# Effects of Exercise Training on Mitochondrial and Capillary Growth in Human Skeletal Muscle: A Systematic Review and Meta-Regression

**DOI:** 10.1007/s40279-024-02120-2

**Published:** 2024-10-10

**Authors:** Knut Sindre Mølmen, Nicki Winfield Almquist, Øyvind Skattebo

**Affiliations:** 1 Section for Health and Exercise Physiology, Inland Norway University of Applied Sciences, P.O. Box. 422, 2604 Lillehammer, Norway; 2 The August Krogh Section for Molecular Physiology, Department of Nutrition, Exercise and Sports, Faculty of Science, University of Copenhagen, Copenhagen, Denmark; 3 Department of Physical Performance, Norwegian School of Sport Sciences, Oslo, Norway

## Abstract

**Background:**

Skeletal muscle mitochondria and capillaries are crucial for aerobic fitness, and suppressed levels are associated with chronic and age-related diseases. Currently, evidence-based exercise training recommendations to enhance these characteristics are limited. It is essential to explore how factors, such as fitness level, age, sex, and disease affect mitochondrial and capillary adaptations to different exercise stimuli.

**Objectives:**

The main aim of this study was to compare the effects of low- or moderate intensity continuous endurance training (ET), high-intensity interval or continuous training (HIT), and sprint interval training (SIT) on changes in skeletal muscle mitochondrial content and capillarization. Secondarily, the effects on maximal oxygen consumption (*V*O_2_max), muscle fiber cross-sectional area, and fiber type proportion were investigated.

**Methods:**

A systematic literature search was conducted in PubMed, Web of Science, and SPORTDiscus databases, with no data restrictions, up to 2 February 2022. Exercise training intervention studies of ET, HIT, and SIT were included if they had baseline and follow-up measures of at least one marker of mitochondrial content or capillarization. In total, data from 5973 participants in 353 and 131 research articles were included for the mitochondrial and capillary quantitative synthesis of this review, respectively. Additionally, measures of *V*O_2_max, muscle fiber cross-sectional area, and fiber type proportion were extracted from these studies.

**Results:**

After adjusting for relevant covariates, such as training frequency, number of intervention weeks, and initial fitness level, percentage increases in mitochondrial content in response to exercise training increased to a similar extent with ET (23 ± 5%), HIT (27 ± 5%), and SIT (27 ± 7%) (*P* > 0.138), and were not influenced by age, sex, menopause, disease, or the amount of muscle mass engaged. Higher training frequencies (6 > 4 > 2 sessions/week) were associated with larger increases in mitochondrial content. Per total hour of exercise, SIT was ~ 2.3 times more efficient in increasing mitochondrial content than HIT and ~ 3.9 times more efficient than ET, while HIT was ~ 1.7 times more efficient than ET. Capillaries per fiber increased similarly with ET (15 ± 3%), HIT (13 ± 4%) and SIT (10 ± 11%) (*P* = 0.556) after adjustments for number of intervention weeks and initial fitness level. Capillaries per mm^2^ only increased after ET (13 ± 3%) and HIT (7 ± 4%), with increases being larger after ET compared with HIT and SIT (*P* < 0.05). This difference coincided with increases in fiber cross-sectional area after ET (6.5 ± 3.5%), HIT (8.9 ± 4.9%), and SIT (11.9 ± 15.1%). Gains in capillarization occurred primarily in the early stages of training (< 4 weeks) and were only observed in untrained to moderately trained participants. The proportion of type I muscle fibers remained unaltered by exercise training (*P* > 0.116), but ET and SIT exhibited opposing effects (*P* = 0.041). *V*O_2_max increased similarly with ET, HIT, and SIT, although HIT showed a tendency for greater improvement compared with both ET and SIT (*P* = 0.082), while SIT displayed the largest increase per hour of exercise. Higher training frequencies (6 > 4 > 2 sessions/week) were associated with larger increases in *V*O_2_max. Women displayed greater percentage gains in *V*O_2_max compared with men (*P* = 0.008). Generally, lower initial fitness levels were associated with greater percentage improvements in mitochondrial content, capillarization, and *V*O_2_max. SIT was particularly effective in improving mitochondrial content and *V*O_2_max in the early stages of training, while ET and HIT showed slower but steady improvements over a greater number of training weeks.

**Conclusions:**

The magnitude of change in mitochondrial content, capillarization, and *V*O_2_max to exercise training is largely determined by the initial fitness level, with greater changes observed in individuals with lower initial fitness. The ability to adapt to exercise training is maintained throughout life, irrespective of sex and presence of disease. While training load (volume × intensity) is a suitable predictor of changes in mitochondrial content and *V*O_2_max, this relationship is less clear for capillary adaptations.

**Graphical Abstract:**

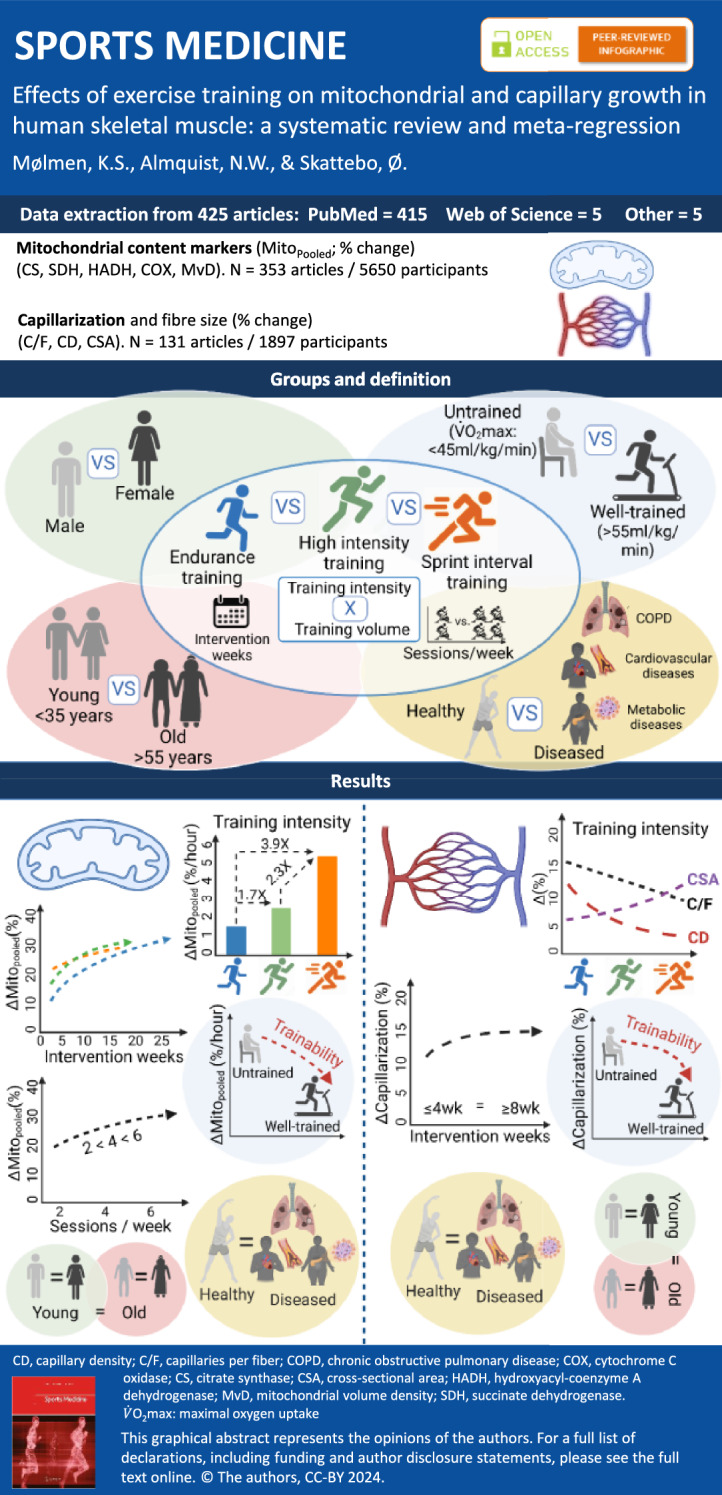

**Supplementary Information:**

The online version contains supplementary material available at 10.1007/s40279-024-02120-2.

## Key Points

**Table:** 

The magnitude of change in mitochondrial content to a given exercise stimulus (trainability) is primarily determined by the initial fitness level and remains consistent throughout life, regardless of sex and the presence of disease.
Greater training volumes and higher exercise intensities are associated with larger increases in mitochondrial content. Thus, training load (intensity × volume) is the most suitable predictor, underlining that higher exercise intensities can compensate for lower training volumes and vice versa.
Increases in mitochondrial content occur in a ~ 2–3:1 ratio with increases in $\dot{V}$O_2_max, and improvements in both capillaries and mitochondrial content are moderately correlated with changes in $\dot{V}$O_2_max.
Most gains in capillarization occur in the early stages of exercise training (< 4 weeks). While ET, HIT, and SIT all increase capillaries per fiber similarly, ET is more effective in increasing capillary density (capillaries per mm^2^) owing to less muscle fiber hypertrophy.

## Introduction

In the last century, major scientific advances have been made in the field of exercise physiology relating to how skeletal muscle capillaries and mitochondria together play essential roles in oxygen and nutrient delivery to the cells, as well as in energy production inside the cells. In this regard, merged evidence from numerous observations have created the widely accepted dogma that repeated exercise sessions (i.e., exercise training) can alter the content of these structures. As early as in 1939–40, the Ukrainian biochemist researcher Olga Chepinoga published the first evidence of exercise training-induced mitochondrial biogenesis [1–3]. The first observation of increased skeletal muscle capillary density with exercise training was published in 1975 by Per Andersen [4].

Mitochondria are organelles responsible for most of the production of adenosine triphosphate, the molecule required for all energy-demanding cellular processes. Given their crucial role in supplying chemical energy, it is no surprise that an increase in mitochondrial content is linked to improved endurance performance but, importantly, it also plays a key role in preventing various chronic diseases and age-related health issues [5–7]. Because of these associations, understanding how mitochondria adapt to different exercise stimuli is imperative for developing evidence-based and effective exercise training prescriptions. Currently, there is an ongoing debate on which exercise training variable—exercise intensity or total training volume—most significantly promotes increases in mitochondrial content [8–11]. Exercise intensity is typically expressed relative to maximal values of e.g., oxygen consumption, heart rate or power output [i.e., maximal oxygen consumption ($\dot{V}$O_2_max), HRmax, and Wmax, respectively], whereas training volume is defined by the total exercise duration (minutes per session *x* training frequency *x* number of intervention weeks) [12–14]. In unraveling the nature of mitochondrial responses to exercise training, a third, arbitrary factor, the training load, might as well explain some of the interindividual differences in adaptations to training. Training load can be defined as the product of relative exercise intensity and the training volume [12–16]. However, the factorial effects of exercise intensity and volume on mitochondrial adaptations remain poorly understood and require further investigation. Furthermore, several other factors may influence the magnitude of change in mitochondrial content in response to exercise training (i.e., the “trainability”), including initial fitness level, age, sex, menopause, degree of active muscle mass engaged during exercise, and the presence of disease [17–21]. Despite indications of their influence, these factors have not yet been systematically reviewed in such a cohort.

Regarding skeletal muscle capillary growth in response to exercise training, several factors, such as menopause and chronic obstructive pulmonary disease (COPD), appear to play influential roles [22, 23]. Interestingly, the stimulus that promotes capillary growth seems to differ somewhat from that which enhances mitochondrial content. Vascular endothelial growth factor (VEGF) is secreted from the muscle fibers to the muscle interstitium during exercise [24] and is recognized as a key regulator of capillary growth [25, 26]. Studies have indicated that high-intensity intermittent exercise leads to lower VEGF levels and subsequently less endothelial cell proliferation compared with low-intensity exercise [27]. Moreover, a longitudinal training study demonstrated a 22% reduction in VEGF levels following eight weeks of high-intensity intermittent exercise [28]. Thus, it has been proposed that prolonged training sessions conducted at a low- to moderate exercise intensity (i.e., exercise training with a relatively high volume) may be more effective in inducing capillary growth [24]. If so, this may be dissimilar to what is important for increasing mitochondrial content which seems to call for a larger portion of the total training volume being performed as high-intensity exercise [24]. Intriguingly, a recent meta-analysis has shown that sprint interval training (SIT) effectively increases capillary content, whereas high-intensity interval training (HIT) does not [29]. However, owing to the limited number of studies included in the analysis (two SIT studies and one HIT study), the results should be interpreted with caution. Therefore, identifying the most effective exercise training factors for improving the vascular bed across different fitness levels, ages, and sexes remains an area requiring further characterization.

The main aim of this systematic review and meta-regression was to examine the effects of low- or moderate-intensity continuous endurance training (ET), HIT, and SIT, on skeletal muscle mitochondrial and capillary content. This evaluation considered variables such as exercise training frequency, number of intervention weeks, initial fitness level, age, sex, presence of disease, and amount of active muscle mass engaged during exercise. Two different approaches were used to analyze the data: (1) analyzing training effects adjusted for the total number of training sessions and (2) evaluating training effects per hour of exercise (“training efficiency”). The first approach emphasizes a high level of ecological validity since, in actual settings, choosing between ET, HIT, and SIT involves selecting different sessions that inherently vary in factors such as minutes per session and the way training is prescribed and conducted. Conversely, the second approach provides insight into which type of exercise intensity yields the most benefit per minute of training (training efficiency). Secondary aims were added to this study and included investigation of the effects of exercise training on whole-body $\dot{V}$O_2_max, muscle fiber cross-sectional area, and fiber type proportion, as well as the relationship between exercise training-induced changes in mitochondrial and capillary content (i.e., peripheral factors of $\dot{V}$O_2_max) to changes in whole-body $\dot{V}$O_2_max.

## Methods

**Fig. 1:**
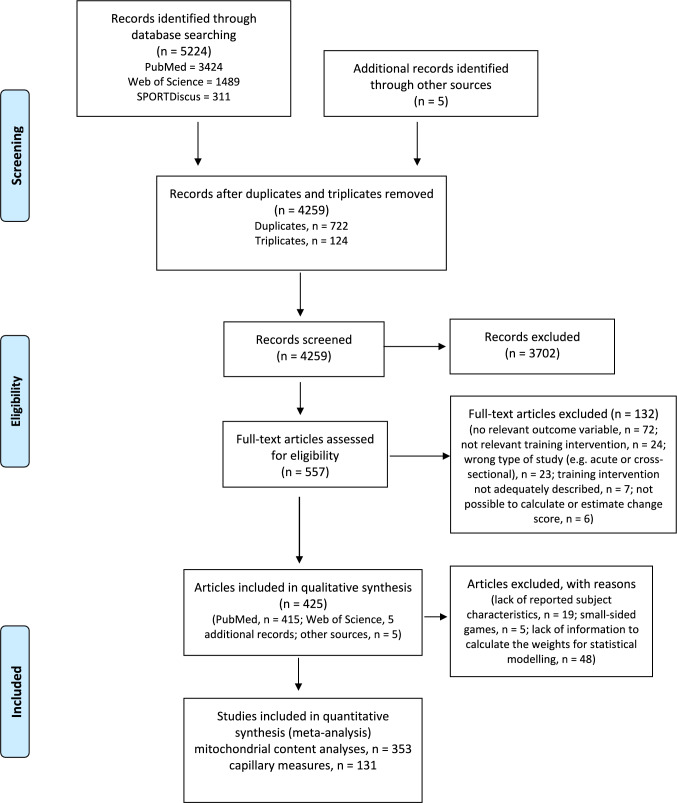
Flowchart of research articles included in the systematic review and meta-regression (PRISMA diagram). PRISMA, Preferred Reporting Items for Systematic Reviews and Meta-Analyses

The present study followed the Preferred Reporting Items for Systematic Reviews and Meta-Analyses (PRISMA) guidelines [30]. A PRISMA diagram displaying the synthesis of the present analysis is presented in Fig. 1.

### Search Strategy

A systematic literature search was conducted in PubMed, Web of Science and SPORTDiscus databases. The search was conducted using the following Boolean operators “AND” and “OR”: (“citrate synthase” OR “cs” OR “cytochrome oxidase” OR “cox” OR “hydroxyacyl” OR “hadh” OR “succinate dehydrogenase” OR “succinic oxidase” OR “succinic dehydrogenase” OR “sdh” OR “oxoglutarate dehydrogenase” OR “ketoglutarate dehydrogenase” OR “ogdh” OR “malate dehydrogenase” OR “mdh” OR “mitochondrial volume” OR “mitochondrial density” OR “mitochondrial content” OR “mitochondrial biogenesis” OR “capillarization” OR “capillary density” OR “capillary supply” OR “capillary content” OR “angiogenesis” AND (“training” OR “exercise”). The search was limited to human studies published in English-written research articles before 2 February 2022. In addition, reference lists of original and review articles were screened to identify additional studies potentially eligible to be included in this systematic review. If a full-text research article was unavailable for the authors, a copy was retrieved via an inter-library loan through the libraries at Inland Norway University of Applied Sciences or the Norwegian School of Sport Sciences.

### Eligibility Criteria

All human studies that investigated the impact of an exercise training intervention on skeletal muscle mitochondrial content or capillarization, using either the “gold-standard” measure of mitochondrial content (transmission electron microscopy), or surrogate measures, such as activity or content of different mitochondrial enzymes, as well as studies with immunohistochemical capillary measurements, were included in the present analysis. The present meta-regression was limited to (1) exercise training intervention studies with a total of ≥ 3 sessions of either ET, HIT, or SIT; (2) research articles that included a description of the training protocol; (3) research articles where the results were reported as average percentage change with standard deviation (SD) from before to after the training intervention, or it was possible to calculate or estimate this through other variables provided [i.e., average absolute changes, average pre- and post-values, standard error (SE), 95% confidence interval (CI) or *t* statistics; calculated or estimated based on the guidelines in the Cochrane Handbook for Systematic Reviews] [31], or if average percentage change with SD was successfully obtained via e-mail contact with the corresponding author of the research article; (4) studies that included at least one relevant mitochondrial or capillary measure.

Only original research articles were included. Study designs that included resistance training in addition to ET, HIT, or SIT (i.e., concurrent training interventions) were excluded. No exclusion criteria were defined related to sex, age, training background, disease status, type of exercise, environmental or dietary manipulations, or which skeletal muscle the biopsies were collected from. For mitochondrial enzymes, both spectrophotometrical and fluorometrical measures of enzyme activity were included, as well as analysis using untreated tissue (wet muscle) and tissue which had been freeze-dried and dissected free of connective tissue, blood, and adipose tissue (dry muscle). Content of mitochondrial enzymes, measured by immunoblotting, was also included. For measurements of mitochondrial density, only studies using transmission electron microscopy for quantification were included and used in further analyses. For capillary measurements, only studies using immunohistochemical analysis of transverse-sectioned muscle biopsies were included. Section 3.1 presents a detailed description and distribution of the data included. On the basis of the above-mentioned inclusion criteria, one reviewer from the author list screened all research articles in the literature search to determine their eligibility. This was done by carefully analyzing titles, abstracts, and full texts of the articles.

### Data Extraction

Two authors collaborated in extracting relevant data from the research articles included. Each of the two authors extracted data from approximately half of the included studies and subsequently double-checked the data the other author had extracted. This process was done twice, giving a quadruple screening of all research articles included. Disagreements were resolved through personal communication between the two authors. Careful screening of the extracted data was conducted with the aim of removing duplicated results presented in distinct research articles. This was done by searching for duplicated values and control-checked by inspecting if variables, such as author names, number of study participants, and training intervention characteristics, were in line across research articles. The following variables were extracted from each research article: author names, year of publication, journal, and PubMed identifier (PMID). For each training group, the following variables were extracted: number of participants, sex, age, body height, initial fitness level, disease (if relevant), exercise training intensity category (i.e., ET, HIT, or SIT), type of exercise, number of training intervention weeks, average number of training sessions per week, exercise intensity, and average number of minutes per training session. Average percentage changes with SD/SE/95% CI, and *P* value, in addition to average pre- and post-values with SD/SE were extracted for the variables: body mass, body mass index (BMI), $\dot{V}$O_2_max (both in absolute values per minute and relative to body mass), enzyme activity, and content of the enzymes citrate synthase (CS), cytochrome c oxidase (COX), hydroxyacyl-coenzyme A dehydrogenase (HADH), succinate dehydrogenase (SDH), oxoglutarate dehydrogenase, malate dehydrogenase, protein content of COX subunit 1, 2, and 4, mitochondrial volume density (MvD, percentage of muscle fiber volume occupied by mitochondria), capillary density (CD, capillaries per mm^2^), capillaries per muscle fiber (C/F), cross-sectional area (CSA) of muscle fibers, and fiber type I proportion. For data in research articles only described in figures or graphs, Fiji software [32] was used for quantification.

### Definitions

#### Training Intensity Categories

Exercise training intensity was reported in several ways across studies. Consequently, three training intensity categories were defined and used in the statistical analyses: (1) low- or moderate intensity continuous endurance training (ET), (2) high-intensity interval or continuous training (HIT), and (3) sprint interval training (SIT). On the basis of previously published guidance about how to determine low-, moderate-, and high-intensity exercise domains [11, 33–36], we defined ET and HIT as exercise training conducted at an intensity below or above the second ventilatory threshold/4 mmol/L blood lactate concentration/87% of HRmax/87% of $\dot{V}$O_2_max/75% of Wmax, respectively. SIT was defined as exercise training protocols that included maximal or near-maximal efforts with a duration of 4–90 s and recovery periods > 1:1. Training interventions which included both ET and HIT were defined as HIT, while training interventions which included both ET and SIT were defined as SIT. Using the percentage of $\dot{V}$O_2_max or HRmax to define exercise intensity is not without limitations from a physiological perspective, as trained individuals typically display their ventilatory and lactate threshold at a higher relative intensity than untrained individuals [37]. The aforementioned guidelines regarding how to determine exercise intensity domains [11, 33–36] are principally most applicable to trained individuals, and untrained individuals may therefore experience that moderate-intensity exercise using % of $\dot{V}$O_2_max or HRmax effort-wise can be considered as high-intensity exercise. Each study was therefore carefully assessed by two of the authors to ensure that the correct training intensity category was used and specifically that the studies with untrained individuals were not misplaced.

#### Initial Fitness Level

Initial fitness level for each of the included training groups was classified into either (1) untrained, (2) moderately trained, or (3) well-trained. This information was mainly retrieved from the research articles' participant description. However, 79 of the studies included did not report such characteristics. For these groups, the fitness level was classified on the basis of their baseline $\dot{V}$O_2_max. For these groups, > 45 and > 55 mL/kg/min were used as cut-off values for moderately trained and well-trained men, respectively. For women, all unclassified training groups had a $\dot{V}$O_2_max  < 35 mL/kg/min and were thus classified as untrained. Of all training groups, 84% had their baseline $\dot{V}$O_2_max measured and the mean ± SD values were 34.8 ± 10.4, 48.8 ± 6.3, and 62.2 ± 6.7 mL/kg/min for untrained, moderately trained, and well-trained participants, respectively (see Table 1 for remaining characteristics).

**Table 1:** Participant and training characteristics for the included data of the pooled mitochondrial analyses (mito_pooled_) (statistical models 1–2)

	Untrained (mean ± SD)	Moderately trained (mean ± SD)	Well-trained (mean ± SD)	Data reported (% of training groups)
Studies (*n*)	237	84	32	–
Training groups (*n*)				
ET	209	47	14	–
HIT	103	44	22	–
SIT	16	38	13	–
Total	328	129	49	–
Participants, total (*n*)				
ET	2659	424	104	–
HIT	1210	370	218	–
SIT	135	383	147	–
Total	4004	1177	469	–
Sex (% men)	74	90	97	100
Disease status (% diseased)	36	0	0	100
Fraction with small muscle mass training (%)	11	22	2	100
Age (years)	39.0 ± 16.5	25.3 ± 9.2	25.2 ± 5.2	98.8
Height (cm)	175.0 ± 5.8	178.1 ± 6.3	179.8 ± 3.7	85.8
Weight (kg)	80.8 ± 12.4	75.4 ± 7.6	73.1 ± 4.6	57.7
$\dot{V}$O_2_max (mL/kg/min)				
ET	35.1 ± 11.3	48.0 ± 6.1	63.7 ± 8.0	80.7
HIT	33.6 ± 8.6	48.2 ± 6.8	63.1 ± 4.9	90.5
SIT	39.3 ± 8.7	50.3 ± 5.7	58.2 ± 7.3	80.6
Mean	34.8 ± 10.4	48.8 ± 6.3	62.2 ± 6.7	84.0
Training weeks (*n*)				
ET	11.4 ± 8.5	6.6 ± 3.9	7.6 ± 7.5	100
HIT	9.0 ± 5.2	5.7 ± 3.2	5.0 ± 4.4	100
SIT	6.5 ± 3.4	4.5 ± 2.3	6.9 ± 9.0	100
Mean	10.4 ± 7.5	5.7 ± 3.4	6.3 ± 6.7	100
Total sessions (*n*)				
ET	42.2 ± 36.5	27.7 ± 17.0	25.5 ± 14.4	97.8
HIT	31.6 ± 19.6	21.6 ± 14.6	21.0 ± 14.7	98.2
SIT	20.9 ± 9.4	14.6 ± 7.5	14.6 ± 12.8	98.5
Mean	37.8 ± 31.7	21.7 ± 14.8	20.3 ± 14.4	98.0
Sessions per week (*n*)				
ET	4.1 ± 1.3	4.3 ± 1.2	5.8 ± 3.3	98.1
HIT	3.8 ± 1.6	3.8 ± 1.8	5.9 ± 3.6	98.8
SIT	3.5 ± 1.1	3.3 ± 0.8	3.3 ± 1.4	98.5
Mean	4.0 ± 1.4	3.8 ± 1.4	5.2 ± 3.2	98.4
Total training hours (*n*)				
ET	35.5 ± 39.0	37.7 ± 37.2	93.2 ± 147.8	96.7
HIT	23.6 ± 16.4	19.9 ± 17.0	29.2 ± 25.5	96.4
SIT	9.5 ± 7.8	6.1 ± 5.3	12.0 ± 20.1	100
Mean	30.5 ± 33.2	22.3 ± 27.8	41.6 ± 84	97.0
Mitochondrial marker (*n* training groups)				
MvD (*n*/% of markers)	54/9%	4/2%	3/3%	–
CS (*n*/% of markers)	247/42%	124/49%	45/47%	–
COX (*n*/% of markers)	115/19%	49/19%	13/14%	–
SDH (*n*/% of markers)	52/9%	19/7%	6/6%	–
HADH (*n*/% of markers)	125/21%	58/23%	29/30%	–
Total (*n*/% of markers)	593/100%	254/100%	96/100%	–
Mitochondrial enzymes measured as:				
Protein content, WB (% of observations)	15.9	24.0	23.7	–
Enzyme activity (% of observations)	84.1	76.0	76.3	–

In total, 353 studies with 506 training groups and a total of 943 observations (i.e., more than one mitochondrial marker was reported in some studies) were used in the statistical models

COX, cytochrome c oxidase/complex IV; CS, citrate synthase; ET, endurance training; HADH, hydroxyacyl-CoA dehydrogenase; HIT, high-intensity interval training; MvD, mitochondrial volume density; SDH, succinate dehydrogenase/complex II; SIT, sprint interval training; $\dot{V}$O_2_max, maximal oxygen consumption; WB, western blot

#### Disease Status

The training groups of each study were categorized into groups comprising (1) healthy participants without any known diseases or conditions, or (2), a group where the participants had a known disease or condition, e.g., diabetes mellitus type I or II, overweight/obesity, chronic heart failure, coronary artery disease, hypertension, COPD, or renal diseases. In subgroup analysis, when comparing disease groups with healthy participants, the following disease groups were used: (1) metabolic diseases [comprising diabetes mellitus type I or II, insulin resistance, metabolic syndrome, and obesity (body mass index > 30 kg/m^2^)], (2) cardiovascular diseases (CVD; comprising participants with hypertension, peripheral arterial occlusive disease, intermittent claudication, coronary artery disease, decreased ejection fraction, and cardiomyopathy), and (3) COPD.

#### Training Time

Total training time for each study was defined as the total active time used to complete all training sessions; specifically, the summative time in each study used for exercise components such as warm-up, main activity of the session, recovery between work periods in interval sessions if active work was performed in these breaks, and cool-down. If time spent on warm-up for HIT or SIT sessions was not specified in the research article, a 15-min warm-up was assumed per session. If the recovery breaks between work periods in interval sessions were not specified to be active or passive, passive breaks were assumed.

#### Small and Large Active Muscle Mass Exercises

Studies were categorized into two levels on the basis of the amount of active muscle mass during exercise: (1) exercises with large active muscle mass, such as bicycling, running, walking, cross-country skiing, and rowing, and (2) exercises with small active muscle mass (one-legged and two-legged knee extension, one-legged cycling, and arm cranking).

### Statistical Analysis

Log-transformed (natural logarithm) fold-changes in mitochondrial content markers from pre- to post-training were analyzed using the generalized linear-mixed model procedure (GLIMMIX) with a specified Gaussian distribution in SAS OnDemand for Academics (SAS Studio 3.8, SAS Institute Inc., Cary, NC). The effect estimates were weighted by the inverse of the square of the studies’ SE (1/SE^2^). When the SE of the fold-change for a training group was missing, it was derived from SD, 95% CI, or *t*-statistics calculated from corresponding exact *P* values if such variables were available instead [38]:

1 $$\text{SE }= \frac{\text{Mean change }}{t}.$$

In cases of no presented variability measure for the fold-change, the SD of the fold-change (SD_change_) was imputed if the mean and SD from pre- (SD_pre_) and post- exercise training (SD_post_) tests for the training group were reported [38]:

2 $${\text{SD}}_{\text{change}} = \frac{\sqrt{{\text{SD}}_{\text{pre}}^{2}+{\text{SD}}_{\text{post}}^{2} - (2 \times r \times {\text{SD}}_{\text{pre}} \times {\text{SD}}_{\text{post}})} }{{\text{Mean}}_{\text{pre}}}.$$

In this calculation, the correlation coefficient (*r*) is describing the similarity between the pre- and post- exercise training data across participants. This was calculated in each research study reporting SD_pre_, SD_post_, and SD_change_, and the median value was subsequently used in Eq. 2. Mitochondrial content markers (i.e., CS, HADH, COX, SDH activity or content, and MvD) displayed an *r* = 0.61 (calculation based on 105 research studies), while capillary markers (i.e., CD and C/F): *r* = 0.71 (based on 31 studies):

3 $$r = \frac{ {\text{SD}}_{\text{pre}}^{2} + {\text{SD}}_{\text{post}}^{2} - {\text{SD}}_{\text{change}}^{2} }{{2 \times {\text{SD}}_{\text{pre}} \times \text{ SD}}_{\text{post}}}.$$

For clarity, Supplementary Information 1 summarizes the characteristics of each statistical model included in the study (model number, dependent variable and its form, included fixed- and random effects, training groups, and participants), and in which figure the resultant effect estimates are presented. Data are expressed as means ± 95% CI, unless otherwise specified.

#### Modelling Mitochondrial Data

**Fig. 2:**
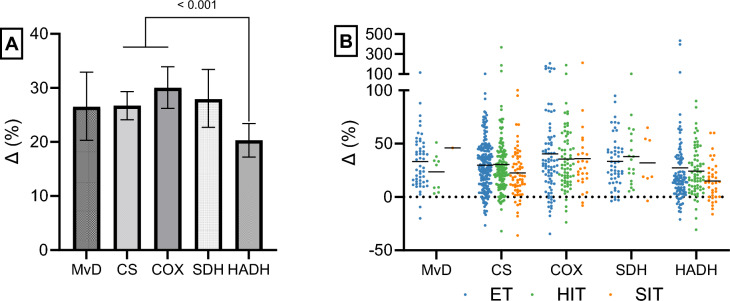
The change (Δ) in mitochondrial volume density (MvD), citrate synthase (CS), cytochrome c oxidase/complex IV (COX), succinate dehydrogenase (SDH), and hydroxyacyl-CoA dehydrogenase (HADH) from before to after training. In 2A, the estimated marginal means with 95% confidence limits from model 1 is presented, which included 353 studies, 506 training groups, 5650 participants, and 943 observations of mean changes (MvD: *N* = 61; CS: *N* = 416; COX: *N* = 177; SDH: *N* = 77; HADH: *N* = 212). In 2B, the individual raw values are presented divided into training intensity categories (ET, endurance training; HIT, high-intensity interval training; SIT, sprint interval training) and mitochondrial content markers

For the mitochondrial objective, statistical models were constructed using the log-transformed fold-change in mitochondrial content markers as the dependent variable, if not otherwise specified. In model 1, the fixed effect of mitochondrial content marker was included (five levels: MvD, CS, SDH, COX, and HADH) to test whether these markers responded differently to exercise training (see Fig. 2A). This model also included random effects of study ID and training group ID. For the remaining statistical models, the five mitochondrial content markers were pooled (Mito_pooled_) to improve statistical power and used as the weightiest stand-alone measure of mitochondrial content. For clarity, some of the below statistical models were also run isolated for CS, i.e., the mitochondrial marker with most data points in the current quantitative synthesis. This CS-only analysis yielded comparable results to Mito_pooled_ which are presented in Supplementary Information 2. The other mitochondrial content markers defined in literature search (oxoglutarate and malate dehydrogenase) were excluded prior to statistical analyses owing to low power (not enough data points for these variables). Model 2 included the fixed effect of training intensity category, did not include any random effects, and used raw percent change scores as the dependent variable (unadjusted fixed effect model). Model 3 included the fixed effects of training intensity category (three levels, SIT, HIT, and ET), training intensity category interacted by the log-transformed (natural logarithm) number of training intervention weeks (continuous, giving three slopes), initial fitness level (three levels: untrained, moderately trained, and well-trained individuals), the log-transformed number of training sessions per week (continuous, giving one slope), active muscle mass during exercise (two levels, small and large active muscle mass exercises; see Sect. 2.4.5 for explanation), sex (three levels: men, women, and mixed; mixed, groups comprising both men and women), disease status (two levels, healthy and diseased participants), and age (three levels, ≤ 35 years, > 35–55 years and > 55 years). To further test the impact of different disease groups, model 4 included an interaction between disease group (four levels, healthy, metabolic diseases, CVD, and COPD) and age (two levels, ≤ 35 years and > 55 years; those > 35–55 years were excluded). The above model also included the fixed effect of training intensity category, an interaction between training intensity category and the log-transformed number of training intervention weeks (giving three slopes) and the log-transformed number of training sessions per week (giving one slope), and the model was only run on data from previously untrained participants. These decisions were made owing to the findings in model 3 (i.e., to account for covariates that had a significant effect). To test the impact of menopause on training responses, model 5 included the interaction between sex (two levels, men and women; the mixed sex group was excluded) and age (two levels, ≤ 35 years and > 55 years, where those > 35–55 years were excluded to avoid groups including both pre- and postmenopausal women), and included the same covariate adjustments as in model 4. This model was only run on data from untrained, healthy participants. In model 6, the dependent variable was the fold-change in mitochondrial content divided by the total number of hours of exercise (a data step carried out before modeling), and the model included the interaction effect between initial fitness level and training intensity category (nine levels, 3 × 3). Hence, in models 3–5, the change in mitochondrial content was adjusted for the total number of training sessions, while a normalization for the total hours of exercise was carried out for model 6 to compare training efficiency between training intensity categories. All models included the random effects of study ID, training group ID, and mitochondrial content marker (five levels, CS, COX, HADH, SDH, and MvD) to allow for different effect estimates between studies, between different groups of participants within the same study and between mitochondrial content markers. Estimated marginal means ± 95% CI were back-transformed and expressed as percentage changes and percentage changes per hour of exercise training. For models including several classification effects, estimated marginal means for each classification effect were calculated based on the observed weight of the other classification effects and the mean value of continuous fixed effects (the OBSMARGINS option within the LSMEANS statement or weighted the same way within the ESTIMATE statement) owing to the unbalanced des**i**gn. The impact of continuous fixed effects on the dependent variable was estimated and graphed from the 10th to the 90th percentile of its frequency distribution. For the impact of intervention weeks on mito_pooled_, this implicated plotting the estimated marginal means from 2–11 weeks, 2–13 weeks and 2–23 weeks of training for SIT, HIT, and ET, respectively. Similarly, the estimated marginal means for 2–6 sessions/week was graphed. Only planned pairwise comparisons were conducted using the ESTIMATE statement, and the Holm–Bonferroni method was used to adjust* P* values, while the Bonferroni method was used to adjust 95% CI for multiple comparisons in a row-wise and column-wise fashion.

#### Modelling of Muscle Capillary and Fiber Cross-Sectional Area Data

Separate models for log-transformed fold-changes in C/F (model 7), CD (model 8) and muscle fiber CSA (model 9) were constructed. Models 7–9 included the fixed effects of training intensity category, number of training intervention weeks (categorical with three levels, ≤ 4 weeks, > 4–8 weeks and > 8 weeks), and initial fitness level. No interaction term was specified between the fixed factors owing to a lack of data points with a related not-satisfactory statistical power for these variables, especially concerning SIT and well-trained individuals. Additionally, the number of training intervention weeks was modeled using the number of weeks as a continuous variable in both its raw and log-linear form. However, these approaches yielded worse or similar statistical model fits compared with the categorical fixed effect approach, and therefore, they were not used in our final analyses. To test the impact of different disease groups on changes in C/F, model 10 included the interaction between disease group (four levels, healthy, metabolic diseases, CVD, and COPD) and age (two levels, ≤ 35 years and > 55 years; those > 35–55 years were excluded), fixed effects of intervention weeks (categorical with three levels, ≤ 4 weeks, > 4–8 weeks and > 8 weeks) and training frequency (categorical with two levels, ≤ 3 sessions/week, > 3 sessions/week), and was only run on data from previously untrained participants. To test the impact of menopause on training-induced changes in C/F, model 11 included the interaction between sex (two levels, men and women; the mixed sex group was excluded) and age (two levels, ≤ 35 years and > 55 years; those > 35–55 years were excluded to avoid groups including both pre- and postmenopausal women), and was adjusted for intervention weeks and training frequency in the same way as model 10. This model was only run on data from untrained, healthy participants. All models included random effects for study ID and training group ID, allowing calculation of effect estimates between studies, as well as effect estimates between different groups of participants within the same study. The residual variance was set to unity as each training group ID only had one data point each in the model. Estimated marginal means ± 95% CI were back-transformed and expressed as percentage changes, and estimated marginal means for each classification effect were calculated on the basis of the observed weight of the other classification effects. For separate analyses of absolute changes in CD, C/F, and muscle fiber CSA, see Supplementary Information 3.

#### Muscle Fiber Type Proportion

The proportion of muscle fiber type I was analyzed using the generalized linear-mixed model procedure with a specified Gaussian distribution. Model 12 included the fixed effect interaction between time point (two levels: pre- and post-training) and training intensity (three levels). The model included random effects for study ID and training group ID, allowing calculation of effect estimates between studies, as well as effect estimates between different groups of participants within the same study. Effect estimates were weighted by the inverse of the square of the studies’ SE (1/SE^2^) in baseline fiber type I proportion. Pairwise within- and between group pre- to post-training differences were analyzed using the ESTIMATE statement, and the Holm–Bonferroni method was used to adjust *P* values, while the Bonferroni method was used to adjust 95% CI for multiple comparisons.

#### Maximal Oxygen Consumption

The statistical models analyzing the change in mito_pooled_ (models 2–6) were also used to analyze the change in $\dot{V}$O_2_max for training interventions using a large active muscle mass while training (studies on small muscle mass exercises were excluded), except using the following changes to the models: (1) the dependent variable was the log-transformed fold change in $\dot{V}$O_2_max and fold change in $\dot{V}$O_2_max per hour of exercise training, (2) the models included random effects for study ID and training group ID, and (3) the residual variance was set to unity as each training group ID only had one data point each in the model. These models were named chronologically in the same order as models for mito_pooled_ (models 13–17). For separate analyses of absolute changes in $\dot{V}$O_2_max, see Supplementary Information 3.

#### Correlation Analysis

Pearson product-moment correlation was used to assess the strength of the linear association between the percentage changes in $\dot{V}$O_2_max, mitochondrial content markers, C/F, and CD from before to after training.

## Results

### Study Characteristics

The PubMed, Web of Science and SPORTDiscus literature search identified in total 5224 records (Fig. 1). Five research articles were additionally included, resulting in a total of 5229 records. After removing duplicated and triplicated records, 4259 research articles remained, of which 557 passed the initial title and abstract screening. Full-text articles were retrieved for these 557 records, and of these, 132 records were excluded for not conforming with the eligibility criteria of this review. Finally, data from 425 research articles were included in the qualitative synthesis of the review (for reference lists, see Supplementary Information 4 and 5 for research articles used in mitochondrial and capillary analysis, respectively).

#### Description of Mitochondrial Data

Data from 353 studies were included in statistical models analyzing changes in mitochondrial content (models 1–2). The included studies of these analyses comprised a total of 5650 participants from 506 different training groups. In Table 1, participant- and training characteristics divided into subcategories of initial fitness level (untrained, moderately trained, and well-trained individuals) and training intensity categories (ET, HIT, and SIT), are presented. The exercise types within the training groups were bicycling (*n* = 313), running/walking (*n* = 73), combined program involving running, bicycling, and other endurance-based activities (*n* = 44), small muscle mass exercises (*n* = 67), cross-country skiing (*n* = 4), swimming (*n* = 2), and other activities/not reported (*n* = 3). The biopsies were taken from vastus lateralis (*n* training groups = 492), gastrocnemius (*n* = 11), rectus femoris (*n* = 1), triceps brachii (*n* = 1), and deltoideus (*n* = 1). The changes in MvD, CS, SDH, COX, and HADH from before to after exercise training were assessed in 61, 416, 77, 177, and 212 training groups, respectively (943 observations in total). Of the mitochondrial enzymes (CS, SDH, COX, and HADH), 81% and 19% of the observations were enzyme activity and protein content measurements, respectively.

#### Description of Capillary Data

The number of training groups and participants included in the models analyzing C/F, CD and fiber CSA are presented in Table 2. In the same table are mean values ± SD of C/F, CD and fiber CSA for the untrained, moderately trained and well-trained participants included in the analyses presented, as well as $\dot{V}$O_2_max values for the described groups.

**Table 2:** The number of training groups and participants (*N*/*N*) included in the models analysing capillary-to-fiber ratio (C/F), capillary density (CD, capillaries per mm^2^) and muscle fiber cross-sectional area (CSA, μm^2^) with participant (divided in training status groups) and training (divided by training intensity categories) characteristics

	Capillary-to-fiber ratio	Capillary density	Cross-sectional area
Total training groups/participants (*N*/*N*)	153/1825	141/1897	76/886
Training intensity			
ET (*N*/*N*)	93/1103	87/1241	46/528
HIT (*N*/*N*)	50/618	43/524	27/320
SIT (*N*/*N*)	10/104	11/132	3/38
Training status			
Untrained (*N*/*N*)	117/1491	104/1542	64/769
Moderately trained (*N*/*N*)	23/229	26/275	9/91
Well-trained (*N*/*N*)	13/105	11/80	3/26
Training intervention duration			
≤ 4 weeks (*N*/*N*)	22/189	26/242	7/58
4–8 weeks (*N*/*N*)	61/654	51/565	35/378
> 8 weeks (*N*/*N*)	70/982	64/1090	34/450
Age (years; mean ± SD)			
Untrained	43.5 ± 16.1	42.4 ± 16.3	40.8 ± 16.5
Moderately trained	31.0 ± 13.1	29.9 ± 12.7	33.3 ± 16.4
Well-trained	23.7 ± 3.7	22.5 ± 2.2	22.8 ± 1.0
Height (cm; mean ± SD)			
Untrained	175.1 ± 5.5	175.6 ± 5.3	175.7 ± 5.2
Moderately trained	179.0 ± 6.6	178.9 ± 5.9	176.0 ± 6.1
Well-trained	179.6 ± 2.7	179.7 ± 2.8	No data
Weight (kg; mean ± SD)			
Untrained	78.2 ± 11.0	79.6 ± 11.0	79.6 ± 11.3
Moderately trained	76.8 ± 5.7	76.0 ± 5.9	75.2 ± 8.4
Well-trained	74.2 ± 3.8	73.7 ± 3.7	73.1 ± 6.4
$\dot{V}$O_2_max (mL/kg/min) (mean ± SD)			
Untrained	31.2 ± 10.9	32.1 ± 10.7	33.7 ± 10.5
Moderately trained	50.5 ± 8.4	49.1 ± 7.4	45.5 ± 14.5
Well-trained	60.5 ± 7.6	61.8 ± 8.5	No data
C/F, CD and CSA (mean ± SD)			
Untrained	1.67 ± 0.50	386 ± 145	4568 ± 1154
Moderately trained	2.57 ± 1.13	384 ± 78	5170 ± 1375
Well-trained	3.01 ± 1.30	437 ± 98	4340 ± 566
Training weeks (*n*; mean ± SD)			
ET	11.4 ± 8.3	12.5 ± 9.7	9.6 ± 5.6
HIT	10.6 ± 4.7	10.0 ± 4.0	10.7 ± 4.8
SIT	3.4 ± 2.2	3.6 ± 2.3	6.3 ± 2.5
Total sessions (*n*; mean ± SD)			
ET	39.9 ± 30.2	44.7 ± 34.1	34.0 ± 16.1
HIT	32.2 ± 15.3	30.4 ± 13.7	31.6 ± 14.8
SIT	9.3 ± 4.8	10.6 ± 6.1	18.3 ± 4.7
Sessions per week (*n*; mean ± SD)			
ET	4.0 ± 1.6	4.1 ± 1.6	4.1 ± 2.0
HIT	3.1 ± 0.5	3.1 ± 0.6	3.0 ± 0.4
SIT	2.8 ± 0.8	3.0 ± 0.9	2.7 ± 1.2
Total training hours (*n*; mean ± SD)			
ET	41.0 ± 49.4	44.1 ± 44.4	25.5 ± 14.9
HIT	26.0 ± 15.3	29.2 ± 34.8	25.1 ± 16.3
SIT	3.0 ± 1.1	2.7 ± 1.0	3.5 ± 1.7

ET, endurance training; HIT, high-intensity interval training; SIT, sprint interval training; $\dot{V}$O_2_max, maximal oxygen consumption

### Primary Analyses

#### Comparison of Responses to Exercise Training Between Mitochondrial Markers

In general, MvD, CS, COX, SDH, and HADH increased from before to after exercise training by 20–30% (all, *P* < 0.001; Fig. 2A; model 1), with the increase being slightly lower for HADH (20.3 ± 3.1%) compared with CS and COX (27–30%, *P* < 0.001). In Fig. 2B, the individual changes for all training groups and mitochondrial markers included in model 1 are presented. Unadjusted for covariates, not log-transformed, non-weighted, and pooled with a fixed effect model, the mean changes in the mitochondrial markers were 31.7 ± 3.1%, 30.6 ± 3.8%, and 23.7 ± 5.9% for ET, HIT, and SIT, respectively (model 2). However, differences in the total time invested in exercise training were observed across intensity categories, with participants averaging 38.6 ± 6.1 h of ET, 23.3 ± 2.8 h of HIT, and 8.0 ± 2.6 h of SIT.

#### The Effect of Exercise Training on Muscle Mitochondrial Content

**Fig. 3:**
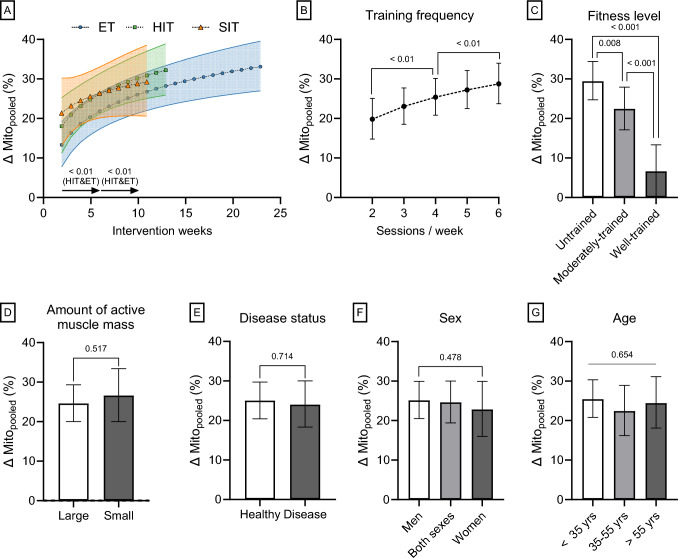
The effects of intervention weeks by training intensity category (**A**), training frequency (**B**), initial fitness level (**C**), amount of recruited muscle mass during exercise (**D**), disease status (**E**), sex (**F**), and age (**G**) on training-induced changes (Δ) in mito_pooled_ (model 3). The arrows in panel (**A**) indicate significant increases between 2-6 weeks and 6-10 weeks of training. Mito_pooled_, a pooled measure of the five mitochondrial content markers mitochondrial volume density, citrate synthase, succinate dehydrogenase, cytochrome c oxidase/complex IV and hydroxyacyl-CoA dehydrogenase; ET, endurance training; HIT, high-intensity interval training; SIT, sprint interval training. Values are estimated marginal means with 95% confidence limits [*N* studies = 343, *N* training groups = 496, *N* participants = 5511, *N* observations of mean changes = 928 (MvD: *N* = 60; CS: *N* = 409; SDH: *N* = 76; COX: *N* = 177; HADH: *N* = 206)]

Using pooled data from the five mitochondrial content markers (mito_pooled_; comprising MvD, CS, SDH, COX, and HADH measurements), and adjusting for covariates (intervention weeks, training frequency, initial fitness level, active muscle mass while training, disease status, sex, and age) with appropriate weighting, all training intensity categories displayed significant increases in mitochondrial content (ET, 22.7 ± 4.6%; HIT, 27.0 ± 5.1%; SIT, 27.0 ± 6.7%; all *P* < 0.001; model 3). Of note, the estimated marginal means comparing the training intensity categories by training intervention weeks differ slightly from the mean changes presented in Sect. 3.2.1 owing to weighting, log-transformation before modelling, and adjustments for covariates, particularly because SIT studies were on average shorter and displayed lower training frequency (see Table 1). When studying the time-course of adaptation, all training intensity categories increased mito_pooled_ after only 2 weeks of training (ET, 13.5 ± 5.7%; HIT, 18.7 ± 7.0%; SIT, 21.5 ± 8.6%; all *P* < 0.001; Fig. 3A; model 3). The increase in mito_pooled_ over intervention weeks followed a log-linear relationship, with ET and HIT showing significant increases between 2–6 weeks and 6–10 weeks of training (all, *P* < 0.01). However, this increase was not significant for SIT (*P* = 0.652; Fig. 3A). Training frequency exerted a log-linear impact on mito_pooled,_ with six sessions/week showing greater potency than two and four sessions/week (both *P* < 0.01; Fig. 3B; model 3). Mito_pooled_ increased with exercise training irrespective of initial fitness level, including in well-trained participants (6.6 ± 6.5%; *P* = 0.046; Fig. 3C). However, mito_pooled_ increased to a greater extent in previously moderately trained participants compared to well-trained participants (mean difference: Δ 14.7 ± 8.6% points; *P* < 0.001; Fig. 3C), and the largest improvements were observed in previously untrained participants (untrained versus moderately trained: Δ 5.8 ± 5.4% points; *P* = 0.008; Fig. 3C; model 3). Mito_pooled_ increased similarly with exercise training engaging whole-body exercises and exercises targeting small muscle groups (Fig. 3D; *P* = 0.512; model 3). The exercise training response was not affected by disease status (*P* = 0.714; Fig. 3E; model 3), sex (*P* = 0.478; Fig. 3F; model 3), or age (*P* = 0.654; Fig. 3G; model 3). When stratified by disease groups, neither young participants (< 35 years) with metabolic diseases (*P* = 0.704) nor older participants (> 55 years) with metabolic diseases (*P* = 0.512), CVD (*P* = 1.00), or COPD (*P* = 1.00) responded differently to exercise training compared to healthy, age-matched, and initial fitness level-matched (i.e., only untrained) participants, when controlling for training intensity, frequency, and intervention weeks (Table 3; model 4). Furthermore, older, healthy, untrained women (*P* = 0.824) and men (*P* = 0.818) increased mito_pooled_ to a similar extent as their younger peers after adjustment for training intensity, frequency, and intervention weeks (Table 3; model 5).

**Table 3:** The interaction between age and disease group, and age and sex, on training-induced changes (Δ) in mito_pooled_ (statistical models 4 and 5, respectively), capillary-to-fiber ratio (statistical models 10 and 11, respectively), and maximal oxygen consumption ($\dot{V}$O_2_max) (statistical models 15 and 16, respectively) adjusted for training intensity category, training frequency, and the interaction between training intensity category and intervention weeks

	ΔMito_pooled_ (%) (statistical model nr.)	ΔCapillary-to-fiber ratio (%) (statistical model nr.)	Δ$\dot{V}$O_2_max (%) (statistical model nr.)	*N* training groups
Healthy versus disease by age	(model 4)	(model 10)	(model 15)	
Young, healthy	30.4 ± 5.1	17.5 ± 4.4	13.1 ± 1.4	(259/40/83)
Young, metabolic disease	28.1 ± 11.8	9.9 ± 12.5	14.8 ± 4.1	(28/4/12)
Old, healthy	26.0 ± 8.6	18.3 ± 6.8	12.2 ± 2.3	(49/13/28)
Old, metabolic disease	36.4 ± 13.7	23.8 ± 15.9	11.5 ± 4.0	(21/3/9)
Old, CVD	27.3 ± 10.7	9.7 ± 9.1	10.8 ± 3.7	(30/8/10)
Old, COPD	30.5 ± 20.4	10.2 ± 10.6	6.0 ± 7.3	(9/7/2)
Sex × age	(model 5)	(model 11)	(model 16)	
Young, men	32.0 ± 6.7	20.5 ± 4.8	12.6 ± 1.4	(201/33/66)
Young, women	29.6 ± 13.2	16.2 ± 17.9	18.7 ± 4.1*	(17/2/6)
Old, men	30.6 ± 12.2	16.9 ± 7.9	11.6 ± 4.1	(26/9/17)
Old, women	27.2 ± 18.4	14.5 ± 13.2	13.1 ± 4.3^†^	(9/3/5)

Only previously untrained participants were used for the analysis of disease status, while the analysis of sex by age included data exclusively from untrained, healthy participants. Numbers in parentheses in the rightmost column denote the number of training groups in each subgroup for mito_pooled_/capillary-to-fiber ratio/$\dot{V}$O_2_max. Detailed information on the number of studies and participants is provided in Supplementary Information 1: Summary of statistical models

Mito_pooled_, a pooled measure of the five mitochondrial content markers of mitochondrial volume density, citrate synthase, succinate dehydrogenase, cytochrome c oxidase/complex IV and hydroxyacyl-CoA dehydrogenase; COPD, chronic obstructive pulmonary disease; CVD, cardiovascular diseases. Values are estimated marginal means with 95% confidence limits

*Denote significant differences between sex at specific age (*P* = 0.004)

^†^Denote tendency to differences between age groups within sex (*P* = 0.060)

##### Change in Mitochondrial Content Per Hour of Exercise Training (Training Efficiency)

**Fig. 4:**
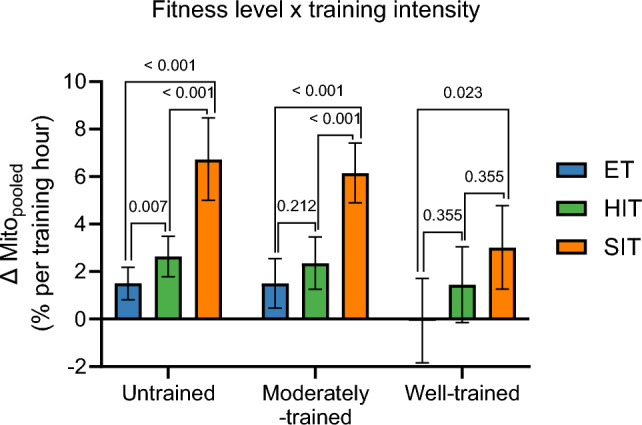
The interaction effect of initial fitness level and training intensity category on changes (Δ) in mito_pooled_ per training hour (model 6). Mito_pooled_, a pooled measure of the five mitochondrial content markers mitochondrial volume density, citrate synthase, succinate dehydrogenase, cytochrome c oxidase/complex IV, and hydroxyacyl-CoA dehydrogenase. ET, endurance training; HIT, high-intensity interval training; SIT, sprint interval training. Values are estimated marginal means with 95% confidence limits. [*N* studies = 345, *N* training groups = 491, *N* participants = 5488, *N* observations = 918 (MvD: *N* = 59; CS: *N* = 404; SDH: *N* = 75; COX: *N* = 174; HADH: *N* = 206)]

When normalizing the percentage change in mito_pooled_ to total hours of exercise training (model 6) and calculating as a weighted mean across all initial fitness level groups, SIT was ~ 2.3 times more efficient than HIT and ~ 3.9 times more efficient than ET (both *P* < 0.001), while HIT was ~ 1.7 times more efficient than ET (*P* = 0.004). This trend of increasing efficiency for ET < HIT < SIT was consistent when stratifying the dataset by initial fitness level groups (Fig. 4; model 6). Untrained and moderately trained individuals increased mito_pooled_ per hour of exercise training for all training intensity categories (all, *P* < 0.01). However, well-trained individuals did not exhibit any change in mito_pooled_ when expressed per hour of exercise training after ET (*P* = 0.933) and HIT (*P* = 0.075), but they significantly increased after SIT (*P* < 0.001).

#### The Effect of Exercise Training on Muscle Capillarization

**Fig. 5:**
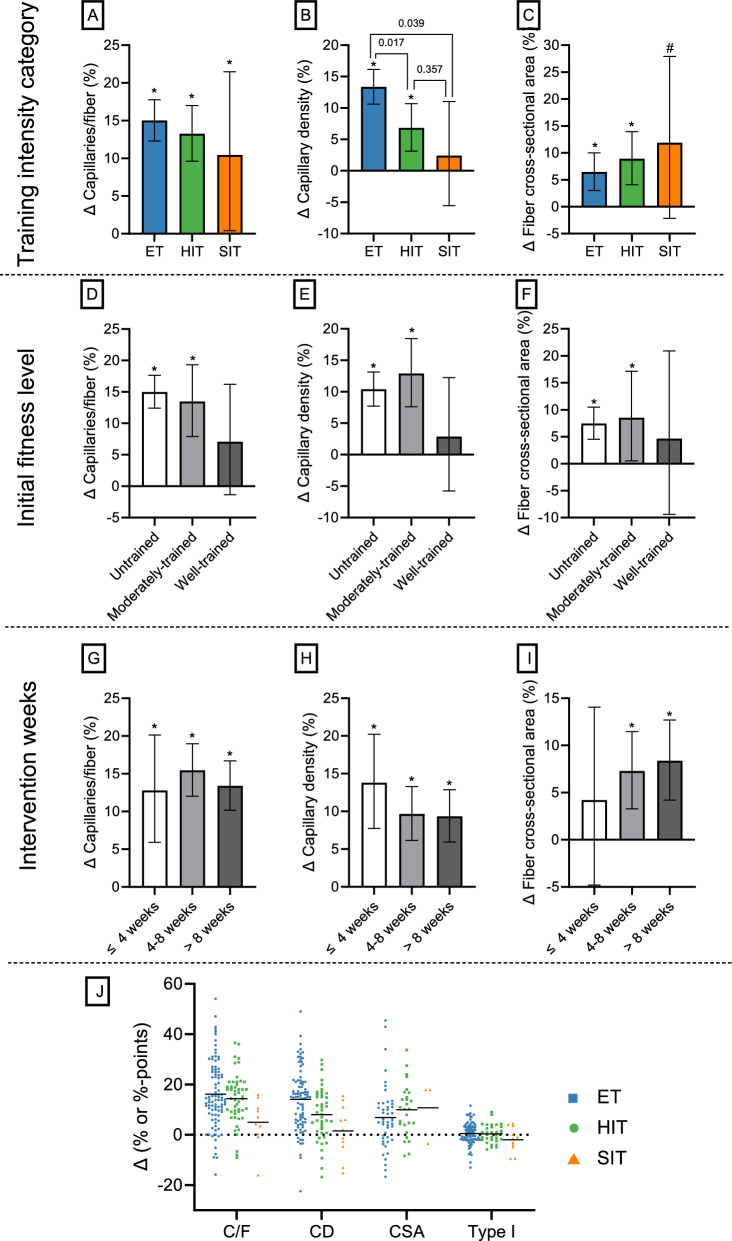
Change (Δ) in capillary-to-fiber ratio (model 7), capillary density (model 8), and muscle fiber cross-sectional area (model 9) in response to exercise training. In the upper panel, the effects of training intensity on training-induced changes in capillary-to-fiber ratio (**A**), capillary density (**B**), and muscle fiber cross-sectional area (**C**) are shown. In the second panel (**D**–**F**), the effects of initial fitness level are displayed, while in the third panel (**G**–**I**), the impact of the number of training intervention weeks on the same variables are presented. In **J**, the individual changes (Δ) in capillary-to-fiber ratio (*C*/*F*), capillary density (CD), muscle fiber cross-sectional area (CSA), and type I fiber distribution from before to after a period of ET, HIT and SIT. For the number of training groups and participants, see Table 2. Values are estimated marginal means with 95% confidence limits. Significant change (*P < 0.05) and tendency to change (^#^*P* < 0.10) from baseline. ET, endurance training; HIT, high-intensity interval training; SIT, sprint interval training

Absolute changes in C/F and CD are detailed in Supplementary Information 3 and are presented here if diverging from the percentage change results. C/F increased after ET (15.0 ± 2.7%; *P* < 0.001), HIT (13.3 ± 3.7%; *P* < 0.001), and SIT (10.4 ± 10.5%; *P* = 0.041), with no significant differences between the exercise intensity categories (*P* = 0.556; Fig. 5A; model 7). CD increased only after ET (13.3 ± 2.8%; *P* < 0.001) and HIT (6.8 ± 3.8%; *P* < 0.001), with ET showing a significantly larger increase compared with both HIT (Δ 5.7 ± 4.8% points; *P* = 0.017) and SIT (Δ 9.6 ± 9.4% points; *P* = 0.039; Fig. 5B; model 8). These estimated marginal means are slightly different from the mean of the training groups’ individual changes for C/F and CD (Fig. 5J) owing to covariate adjustments (initial fitness level and training intervention weeks), log-transformation before modeling, and appropriate weighting. C/F increased in previously untrained (15.0 ± 2.6%; *P* < 0.001) and moderately trained participants (13.5 ± 5.7%; *P* < 0.001), while well-trained participants only tended to increase C/F in response to exercise training (7.1 ± 8.8%; *P* = 0.102; Fig. 5D). Similarly, exercise training increased CD (Fig. 5E) in both untrained (10.4 ± 2.7%; *P* < 0.001) and moderately trained individuals (12.9 ± 5.4%; *P* < 0.001), but not in well-trained individuals (2.8 ± 9.0%; *P* = 0.525). After only ≤ 4 weeks of exercise training, increases in C/F (12.8 ± 7.1%; *P* < 0.001) and CD (13.8 ± 6.2%; *P* < 0.001) were observed, with no further increases associated with a greater number of training intervention weeks (Fig. 5G, H; *P* > 0.73). The increase in C/F did not significantly differ between healthy participants and those with metabolic diseases, CVD or COPD (all comparisons, *P* > 0.254; Table 3; model 10). However, when analyzing absolute changes (Δ C/F; see Supplementary Information 3), C/F tended to increase less in young individuals with metabolic diseases (*P* = 0.072) and older participants with COPD (*P* = 0.051) compared with their age-matched healthy counterparts (Supplementary Information 3, Fig. S5A). Both male and female participants > 55 years of age increased C/F in a comparable manner to their younger counterparts (*P* = 0.450 and *P* = 0.866, respectively; Table 3; model 11).

### Secondary Analyses

#### The Effect of Exercise Training on Muscle Fiber Cross-Sectional Area and Type I Proportion

**Fig. 6:**
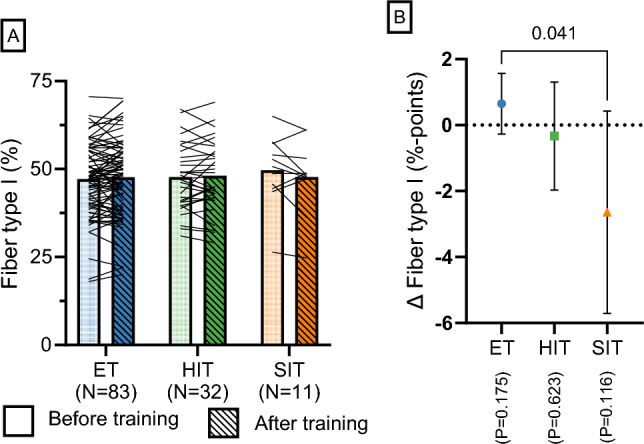
**A** Presents the average type I fiber distribution *per* training group before and after training, and **B** presents the mean change with 95% confidence limits (model 12). For the number of training groups and participants, see Table 2

Exercise training increased muscle fiber CSA (ET: 6.5 ± 3.5%, *P* < 0.001; HIT: 8.9 ± 4.9%, *P* < 0.001; SIT: 11.9 ± 15.1%, *P* = 0.099; Fig. 5C; model 9). This effect was numerically, but not statistically, largest for SIT (likely owing to limited number of SIT studies; see Fig. 5J for the individual data). Untrained and moderately trained participants increased their muscle fiber CSA by 7.5 ± 3.0% (*P* < 0.001) and 8.5 ± 8.3% (*P* = 0.037), respectively, whereas well-trained participants did not experience any significant change (4.7 ± 15.2%; *P* = 0.527; Fig. 5F). Significant increases in muscle fiber CSA were observed after 4–8 weeks of exercise training (7.3 ± 4.1%; *P* < 0.001), with no further increases displayed with larger number of training intervention weeks (4–8 weeks versus > 8 weeks: *P* = 1.00; Fig. 5I). Regarding muscle fiber type proportion, no significant changes in type I fiber proportion were found following exercise training (Fig. 6A, B). Specifically, the changes were 0.7 ± 0.9% points for ET (*P* = 0.175), − 0.3 ± 1.6% points for HIT (*P* = 0.623) and − 2.6 ± 3.1% points for SIT (*P* = 0.116; model 16). However, the pre- to post-test change was different between ET and SIT (3.3 ± 3.2% points; *P* = 0.041), due to a numerical increase for ET and a numerical decrease for SIT. Individual raw changes for muscle fiber CSA and type I muscle fiber proportion per training group are presented in Fig. 5J.

#### The Effect of Exercise Training on Maximal Oxygen Consumption

**Fig. 7:**
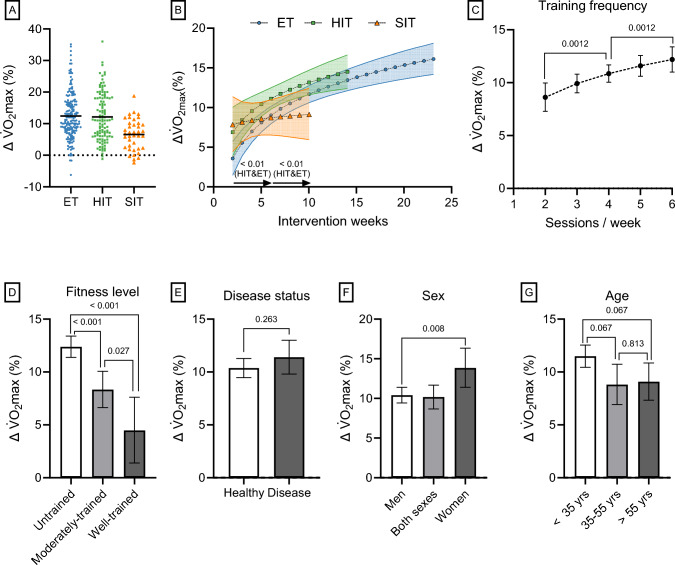
In **A**, the unadjusted individual raw changes in maximal oxygen consumption ($\dot{V}$O_2_max) are presented divided into training intensity categories (ET, endurance training; HIT, high-intensity interval training; SIT, sprint interval training). The remaining figures show the effects of intervention weeks by training intensity category (**B**), training frequency (**C**), initial fitness level (**D**), disease status (**E**), sex (**F**), and age (**G**), on training-induced changes (Δ) in $\dot{V}$O_2_max after adjusting for covariates (model 14). Values are estimated marginal means with 95% confidence limits (*N* studies = 202, *N* training groups = 298, *N* participants = 3524)

Absolute changes in $\dot{V}$O_2_max are detailed in Supplementary Information 3 and are presented here if deviating from the percentage change results. Figure 7A presents the individual changes in $\dot{V}$O_2_max for all training groups, divided into training intensity categories. Unadjusted for covariates and pooled with a fixed effect model (model 13), the mean changes in $\dot{V}$O_2_max were 12.5 ± 1.0%, 12.1 ± 1.3%, and 6.6 ± 2.3% for ET, HIT, and SIT, respectively.

After adjustment for covariates (training frequency, training intervention weeks, initial fitness level, disease status, sex, and age), all training intensity categories were associated with increases in $\dot{V}$O_2_max (ET, 10.2 ± 1.0%; HIT, 12.0 ± 1.3%; SIT, 8.9 ± 2.6%; all *P* < 0.001; model 14). There was a tendency for both ET and SIT to have lower responses compared with HIT (both *P* = 0.082). Of note, these estimated marginal means comparing the training intensity categories are slightly different from the mean of the training groups’ individual changes presented previously due to weighting, log-transformation before modeling, and adjustments for covariates, especially since SIT studies on average were associated with lower number of training intervention weeks and lower training frequency (see Table 1). The increase in mito_pooled_ after training was 2.2, 2.2, and 3.0 times larger than the increase in $\dot{V}$O_2_max for ET, HIT, and SIT, respectively (the estimated marginal means for mito_pooled_ divided by those for $\dot{V}$O_2_max).

When studying the time-course of $\dot{V}$O_2_max changes, all training intensity categories increased $\dot{V}$O_2_max after only 2 weeks of training (ET, 3.6 ± 2.1%; HIT, 6.9 ± 3.1%; SIT, 7.9 ± 3.4%; all *P* < 0.001; Fig. 7B; model 14). The increase in $\dot{V}$O_2_max by intervention weeks followed log-linear relationships, with ET and HIT showing significant increases between 2–6 weeks and 6–10 weeks of training (all, *P* < 0.01). However, this increase was not significant for SIT (*P* = 1.00; Fig. 7B). Training frequency had a log-linear impact on $\dot{V}$O_2_max, with six sessions/week being more potent than four and four sessions/week being more potent than two (both *P* = 0.0012; Fig. 7C; model 14). $\dot{V}$O_2_max increased with exercise training irrespective of initial fitness level, including in well-trained participants (4.5 ± 3.1%; *P* = 0.004; Fig. 7D). However, $\dot{V}$O_2_max tended to increase to a greater extent in previously moderately trained participants compared to well-trained participants (mean difference: Δ 3.7 ± 4.1% points; *P* = 0.027; Fig. 7D), and significantly more in previously untrained participants compared with moderately- (Δ 3.7 ± 2.4% points; *P* < 0.001; Fig. 7D) and well-trained participants (Δ 7.6 ± 4.2% points; *P* < 0.001; Fig. 7D; model 14). The exercise training response was not affected by disease status (*P* = 0.263; Fig. 7E; model 14) but there was a tendency for young individuals (< 35 years) to increase $\dot{V}$O_2_max more than those > 35 years (*P* = 0.067; Fig. 7G; model 14). Additionally, for the analyses of the absolute change in $\dot{V}$O_2_max (Δ mL/kg/min), participants aged < 35 years (4.8 ± 0.4 mL/kg/min) were found to increase $\dot{V}$O_2_max more than those aged 35–55 (2.9 ± 0.6 mL/kg/min; *P* < 0.001) and > 55 years (2.2 ± 0.6 mL/kg/min; *P* < 0.001; Supplementary Information 3, Fig. S6G). Furthermore, women increased $\dot{V}$O_2_max more than men when expressed in percentage change terms (men versus women: Δ 3.0 ± 2.2% points; *P* = 0.008; Fig. 7F) but the effects were similar in absolute change terms (3.8 ± 0.8 versus 3.7 ± 0.4 mL/kg/min in women and men, respectively; *P* = 0.976; Supplementary Information 3, Fig. S6F). When split into disease groups, neither young participants (< 35 years) with metabolic diseases (*P* = 0.443) nor old participants (> 55 years) with metabolic diseases (*P* = 1:00), CVD (*P* = 1.00), or COPD (*P* = 0.333) responded differently to exercise training compared to healthy, age-matched, and initial fitness level-matched (i.e., only untrained) participants, when training intensity, frequency and intervention weeks were controlled for (Table 3; model 15). No differences between disease groups and healthy individuals were found when analyzed as Δ mL/kg/min (*P* ≥ 0.099; Supplementary Information 3, Fig. S7A). Young, healthy, untrained women increased $\dot{V}$O_2_max more than young men expressed in percentage change terms (*P* = 0.004; Table 3; model 16) but similarly in mL/kg/min (*P* = 0.382; Supplementary Information 3, Fig. S7B) when controlling for training intensity, frequency and intervention weeks. This difference in percentage change between sexes vanished in the older (> 55 years) age group (*P* = 0.506) since old women tended to have a lower percentage increase in $\dot{V}$O_2_max than their younger counterparts (*P* = 0.060; Table 3). No difference between young and old men was observed in percentage changes (*P* = 0.450) but the older group increased $\dot{V}$O_2_max less in absolute values (3.2 ± 0.8 versus 5.6 ± 0.5 mL/kg/min; *P* < 0.001; Supplementary Information 3, Fig. S7B).

##### *Change in Maximal Oxygen Consumption Per Hour of Exercise Training (Training Efficiency)*

**Fig. 8:**
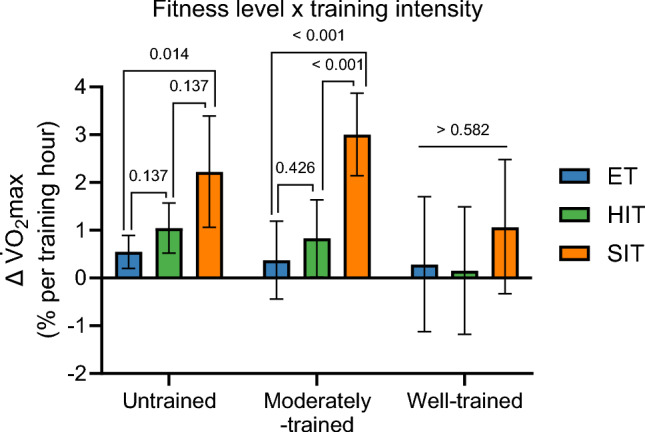
The interaction effect of initial fitness level and training intensity category on changes (Δ) in $\dot{V}$O_2_max per training hour (Fig. 8; model 17). Values are estimated marginal means with 95% confidence limits. (*N* studies = 202, *N* training groups = 295, *N* participants = 3487)

When normalizing the percentage change in $\dot{V}$O_2_max to total hours of exercise training (Fig. 8; model 17), SIT was ~ 2.9 times more efficient than HIT and ~ 4.9 times more efficient than ET (both *P* < 0.001), while HIT was ~ 1.7 times more efficient than ET, although this difference did not reach statistical significance (*P* = 0.166). This trend of progressively greater efficiency SIT > HIT > ET was consistent across different initial fitness levels (Fig. 8; model 17). Of note, well-trained individuals did not show any change in $\dot{V}$O_2_max when expressed per hour of exercise training for any training intensity category (*P* > 0.136; Fig. 8).

#### Correlations

**Fig. 9:**
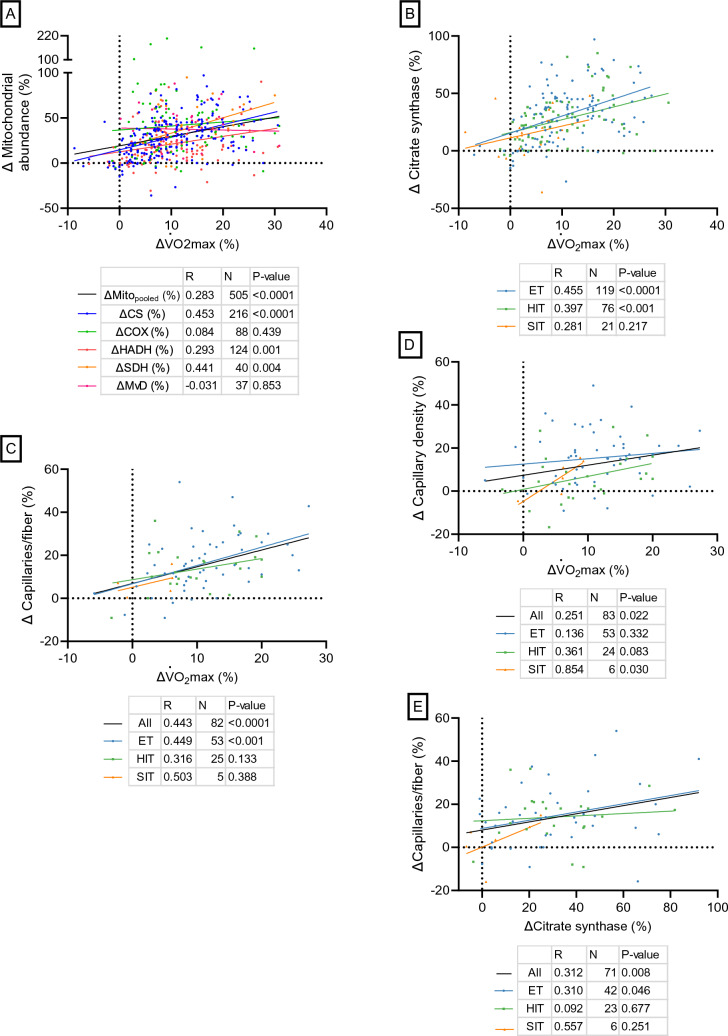
Correlation plots between relative changes (Δ) after exercise training in maximal oxygen consumption ($\dot{V}$O_2_max) and muscle mitochondrial (**A**, **B**) and capillary measures (**C**, **D**). Only training interventions using exercises recruiting large muscle mass were used. **E** shows correlation plot between relative changes (Δ) in citrate synthase content and capillaries per muscle fiber after exercise training (both exercises recruiting small and large amounts of muscle mass were used). COX, cytochrome c oxidase/complex IV; CS, citrate synthase; ET, endurance training; HADH, hydroxyacyl-CoA dehydrogenase; HIT, high-intensity interval training; MvD, mitochondrial volume density; SDH, succinate dehydrogenase/complex II; SIT, sprint interval training

The relative changes in $\dot{V}$O_2_max and mito_pooled_ in response to exercise training were significantly correlated (*r* = 0.283, *P* < 0.0001, 505 observations; Fig. 9A). Specifically for the singular mitochondrial content markers, the relative change in $\dot{V}$O_2_max was significantly correlated with the changes in CS (*r* = 0.453, *P* < 0.0001, 216 observations; Fig. 9A), HADH (*r* = 0.293, *P* = 0.001, 124 observations; Fig. 9A), and SDH (*r* = 0.441, *P* = 0.004, 40 observations; Fig. 9A) but not with COX (*r* = 0.084, *P* = 0.439, 88 observations; Fig. 9A) or MvD (*r* =  − 0.031 *P* = 0.853, 37 observations; Fig. 9A). Regarding the relative changes in $\dot{V}$O_2_max and CS, both ET (*r* = 0.455, *P* < 0.0001, 119 observations; Fig. 9B) and HIT (*r* = 0.397, *P* < 0.001, 76 observations; Fig. 9B) showed significant correlations, while SIT (*r* = 0.281, *P* = 0.281, 21 observations; Fig. 9B) did not.

The relative changes in $\dot{V}$O_2_max and the capillary markers C/F and CD were also significantly correlated (*r* = 0.443, *P* < 0.0001, 82 observations and *r* = 0.251, *P* = 0.022, 83 observations, respectively; Fig. 9C, D, respectively). However, for change in C/F, only ET showed significant correlation with the change in $\dot{V}$O_2_max (*r* = 0.449, *P* < 0.001, 53 observations; Fig. 9C), while only SIT displayed a significant correlation between CD and $\dot{V}$O_2_max (*r* = 0.854, *P* = 0.030, 6 observations; Fig. 9D).

There was a significant correlation between the relative changes in C/F and CS (*r* = 0.312, *P* = 0.008, 71 observations; Fig. 9E).

## Discussion

This systematic review and meta-regression covered 50 years of data from 5973 participants and investigated the impact of exercise training variables on skeletal muscle mitochondrial biogenesis and capillarization. Our main findings were as follows: (1) larger training volumes (higher training frequency per week and larger number of training intervention weeks) and higher training intensities (per hour of training, SIT > HIT > ET) are associated with greater increases in mitochondrial content and $\dot{V}$O_2_max. (2) SIT is effective in improving mitochondrial content and $\dot{V}$O_2_max in the early stages of exercise training. In contrast, ET and HIT show slower initial increases but continue to improve steadily over a greater number of training weeks. (3) Gains in capillarization occur primarily in the early stages of training (< 4 weeks) and are observed only in untrained to moderately trained participants. Capillaries per fiber (CF) increase similarly with ET, HIT, and SIT, while capillaries per mm^2^ fiber area (CD) increase only after ET and HIT, with ET showing larger increases compared to HIT and SIT. (4) Generally, responses to exercise training are largely determined by the initial fitness level and are not influenced by age, sex, disease, and menopause. However, women display larger percentage gains in *V*O_2_max compared with men.

In the past five years, a debate coined “the mitochondrial content contest” by Fiorenza and Lemminger [39] has emerged in the scientific community focusing on whether exercise training intensity or volume is the most important exercise training variable for promoting increases in mitochondrial content [8, 10]. Granata and colleagues [11] pooled the results from 58 exercise training studies and found no significant association between exercise training intensity (% of Wmax) and change in mitochondrial content, as assessed by either CS activity or MvD. Instead, they highlighted a positive relationship between total exercise training volume, which they defined as the exercise intensity multiplied by the total training duration (which in the current study is defined as training load, see explanation below), and changes in mitochondrial content. This led them to advocate for exercise training volume as the primary driver for increasing mitochondrial content [10, 11]. In response, MacInnis et al. [8] contend that exercise training intensity is the most important variable mediating exercise-induced increases in mitochondrial content within a fixed training duration. In support of their view, they emphasize that (1) findings from acute exercise studies where mRNA expression of peroxisome proliferator activated receptor ƴ *c*oactivator 1α (PGC-1α), a key regulator of mitochondrial biogenesis, increases to a larger extent following HIT than work-matched low-intensity exercise [40], and (2), findings from longitudinal studies comparing changes in mitochondrial content show larger increments in mitochondrial content after HIT compared with work-matched, moderate-intensity continuous exercise training (e.g., [41]). Furthermore, they point out studies comparing low-volume SIT with higher volume of moderate-intensity continuous exercise, where similar mitochondrial adaptations were observed (e.g., [42]). In the present study, we adopted a slightly different and more nuanced statistical approach compared with previous research. Our aim was to investigate the distinct contributions of each factor constituting the training load: training intensity, training frequency per week, and the number of training intervention weeks. In each analysis, we controlled for the other factors when investigating the factor of interest. Additionally, we conducted analyses to assess training responses per hour of training across the three training intensity categories, aiming to investigate training efficiency. Our findings provide evidence that supports both sides of the debate, underscoring the importance of both training intensity and volume. Specifically, we observed that ET, SIT, and HIT did not promote differential changes in mitochondrial content after adjustment for the total number of training sessions. However, the increase in mitochondrial content per hour of exercise training followed an exercise intensity-dependent pattern (SIT > HIT > ET). This highlights the role of exercise training intensity in enhancing mitochondrial content. Furthermore, our study also shows greater elevations of mitochondrial content with higher training frequency per w*e*ek and, for ET and HIT, greater number of training intervention weeks, supporting the argument for training volume. Notably, we observed that high-intensity training, and especially SIT, may reach a plateau in mitochondrial adaptations sooner than lower intensity training. Our understanding of the accumulated evidence is therefore simple; a high exercise intensity will compensate for a low training volume in exercise training interventions with a short duration, but a larger exercise training volume is necessary to promote further increments in mitochondrial content over time, as indicated by SIT not leading to a further increase in mitochondrial content after the initial 2 weeks of exercise training. However, for a given exercise training volume (i.e., number of exercise training hours), training with a high exercise intensity is the most efficient way of increasing mitochondrial content, as SIT in this context showed larger increments than both HIT and ET, and HIT was more efficient than ET.

The *Journal of Physiology* crosstalk about the mitochondrial content contest [8, 10, 39] was probably influenced by semantic differences among researchers in how to interpret the exercise training volume and intensity terms. In the current study we have, consistently with other researchers [12–14], defined exercise training volume as the total exercise training time (i.e., minutes per training session × training session frequency). However, other researchers, such as Granata and colleagues [11], interpret this term differently. As mentioned, they define exercise training volume as the exercise intensity multiplied by the total training duration. This interpretation is the same as Banister and colleagues [12] in 1975 defined as exercise training load, a term which has been widely used with the same interpretation in later years [13–16]. However, in the recent crosstalk debate, the semantic issues of distinguishing between exercise training volume and -load were not properly addressed. Albeit belatedly, we consider it reasonable to put forward the argument that two exercise training variables (i.e., exercise training volume and intensity) against one (i.e., exercise training intensity) is an unfair match for evaluating the most important variable mediating exercise-induced increases in mitochondrial content. The term training load may be used to describe the physiological stress a certain amount of exercise training poses on an individual, which, as expected, leads to a given magnitude of adaptation. However, the training load for an individual is largely affected by their fitness level. For example, 1 h of ET will pose a larger physiological stress on an untrained individual than a well-trained individual, and typically lead to greater training adaptations. In the present study, to elucidate this discrepancy in exercise training-induced adaptations in response to training volume and intensity between individuals with different fitness levels, we estimated the percentage improvement per hour of ET, HIT, and SIT for the variables mitochondrial content and $\dot{V}$O_2_max (Figs. 4, 8, respectively). When planning training, such an approach can be used as a training load factor for predicting adaptations in relevant physiological variables to a given training stimulus.

Exercise training-induced skeletal muscle capillary growth appears to be influenced by at least two physiological factors: (1) increased blood flow and thereby shear stress on endothelial cells [43], and (2) mechanical stretch of the tissue [44]. It is also likely that factors related to increased muscle metabolism and/or hypoxia with exercise add an additional angiogenic stimulus [45]. In the present study, we show that C/F is increased to a similar extent by ET, HIT, and SIT when number of training intervention weeks and initial fitness level of the participants are controlled for. This finding contrasts with the prevailing view that the most potent type of exercise for inducing capillary growth is long-duration training sessions conducted at a low- to moderate exercise intensity [24]. This perspective is based on the finding of attenuated VEGF levels after HIT compared with low-intensity exercise [27]. Nor is it in line with the indications of more pronounced capillary adaptations after SIT compared with HIT in a recent meta-analysis [29], although that finding, in particular, should be interpreted with caution owing to the low number of studies included in the analysis (two SIT studies and one HIT study [29]). However, ET in the current study did indeed lead to a greater increase in CD compared to both HIT and SIT, which may be used as further evidence for low- to moderate intensity training sessions to be the superior type of exercise in promoting muscle capillary growth. Importantly, the present finding of superior change in CD with ET was not due to greater capillary growth per se, but rather a consequence of numerically lower muscle hypertrophy response (i.e., change in muscle fiber CSA) compared with HIT and SIT. We cannot rule out the possibility that methodological decisions may have influenced the capillarization results, such as adjusting for covariates, instead of only displaying the mean changes as in Fig. 5J. Moreover, the ET exercise intensity category in the current study must be considered as quite heterogeneous, spanning all types of exercise activities and a wide range of minutes per training session conducted in a continuous fashion with an exercise intensity below the second ventilatory threshold/4 mmol/L blood lactate concentration/87% of HRmax/87% of $\dot{V}$O_2_max /75% of Wmax. The heterogeneity of training interventions within the different exercise intensity categories may thus have diluted the exercise intensity-dependent capillarization effects. More research is therefore needed to further elucidate the etiology of exercise-induced capillary growth. Common for both C/F and CD was that the increase occurred in the early stages of exercise training onset (i.e., ≤ 4 weeks), with greater number of training intervention weeks not associated with further improvements. This is strikingly similar to the time course of exercise training-induced angiogenesis that has been demonstrated in rodent skeletal muscle [46]. Additionally, this agrees with the finding of Jensen et al. [47] who sampled multiple muscle biopsies across a seven-week training intervention but observed no further increase in capillarization beyond 4 weeks of exercise training. This phenomenon is speculated to be owing to the fact that the initial exercise training-induced increase in vascularization possibly reduces the shear stress on the endothelial cells, and thus provides a weaker angiogenic stimulus. Longer-duration training interventions are therefore requested to fully uncover the evolution of vascularization in human skeletal muscle and to explain the major differences in mean baseline C/F values between untrained, moderately trained, and well-trained participants (1.7, 2.6, and 3.0, respectively) as described in the present analysis (Table 2).

It is widely reported that $\dot{V}$O_2_max gradually decreases with advancing age [22, 48, 49] owing to associated reductions in central factors (e.g., decreases in blood volume, maximal heart rate, and stroke volume) as well as peripheral factors (reduced muscle capillarization, mitochondrial content and oxidative capacity) [50]. However, exercise training has previously been reported to improve $\dot{V}$O_2_max by a similar magnitude across age-groups [51, 52], which generally is in line with the percentage change findings of the present study. Across age groups, the percentage improvements in $\dot{V}$O_2_max, mitochondrial content and capillarization were not different, indicating that trainability is largely maintained throughout the lifespan although participants < 35 years tended to display larger percentage $\dot{V}$O_2_max gains than their older counterparts. However, in terms of the absolute change of body weight-normalized $\dot{V}$O_2_max, participants < 35 years demonstrated larger increases than participants between 35–55 years and > 55 years, which as probably partially related to the lower baseline values in these age groups. Furthermore, absolute values of $\dot{V}$O_2_max are known to be closely related to factors, such as total lean body mass [53, 54], and cardiac size [55], both of which are known to decrease with increasing age [56, 57]. In theory, such relationships between dimensions of different body parts and $\dot{V}$O_2_max also suggest that the absolute changes in $\dot{V}$O_2_max to exercise training are scaled to the absolute proportions of factors such as lean body mass and cardiac size, thus giving persons with lower initial levels of these characteristics, as older individuals, lower absolute changes in $\dot{V}$O_2_max. Moreover, increases in $\dot{V}$O_2_max were not significantly different following ET, HIT, and SIT. This is in agreement with previous meta-analyses showing no difference for changes in $\dot{V}$O_2_max with HIT compared with SIT [58, 59] and studies displaying no difference in effect between SIT and ET [60, 61]. However, it is contrary to the previous finding of HIT to be more potent than ET in improving $\dot{V}$O_2_max [62], although there was a tendency toward greater $\dot{V}$O_2_max increases with HIT compared with ET in the present study. The discrepancies across studies could potentially be partly explained by different inclusion criteria, i.e., HIT criteria being 90–95% of HRmax in the aforementioned meta-analysis [62]). Interestingly, we found that women in general have larger percentage increase in $\dot{V}$O_2_max than men in response to exercise training, while displaying similar increases to males expressed in mL/kg/min. This is, to a certain extent, in contrast to what is previously observed for cardiac adaptations, where males seem to improve more than females [63], and in contrast to a recent meta-analysis finding a 2 mL/kg/min larger increase in $\dot{V}$O_2_max after exercise training in males than females [64]. However, it must be emphasized that the mentioned meta-analysis only included eight studies (eight training groups of men and eight training groups of women), while model 17 in the present study included 24 and 183 training groups of women and men, respectively. Moreover, since men in general have a higher baseline $\dot{V}$O_2_max, it can be calculated that the mean percentage increase in body mass-normalized $\dot{V}$O_2_max was exactly 16% for both men and women for the eight included studies in Diaz-Canestro and Montero [64]. Furthermore, the above analysis only included studies in which both male and female participants were recruited in the same study and completed the same training program, but failed to include data from a few other studies with these criteria, such as Hoppeler et al. [65] (10% and 19% increase in $\dot{V}$O_2_max for men and women, respectively) and one of the largest exercise training intervention studies conducted on both sexes, the HERITAGE study [52]. That study included 633 participants (287 men and 346 women) who carried out 20 weeks of ET, with men and women increasing $\dot{V}$O_2_max by 5.5 and 5.2 mL/kg/min, respectively, and the percentage increase being significantly lower for men (15.9% and 19.5% for men and women, respectively; *P* < 0.01). This sex difference persisted even when normalizing $\dot{V}$O_2_max to fat-free mass instead of body mass (14.6% and 17.9% increase for men and women, respectively; comparison between sexes: *P* < 0.01) [52], which, in our opinion, is the fairest comparison between sexes both for baseline and trainability comparisons owing to initial differences in body size and composition. Furthermore, a recent meta-analysis also concluded that there are no clear indications of sex-specific differences in $\dot{V}$O_2_max trainability [66]. Hence, we argue that the summative information indicates similar or even better $\dot{V}$O_2_max trainability for (premenopausal) women than men.

Recently, the magnitude of both central and peripheral (i.e., vascular) training adaptations has been reported to differ in pre- and recently post-menopausal women (< 5 years) compared with women initiating exercise training later after the menopause [22]. This finding is thought to be related to the estrogen status and changes in estrogen receptor signaling [67]. In support of this, the present data show indications (*P* = 0.060) of greater percentage increases in $\dot{V}$O_2_max in young women compared with older women above 55 years of age (significantly larger in mL/kg/min). However, these analyses must be interpreted with caution since only six and five training groups of untrained, healthy young and old women were included in this dataset, respectively. Moreover, exercise training-induced changes in mitochondrial content and capillarization are still very similar between sexes and age groups, and remain potent if the exercise intensity is high, at least when comparing untrained young (< 35 years) and old (> 55 years) women and men. This suggests, despite an age-related decrease in physical performance, that regular exercise training can oppose this decrement, that mitochondrial and $\dot{V}$O_2_max trainability is largely maintained throughout life and highly affected by the total training load (training intensity × volume), and that trainability is primarily determined by the initial fitness level. Interestingly, our data did not show any conditional effect of disease status. This is somewhat contrary to the view that common comorbidities with a range of diseases, such as systemic inflammation, insulin resistance, low capillarization, and mitochondrial content and function, in addition to the use of certain medications, are associated with impaired responses to exercise training [68–72]. This finding was further strengthened in our post hoc analyses which sub-analyzed different disease groups: in terms of percentage changes in mitochondrial content, capillarization or $\dot{V}$O_2_max, people with metabolic diseases, CVD, or COPD did not respond differently to exercise training compared with healthy, age-matched, and initial fitness level category-matched participants (i.e., untrained < 45 mL/kg/min). This may, therefore, be related to initial lower levels of fitness amongst different disease groups, since we did not differentiate by specific $\dot{V}$O_2_max, and thus may have a greater potential for adaptations, as previously discussed [72]. Importantly, this also reiterates the beneficial effects of exercise training across all groups in the population. The finding is very consistent with the common understanding of magnitude of training-induced adaptations being inversely proportional to the initial fitness level [51, 73, 74], regardless of sex and age. Of note, in terms of absolute changes in C/F, this variable was found to increase less with exercise training in young individuals with metabolic diseases and older individuals with COPD compared to their age-matched healthy controls. This differentiates from the percentage change results, implying that lower baseline values in these groups had an impact. COPD subjects are clearly limited during exercise by their low cardiopulmonary capacity, particularly when conducting whole-body exercises [75, 76]. Feasibly, the lower blood flow to exercising muscles in COPD participants may have provided a weaker angiogenic stimulus for participants with COPD compared with the healthy participants.

In this study we also show that exercising with a small amount of muscle mass (such as during one-legged cycling or knee extension exercises), which provides a higher capacity for muscle mass-specific energy turnover compared with whole-body exercises owing to less oxygen-delivery (central) limitations [75, 77], does not translate into greater increases in mitochondrial content in the stimulated muscle. This suggests (1) that the degree of local muscle metabolic perturbations (i.e., accumulation of metabolites such as hydrogen ions and inorganic phosphate) during exercise is the primary stimulus for mitochondrial growth, and (2) that the mass-specific energy turnover in the muscle is not predictive of mitochondrial adaptations, and hence, not a suitable measure of training load. The association between central and peripheral adaptations does not occur in a 1:1 ratio. For instance, one-legged endurance training is associated with minor stress on the cardiovascular system and thus has little potency for central cardiovascular adaptations in healthy individuals, as evident by whole-body $\dot{V}$O_2_max being unchanged after one-legged knee extension endurance training [78] and only small changes occurring after one-legged cycling endurance training [79, 80]. Moreover, since small amounts of muscle mass are stimulated during isolated training models, it must be emphasized that the favorable mitochondrial adaptations only take place in the stimulated muscle and lead to less expansion of the total body’s “mitochondrial pool” than for whole-body exercises [81].

In the current study, the ratio of change in mitochondrial content compared to change in $\dot{V}$O_2_max was larger with SIT compared with ET and HIT (3.0, 2.2, and 2.2, respectively), which could indicate that the balance between central to peripheral adaptations is lowest for SIT. Indicatively, Vigelsø et al. [82] observed a moderate correlation of *r* = 0.42 between change in CS activity and change in $\dot{V}$O_2_max for ET interventions, which is consistent with our observation (*r* = 0.46). Surprisingly, the change in $\dot{V}$O_2_max was not associated with the change in MvD, opposing the seemingly accepted dogma of a near and direct relationship between $\dot{V}$O_2_max and MvD [65, 83, 84]. The current study also demonstrates a positive correlation between mitochondrial (CS content) and capillary (C/F) adaptations to exercise training (Fig. 9E). The cooccurrence of these peripheral adaptations may partly be explained by shared signaling pathways. For instance, PGC-1α, a major regulator of mitochondrial biogenesis in response to exercise training, can also regulate VEGF expression and angiogenesis [85]. Taken together, mitochondrial growth seems primarily to be determined by local muscle metabolic perturbations during exercise, and the ratio between training-induced change in $\dot{V}$O_2_max and mitochondrial content are different between exercises engaging small amounts of muscle mass and whole-body exercises, as well as between exercise intensity categories. Exercise training-induced increases in mitochondria and capillarization seem to be modestly correlated.

As a secondary aim of this study, we compared the effects of exercise training on muscle fiber type I proportion in studies that were already included in the qualitative synthesis of this review. This was done with the aim of elucidating a debated topic using a rather high number of exercise training studies: whether the proportion of the more oxidative and fatigue-resistant muscle fiber type I may be influenced by exercise training. Although numerous studies have clearly demonstrated that prolonged exercise training promotes transformation within the fast-twitch fiber types (from type IIx to type IIa; e.g., [86, 87]), it remains to be experimentally confirmed if exercise training-induced transformation from muscle fiber type II to type I is occurring [88]. After pooling all exercise training studies in the current study, no such main effect was displayed. However, the pre- to post-test change in fiber type I proportion was different between ET and SIT, suggesting transformative effect towards an aerobic, type I, muscle phenotype with ET, and towards an anaerobic-glycolytic, type II, muscle phenotype with SIT. This indicates that fiber type transformation between type I and II can occur with exercise training. Moreover, case studies have described that increases in fiber type I proportion can occur at the expense of type II fibers [89, 90]. Such a transformation likely requires years of deliberate exercise training [89, 90], and has, therefore, been difficult to detect after relatively short training durations (i.e., weeks or months of exercise training) usually applied in exercise training studies. A notion to support our finding is the fact that well-trained endurance athletes generally express a greater proportion of type I fibers compared to their untrained counterparts [91], which is confirmed by the present data where well-trained displayed significantly higher pre-training type I proportion (mean ± 95% CI: 54.8 ± 6.4) than untrained participants (45.2 ± 2.2; *P* = 0.018), which also tended to be greater than for moderately-trained participants (49.4 ± 3.8; *P* = 0.116).

### Methodological Considerations

Physiological responses to exercise training are not uniform between individuals and covary with individual characteristics, such as genetics, epigenetics, and composites of the inner physiological milieu. Moreover, exercise training studies are associated with some inherent limitations that may affect methodological quality and thus the interpretation of the data. For any research project that aims to investigate the etiology of responses to exercise training, the interpretation of their outcome data is thus a complicated task, which is also the case for this systematic review. First, for exercise training studies in general, as well as for the studies included in the qualitative synthesis of this review, there is a lack of adherence to the general principles of exercise training. Compliance with the stringent research principle of study protocol standardization can often be at the expense of particularly the training principles of individualization and progression*.* This, in turn, may be a threat to the ecological validity of the findings, as such principles commonly are taken into consideration when preparing a training program in “actual settings”. Moreover, exercise training studies are usually limited to a relatively short training duration (i.e., weeks or months of exercise training) owing to resource limitations affecting both study participants and staff, which may imply that the findings in the present study most of all are generalizable to initial responses to an altered exercise training stimulus. Second, exercise training studies investigating the muscle physiological responses to exercise training are to a variable extent riddled with sources of error associated with the muscle biopsy technique. Muscle fiber characteristics are not uniform across the whole muscle [92], and assessments derived from muscle samples are thus vulnerable to reproducibility issues since repeated muscle sampling for a study participant are sampled at slightly different locations. Such issues may be further complicated if artifacts in the specimens, such as the amount of blood and connective tissue, are not taken into consideration when preparing the muscle biopsy [93]. Assessments of muscle characteristics may also be prone to analytical issues which in turn can influence the validity of the respective studies. The research quality is thus dependent upon the fact that each laboratory has developed reliable and solid, documented methods [93]. Unfortunately, for the research articles included in the present study, the laboratories’ coefficient of variation for the relevant methods was rarely stated. Third, the accuracy of meta-regression analyses depends heavily on the quality of the included studies and the data extraction. In the current study, we implemented several preventive measures to enhance data quality, particularly through a careful and rigorous screening procedure, as detailed in Sect. 2.3. Data extraction. However, the potential impact of publication bias has not been specifically addressed. Our comprehensive approach, which included broad inclusion criteria and extensive metadata extraction for each study, did not incorporate statistical methods to evaluate publication bias. Therefore, the potential impact of publication bias remains a limitation of this study. As the mitochondrial perspective of this study was limited to elucidate which variables influence the exercise training-induced increase in mitochondrial content, it was not in the scope of this review to investigate other potential factors affecting exercise training-induced changes in different mitochondrial functions. This study is thus not able to provide conclusions regarding mitochondrial qualitative changes with exercise training such as levels of fat oxidation and global respiratory capacity per muscle weight. Moreover, owing to differences in methodology between laboratories, between dry/wet weight muscle, normalization strategies used, and the wide range of metrics and absolute levels of for example CS activity between studies, even when recalculated into the same metric (e.g., µmol/min/g protein), made it impossible to conduct meta-regression on the absolute changes and compare those between population groups. Therefore, the mitochondrial data presented herein can only be used for investigating relative (%) changes with training. Nor are the data suitable for differentiating between the two populations of mitochondria inside the muscle cells, i.e., subsarcolemmal and intermyofibrillar mitochondria. Subsarcolemmal mitochondria work primarily to sustain the bioenergetic demands of active membrane ion and substrate transporters, while intermyofibrillar mitochondria are primarily responsible for the bioenergetics needed to enable muscle contractions [94]. These two mitochondrial populations are shown to possess divergent responses to exercise training: in relative values (%), the exercise training-induced change in mitochondria appear to be larger in subsarcolemmal than intermyofibrillar mitochondria [65, 95]. However, since the majority of mitochondria are located in between myofibrillar sarcomeres [94], the absolute changes tend to actually be greatest in the intermyofibrillar population [65, 95]. Unfortunately, too few studies have distinguished between subsarcolemmal and intermyofibrillar changes in mitochondrial content to analyze if the exercise training-induced changes in the two different mitochondrial populations rely upon different factors and training stimuli.

Furthermore, it is important to note that a change in one mitochondrial structural or functional variable does not necessarily imply that other mitochondrial variables are altered to the same degree [94, 96], which was the case for HADH in the present study. To increase statistical power in the mitochondrial content analyses, and to reduce the risk of type II errors, we chose to pool the assessments of different mitochondrial content markers and to use this pooled measure (i.e., mito_pooled_) as the most meaningful stand-alone interpretation in analyses. In support of this action, we show that the different mitochondrial content markers used in mito_pooled_ display the same responses to exercise training (Fig. 2A) and that the same analyses conducted on CS alone, the most utilized marker of mitochondrial content, show highly similar findings (Supplementary Information 2). The exception to this was the HADH response to exercise training, which was slightly lower than for the other mitochondrial markers. However, enzyme markers of beta-oxidation generally display numerically lower responses to exercise training than enzyme markers involved in the citric acid cycle and the electron transport chain (e.g., [78, 97–104]), potentially owing to the observation that most studies included in this review conducted their exercise training in a fed state, which is shown to inhibit fat oxidation during exercise compared to exercising in a fasted state, resulting in lower HADH activity training responses [105]. Moreover, beta-oxidation of fatty acids is an essential attribute of the mitochondria, and we therefore considered it necessary to include HADH in such a pooled mitochondrial measure on the same level as enzyme markers from the citric acid cycle and the electron transport chain. To account for this in the model, the type of mitochondrial marker was added as a separate random effect.

In this context, it is also timely to mention that the capillary measures investigated in this study have limitations, and their functional relevance has to some extent been questioned. Both CD and C/F are derived from two-dimensional images of transverse muscle sections. However, capillaries form a three-dimensional network surrounding muscle fibers where the direction of the capillaries often can deviate substantially from the direction of the longitudinal axis [106, 107]. Thus, CD and C/F do not take into account capillary tortuosity and may not accurately reflect muscle capillarization or blood perfusion of the muscle fibers. Additionally, current research assumes that factors other than the quantity of capillaries are of importance for the blood perfusion of the muscle. For instance, both the positioning of the capillaries [108, 109], and the thickness of the capillary basement membranes [110, 111] can influence the diffusion conditions for gas and metabolic substrate/by-product exchange between capillaries and muscle fibers. Methods to study such factors are emerging [107], and these will hopefully in the future provide more insight about how to induce functional capillary adaptations with exercise training.

To further elucidate the role of aging, sex, and disease on exercise training-induced adaptations, subanalyses were performed in the present study. Within the limitations of this meta-regression, women do not seem to respond differently to exercise training than men regarding mitochondrial and capillary changes but do for $\dot{V}$O_2_max (women increase $\dot{V}$O_2_max similarly to men in mL/kg/min, but due to their lower baseline level, their percentage increase is higher). This conclusion is limited by the fact that very few of the included studies have thoroughly controlled for differences in the hormonal cycle of the female participants, but the general lack of difference in adaptations between sexes indicates that this has a minor effect on training adaptations. Furthermore, we conducted sub-divisional analyses of females performing training interventions before and after menopause based on the assumption that no females < 35 years were menopausal, and that all females > 55 years were. As highlighted by Tamariz-Ellemann and colleagues, the trainability of females seems to change within a few years after the menopause [22]. In our analysis, a tendency to lower percentage change in females > 55 years compared with those < 35 years was displayed. Our analysis also showed that $\dot{V}$O_2_max reported in mL/kg/min increased less in older (> 55 years) compared with young (< 35 years) women, but importantly, this reduction in absolute terms $\dot{V}$O_2_max change to exercise training from > 35 to < 55 years was also displayed for males. The lack of information regarding onset of menopause of the females included in this meta-regression may have limited our ability to confirm the notion of reduced trainability of females after menopause, and underlines the necessity of investigating sex-specific alterations in future controlled studies. Our sub-group disease analyses of participants with a known metabolic, cardiovascular, or chronic obstructive pulmonary disease, may also be riddled with similar methodological issues. Each category spans a wide range of conditions which may have different impacts on training adaptations.

## Conclusions

In this systematic review and meta-regression covering ~ 50 years of research data, we demonstrate that the magnitude of change in mitochondrial content, capillarization, and $\dot{V}$O_2_max to exercise training is largely determined by the initial fitness level. The ability to adapt to exercise training is maintained throughout life irrespective of sex and presence of disease. Larger training volumes (higher training frequency per week and larger number of training weeks) and higher training intensities (per hour of training, SIT > HIT > ET) are associated with greater increases in mitochondrial content and $\dot{V}$O_2_max. Therefore, training load (volume *x* intensity) is a robust predictor of changes in mitochondrial content and $\dot{V}$O_2_max. Increases in capillarization occur primarily in the early stages of exercise training (< 4 weeks) with ET, HIT, and SIT equally enhancing capillaries per fiber, while ET is more effective in increasing capillary density (capillaries per mm^2^) due to less pronounced muscle fiber hypertrophy.

## Supplementary Information

Below is the link to the electronic supplementary material.

Supplementary file1 Supplementary Information 1: Summary of statistical models (DOCX 19 KB)

Supplementary file2 Supplementary Information 2: Analysis of citrate synthase (DOCX 197 KB)

Supplementary file3 Supplementary Information 3: Analysis of absolute changes in capillary markers and VO2max (DOCX 405 KB)

Supplementary file4 Supplementary Information 4: Reference list of research articles included in mitochondrial analysis (PDF 248 KB)

Supplementary file5 Supplementary Information 5: Reference list of research articles included in capillary analysis (PDF 159 KB)

## Declarations

### Availability of data and material

The dataset, statistical analysis codes, and SAS scripts are available from the study’s GitHub repository. To request access, please contact the corresponding author with your GitHub username, and access will be granted upon reasonable request.
