# Supplementary material for: Effects of Exercise Training on Mitochondrial and Capillary Growth in Human Skeletal Muscle: A Systematic Review and Meta-Regression
Source: Sports Med. 2024 Oct 10;55(1):115–44. doi: 10.1007/s40279-024-02120-2 (PMC11787188; doi:10.1007/s40279-024-02120-2)
Supplement: Supplementary file 1 — Supplementary file1 Supplementary Information 1: Summary of statistical models (DOCX 19 KB) [file 40279_2024_2120_MOESM1_ESM.docx]

| Model nr. | Dependent variable (DP) | DP form | Fixed effects | Random effects | Data used | Fig. nr./table |
| --- | --- | --- | --- | --- | --- | --- |
| 1 | Mitochondrial abundance markers | Fold-change, log-transformed | Mitochondrial marker (five levels: MvD, CS, SDH, COX and HADH) | Study ID, training group ID, and allowing for residual variance | All data  N studies: 353  N training groups: 506  N participants: 5650  N observations: 943 | 2 |
| 2 | Mito_pooled_ | Percent change | Training intensity category (three levels, SIT, HIT and ET) | None | All data  N studies: 353  N training groups: 506  N participants: 5650  N observations: 943 | - |
| 3 | Mito_pooled_ | Fold-change, log-transformed | Training intensity category (three levels, SIT, HIT and ET), initial fitness level (three levels, untrained, moderately trained and well-trained individuals), interaction between intervention weeks (continuous, log transformed before modelling using the natural logarithm) and training intensity category (three slopes), number of training sessions *per* week (continuous, log transformed before modelling using the natural logarithm), active muscle mass during exercise (two levels, small and large active muscle mass exercises), sex (three levels, men, women and mixed; mixed, groups comprising both men and women), disease status (two levels, healthy and diseased participants), and age (three levels, ≤35 years, >35-55 years and >55 years). | Study ID, training group ID, mitochondrial abundance marker (five levels, CS, COX, HADH, SDH and MvD), and allowing for residual variance | All data  N studies: 343  N training groups: 496  N participants: 5511  N observations: 928 | 3 |
| 4 | Mito_pooled_ | Fold-change, log-transformed | Interaction between disease group (four levels, healthy, metabolic diseases, CVD, and COPD) and age (two levels, ≤35 years and >55 years), training intensity category (three levels), interaction between training intensity category and log-transformed intervention weeks (three slopes), and the number of training sessions *per* week (continuous, log transformed before modelling using the natural logarithm) | Study ID, training group ID, mitochondrial abundance marker (five levels, CS, COX, HADH, SDH and MvD), and allowing for residual variance | Only untrained participants. Age >35-55 years were excluded.  N studies: 168  N training groups: 225  N participants: 2396  N observations: 396 | Table 3 |
| 5 | Mito_pooled_ | Fold-change, log-transformed | Interaction between sex (two levels, men and women) and age (two levels, ≤35 years and >55 years), training intensity category (three levels), interaction between training intensity category and log-transformed intervention weeks (three slopes), and the number of training sessions *per* week (continuous, log transformed before modelling using the natural logarithm) | Study ID, training group ID, mitochondrial abundance marker (five levels, CS, COX, HADH, SDH and MvD), and allowing for residual variance | Only untrained, healthy participants were used. Mixed sex groups and age >35-55 years were excluded.  N studies: 101  N training groups: 137  N participants: 1325  N observations: 253 | Table 3 |
| 6 | Mito_pooled_ | Fold-change per hour of training, log-transformed | Interaction between initial fitness level and training intensity category (nine levels, 3 × 3) | Study ID, training group ID, mitochondrial abundance marker (five levels, CS, COX, HADH, SDH and MvD), and allowing for residual variance | All data where training hours was available  N studies: 345  N training groups: 491  N participants: 5488  N observations: 918 | 4 |
| 7 | Capillary-to-fiber ratio | Fold-change, log-transformed | Training intensity category (three levels, SIT, HIT and ET), initial fitness level (three levels, untrained, moderately trained and well-trained individuals), intervention weeks (three levels, ≤4 weeks, >4-8 weeks and >8 weeks) | Study ID, training group ID, and with the residual variance set to unity | All data  N studies: 107  N training groups: 153  N participants: 1825 | 5A,D,G |
| 8 | Capillary density | Fold-change, log-transformed | Training intensity category (three levels, SIT, HIT and ET), initial fitness level (three levels, untrained, moderately trained and well-trained individuals), intervention weeks (three levels, ≤4 weeks, >4-8 weeks and >8 weeks) | Study ID, training group ID, and with the residual variance set to unity | All data  N studies: 98  N training groups: 141  N participants: 1897 | 5B,E,H |
| 9 | Muscle fiber cross-sectional area | Fold-change, log-transformed | Training intensity category (three levels, SIT, HIT and ET), initial fitness level (three levels, untrained, moderately trained and well-trained individuals), intervention weeks (three levels, ≤4 weeks, >4-8 weeks and >8 weeks) | Study ID, training group ID, and with residual variance set to unity | All data  N studies: 54  N training groups: 76  N participants: 886 | 5C,F,I |
| 10 | Capillary-to-fiber ratio | Fold-change, log-transformed | Interaction between disease group (four levels, healthy, metabolic diseases, CVD, and COPD) and age (two levels, ≤35 years and >55 years), the fixed effect of intervention weeks (three levels, ≤4 weeks, >4-8 weeks and >8 weeks) and number of training sessions *per* week (two levels, ≤3 sessions/week and >3 sessions/week) | Study ID, training group ID, and with the residual variance set to unity | Only untrained participants. Age >35-55 years were excluded.  N studies: 56  N training groups: 75  N participants: 845 | Table 3 |
| 11 | Capillary-to-fiber ratio | Fold-change, log-transformed | Interaction between sex (two levels, men and women) and age (two levels, ≤35 years and >55 years), the fixed effect of intervention weeks (three levels, ≤4 weeks, >4-8 weeks and >8 weeks) and number of training sessions *per* week (two levels, ≤3 sessions/week and >3 sessions/week) | Study ID, training group ID, and with the residual variance set to unity | Only untrained, healthy participants were used. Mixed sex groups and age >35-55 years were excluded.  N studies: 37  N training groups: 47  N participants: 504 | Table 3 |
| 12 | Muscle fiber type I proportion | Raw, type I proportion | Interaction between time point (two levels: pre- and post-training) and training intensity category (three levels). | Study ID, training group ID, and allowing for residual variance | All data  N studies: 95  N training groups: 126  N participants: 1455 | 6B |
| 13 | VO_2_max | Percent change | Training intensity category (three levels, SIT, HIT and ET) | None | All data except small muscle mass exercises  N studies: 233  N training groups: 338  N participants: 3945 | - |
| 14 | VO_2_max | Fold-change, log-transformed | Training intensity category (three levels, SIT, HIT and ET), initial fitness level (three levels, untrained, moderately trained and well-trained individuals), interaction between intervention weeks (continuous, log transformed before modelling using the natural logarithm) and training intensity category (three slopes), number of training sessions *per* week (continuous, log transformed before modelling using the natural logarithm), sex (three levels, men, women and mixed; mixed, groups comprising both men and women), disease status (two levels, healthy and diseased participants), and age (three levels, ≤35 years, >35-55 years and >55 years). | Study ID, training group ID, and with the residual variance set to unity | All data except small muscle mass exercises  N studies: 202  N training groups: 298  N participants: 3524 | 7 |
| 15 | VO_2_max | Fold-change, log-transformed | Interaction between disease group (four levels, healthy, metabolic diseases, CVD, and COPD) and age (two levels, ≤35 years and >55 years), training intensity category (three levels), interaction between training intensity category and log-transformed intervention weeks (three slopes), and the number of training sessions *per* week (continuous, log transformed before modelling using the natural logarithm) | Study ID, training group ID, and with the residual variance set to unity | Only untrained participants. Small muscle mass exercises and age >35-55 years were excluded.  N studies: 105  N training groups: 144  N participants: 1639 | Table 3 |
| 16 | VO_2_max | Fold-change, log-transformed | Interaction between sex (two levels, men and women) and age (two levels, ≤35 years and >55 years), training intensity category (three levels), interaction between training intensity category and log-transformed intervention weeks (three slopes), and the number of training sessions *per* week (continuous, log transformed before modelling using the natural logarithm) | Study ID, training group ID, and with the residual variance set to unity | Only untrained, healthy participants were used. Small muscle mass exercises, mixed sex groups and age >35-55 years were excluded.  N studies: 105  N training groups: 144  N participants: 1639 | Table 3 |
| 17 | VO_2_max | Fold-change per hour of training, log-transformed | Interaction between initial fitness level and training intensity category (nine levels, 3 × 3) | Study ID, training group ID, and with the residual variance set to unity | All data where training hours was available except small muscle mass exercises  N studies: 202  N training groups: 295  N participants: 3487 | 8 |
