# Supplementary material for: Effects of Exercise Training on Mitochondrial and Capillary Growth in Human Skeletal Muscle: A Systematic Review and Meta-Regression
Source: Sports Med. 2024 Oct 10;55(1):115–44. doi: 10.1007/s40279-024-02120-2 (PMC11787188; doi:10.1007/s40279-024-02120-2)
Supplement: Supplementary file 2 — Supplementary file2 Supplementary Information 2: Analysis of citrate synthase (DOCX 197 KB) [file 40279_2024_2120_MOESM2_ESM.docx]

**Supplement 2, separate analysis for citrate synthase content-only:**

**Statistics**

Log-transformed (natural logarithm) fold-changes in citrate synthase (CS) from pre- to post-training was analyzed using the generalized linear-mixed model procedure (GLIMMIX) with a specified Gaussian distribution in SAS OnDemand for Academics (SAS Studio 3.8, SAS Institute Inc., Cary, NC). The effect estimates were weighted by the inverse of the square of the studies’ standard errors (1/SE^2^). When the standard error (SE) of the fold-change was missing, it was derived when possible from the standard deviation (SD) or 95% confidence limits (CL), or from t statistics calculated from corresponding exact P-values (Higgins et al. 2021):

SE = $\frac{Mean change}{t}$ eq. 1

In cases of no presented variability on the fold-changes, the SD was imputed if the pre- and post-training mean and SD were presented (Higgins et al. 2021):

SD_change_ = $\frac{\sqrt{{SD}_{pre}^{2}+{SD}_{post}^{2} - (2 \times r \times{SD}_{pre} \times{SD}_{post})}}{{Mean}_{pre}}$ eq. 2

In this calculation, the correlation coefficient (r) describing the similarity between the pre- and post-data across participants was calculated in each study reporting SD_pre_, SD_post_ and SD_change_, and the median value was subsequently used in equation 2 (r = 0.61, based on 105 studies):

r = $\frac{{SD}_{pre}^{2} + {SD}_{post}^{2} - {SD}_{change}^{2}}{{2 \times{SD}_{pre} \times SD}_{post}}$ eq. 3

Models were constructed using the log-transformed fold-change in CS as the dependent variable:

*Model 2* (named with the same number according to the comparable model for mito_pooled_) included the fixed effect of training intensity category, did not include any random effects, and used raw percent change scores as the dependent variable (unadjusted fixed effect model). *Model 3* included the fixed effects of training intensity category (three levels, SIT, HIT and ET), training intensity category interacted by the log-transformed (natural logarithm) number of training intervention weeks (continuous, giving three slopes), initial fitness level (three levels, untrained, moderately trained and well-trained individuals), the log-transformed number of training sessions *per* week (continuous, giving one slope), active muscle mass during exercise (two levels, small and large active muscle mass exercises; see 2.4.5 of the manuscript for explanation), sex (three levels, men, women and mixed; mixed, groups comprising both men and women), disease status (two levels, healthy and diseased participants), age (three levels, ≤35 years, >35-55 years and >55 years) and enzyme analysis type (two levels: enzyme content using Western blot and enzyme activity). To further test the impact of different disease groups, *Model 4* included an interaction between disease group (four levels, healthy, metabolic diseases, CVD, and COPD) and age (two levels, ≤35 years and >55 years; those >35-55 years were excluded). The above model also included the fixed effect of training intensity category, an interaction between training intensity category and the log-transformed number of training intervention weeks (giving three slopes) and the log-transformed number of training sessions *per* week (giving one slope), and the model was only run on data from previously untrained participants. These decisions were made due to the findings in *Model 3* (i.e., to account for covariates that had a significant effect). To test the impact of menopause on training responses, *Model 5* included the interaction between sex (two levels, men and women, the mixed sex group was excluded) and age (two levels, ≤35 years and >55 years, where those >35-55 years were excluded to avoid groups including both pre- and postmenopausal women) and included the same covariate adjustments as in model 4. This model was only run on data from untrained, healthy participants. In *Model 6*, the dependent variable was the fold-change in CS divided by the total number of hours of exercise (a data step carried out before modelling), and the model included the interaction effect between initial fitness level and training intensity category (nine levels, 3 × 3). Hence, in model 3-5, the change in CS was adjusted for the total number of training sessions, while a normalization for the total hours of exercise training was carried out for *Model 6* to compare training efficiency between training intensity categories. All models included the random effects of study ID and training group ID to allow for different effect estimates between studies and between different groups of participants within the same study. Estimated marginal means ± 95% CI were back-transformed and expressed as percentage changes and percentage changes *per* hour of exercise training. For models including several classification effects, estimated marginal means for each classification effect were calculated based on the observed weight of the other classification effects and the mean value of continuous fixed effects (the OBSMARGINS option within the LSMEANS statement or weighted the same way within the ESTIMATE statement) due to the unbalanced des**i**gn. The impact of continuous fixed effects on the dependent variable was estimated and graphed from the 10^th^ to the 90^th^ percentile of its frequency distribution. For the impact of intervention weeks on CS abundance, this implicated plotting the estimated marginal means from 2-11 weeks, 2-13 weeks and 2-23 weeks of training for SIT, HIT and ET, respectively. Similarly, the estimated marginal means for 2-6 sessions/week was graphed. Only planned pairwise comparisons were conducted using the ESTIMATE statement, and the Holm-Bonferroni method was used to adjust *P* values, while the Bonferroni method was used to adjust 95% CI for multiple comparisons in a row-wise and column-wise fashion.

**Results**

Unadjusted for covariates, not log-transformed, non-weighted, and pooled with a fixed effect model, the mean change in CS content were 29.8 ± 4.0 %, 30.5 ± 4.7 % and 22.6 ± 7.2 % for ET, HIT and SIT, respectively (Model 2). Adjusting for covariates (intervention weeks, training frequency, initial fitness level, active muscle mass while training, disease status, sex, enzyme analysis type and age) and using appropriate weighting, all training intensity categories were associated with increases in CS content (ET, 23.8 ± 3.5 %; HIT, 26.7 ± 4.2 %; SIT, 26.9 ± 7.4 %; all *P* < 0.001; *Model 3*). Of note, the estimated marginal means comparing the training intensity categories are slightly different from the mean changes presented above due to weighting, log-transformation before modelling, and adjustments for covariates, especially since SIT studies in average were shorter and displayed lower training frequency (see Table 1 of the manuscript). When studying the time course of adaptation, all training intensity categories increased CS content after only two weeks of training (ET, 13.7 ± 6.0 %; HIT, 14.0 ± 7.1 %; SIT, 26.1 ± 10.5 %; all *P* < 0.001; Fig. S1A; *Model 3*). The increase in CS content by intervention weeks followed log-linear relationships, with ET and HIT showing significant increases between 2-6 weeks and 6-10 weeks of training (all, *P* < 0.001). However, SIT did not alter CS content after the initial change occurring within 2 weeks of training (*P* = 1.00; Fig. S1A). The training frequency had a log-linear impact on CS content, with six sessions/week being more potent than four and four sessions/week being more potent than two (both, P < 0.001; Fig. S1B; Model 3). CS content only showed a tendency to increase in well-trained participants (6.3 ± 7.7 %; *P* = 0.098; Supplementary Fig. S1C; *Model 3*), and increased to a greater extent in previously moderately trained participants compared to well-trained participants (mean difference: Δ 15.8 ± 11.4 %-points; *P* < 0.001; Supplementary Fig. S1C), and the largest improvements were seen in previously untrained participants (untrained vs. moderately trained: Δ 6.5 ± 6.8 %-points; *P* = 0.018; Supplementary Fig. S1C). CS content increased slightly more when measured as enzyme activity than as protein expression using Western blots (mean difference: Δ 1.8 ± 1.1 %-points; *P* = 0.0012; Supplementary Fig. S1D; *Model 3*). CS content increased to a similar extent with exercise training engaging whole-body exercises and small muscle mass exercises (Supplementary Fig. S1E; *P* = 0.446; *Model 3*). The exercise training response was neither affected by disease status (*P* = 0.956; Supplementary Fig. S1F), sex (*P* = 0.236; Supplementary Fig. S1G) nor age (*P* = 0.908; Supplementary Fig. S1H; *Model 3*).

<< Supplementary Fig. S1 here >>

When split into disease groups, neither young participants (< 35 years) with metabolic diseases (*P* = 0.180) nor old participants (> 55 years) with metabolic diseases (*P* = 0.328), CVD (*P* = 0.751), or COPD (*P* = 0.759) responded differently to exercise training compared to healthy, age-matched, and initial fitness level-matched (i.e., only untrained) participants, when training intensity, -frequency and -intervention weeks were controlled for (Supplementary Fig. S2A; *Model 4*). Furthermore, old, healthy, untrained women (P = 0.939) and men (P = 0.646) increased CS content to a similar extent as their young peers when controlled for training intensity, -frequency and -intervention weeks (Supplementary Fig. S2B; *Model 5*).

<< Supplementary Fig. S2 here >>

CS content *per hour of exercise training (exercise training efficiency)*

When normalizing the percent change in CS content to total hours of exercise training (Supplementary Fig. S3), SIT was ~2.3 times more efficient than HIT and ~3.7 times more efficient than ET (both, *P* < 0.001), while HIT was ~1.6 times more efficient than ET (*P* = 0.029; *Model 6*) as a weighted mean of all initial fitness level groups. The same trend of progressively greater efficiency ET < HIT < SIT was also seen when the dataset was stratified into initial fitness level groups (Supplementary Fig. S3; *Model 6*). Untrained and moderately trained individuals increased CS content per hour of exercise training for all training intensity categories (all, P < 0.01). However, well-trained individuals did not show any change in CS content when expressed *per* hour of exercise training after ET (*P* = 0.554), HIT (*P* = 0.549) and SIT (*P* = 0.085).

<< Supplementary Fig. S3 here >>

| **Supplementary Fig. S1:** The effects of intervention weeks by training intensity category (A), training frequency (B), initial fitness level (C), enzyme measurement type (D), amount of recruited muscle mass during exercise (E), disease status (F), sex (G), and age (H), on training-induced changes (Δ) in citrate synthase content (Model 3). ET, endurance training; HIT, high-intensity interval training; SIT, sprint interval training. Values are estimated marginal means with 95% confidence limits (N training groups = 409). |
| --- |
|  |

| **Supplementary Fig. S2:** The interaction between age and disease group (A; Model 4) and sex (B; Model 5) on training-induced changes (Δ) in citrate synthase content adjusted for training intensity, training frequency and the interaction between training intensity and intervention weeks. Only previously untrained participants were used in A and B. In B, data from only healthy participants were used. COPD, chronic obstructive pulmonary disease; CVD, cardiovascular diseases. Values are estimated marginal means with 95% confidence limits. P-values in A denote the comparison between disease groups and the healthy age-matched group. Numbers in parentheses denotes the number of training groups in each subgroup (young/old). |
| --- |
|  |

| **Supplementary Fig. S3:** The interaction effect of initial fitness level and training intensity category on changes (Δ) in citrate synthase content per training hour (Model 6). ET, endurance training; HIT, high-intensity interval training; SIT, sprint interval training. Values are estimated marginal means with 95% confidence limits. (N training groups and observations = 404). |
| --- |
|  |

**References**

Higgins JPT, Li T, Deeks JJ, (editors) (2021) Chapter 6: Choosing effect measures and computing estimates of effect. In: Higgins JPT et al. (eds) Cochrane Handbook for Systematic Reviews of Interventions version 6,2 (updated February 2021). Cochrane.
