# Supplementary material for: Effects of Exercise Training on Mitochondrial and Capillary Growth in Human Skeletal Muscle: A Systematic Review and Meta-Regression
Source: Sports Med. 2024 Oct 10;55(1):115–44. doi: 10.1007/s40279-024-02120-2 (PMC11787188; doi:10.1007/s40279-024-02120-2)
Supplement: Supplementary file 3 — Supplementary file3 Supplementary Information 3: Analysis of absolute changes in capillary markers and VO2max (DOCX 405 KB) [file 40279_2024_2120_MOESM3_ESM.docx]

***Supplement 3, separate analysis for absolute changes in capillary markers and* V̇O_2_max**

**Statistics:**

See the main text document and Supplementary Information 1 (Statistical models summary) for specifications regarding the statistical models. The models for absolute changes in capillary markers and V̇O_2_max were run identically to those investigating % changes in these dependent variables, except using absolute change scores instead of log-transformed fold-changes. In addition, no back-transformation was needed after modelling since data were not log-transformed before modelling.

**Results:**

***The effect of exercise training on muscle capillarization* and CSA (absolute changes)**

C/F increased (ΔN/N) after ET (0.27 ± 0.04; *P* < 0.001), HIT (0.20 ± 0.06; *P* < 0.001) and SIT (0.22 ± 0.19; *P* = 0.025), with no significant differences between the exercise intensity categories (*P* > 0.255; Supplementary (S) Fig. S4A; Similar to m*odel 7* in the main text). CD only increased (Δ capillaries per mm^2^) after ET (42 ± 9; *P* < 0.001) and HIT (19 ± 13; *P* = 0.004), with the change being significantly larger with ET compared to HIT (Δ 23 ± 18; *P* = 0.009) and tended to be larger than SIT (Δ 34 ± 42; *P* = 0.101; Fig. S4B; *Model 8*). These estimated marginal means are slightly different from the mean of the training groups’ individual changes for C/F and CD (Fig. S4J-K) due to adjustments for covariates (initial fitness level and intervention weeks) and appropriate weighting. C/F increased in previously untrained (0.24 ± 0.04; *P* < 0.001) and moderately trained participants (0.33 ± 0.10; P < 0.001), while well-trained participants did not change C/F in response to exercise training (0.09 ± 0.18; *P* = 0.361; Fig. S4D). Similarly, exercise training increased CD (Fig. S4E) for both untrained (34 ± 9; P < 0.001) and moderately trained (43 ± 19; P < 0.001), but not in well-trained individuals (-2 ± 39; P = 0.919). After only ≤ 4 weeks of exercise training, increases in C/F (0.21 ± 0.13; *P* = 0.002) and CD (58 ± 22; *P* < 0.001) were observed, but a higher number of intervention weeks was not associated with any further changes (Fig. S4G and S4H; P > 0.463 for CF and P > 0.067 for CD). The increase in C/F tended to be lower in participants with metabolic diseases than in healthy, age- and training-status-matched young (*P* = 0.072; Fig. S5A; *Model 10*) but not old (P=0.567) participants. Old participants with COPD increased the C/F less than healthy age- and training-status-matched participants (P=0.051), and participants with CVD almost tended to do the same (P=0.128). Both male and female participants >55 years of age increased C/F in a comparable manner as their young counterparts (*P* = 0.957 and *P* = 0.597, respectively; Fig. S5B; *Model 11*). Exercise training did not alter muscle fiber CSA in any training intensity category (P > 0.188), fitness level group (P > 0.151), nor at any training duration (P > 0.168; Fig. S4C, F, I; *Model 9*). Of note, the latter contrasts, to some extent, the percentage increases due to large between-study variations in absolute levels of muscle fiber CSA.

<< Supplementary Fig. S4-5 here >>

***The effect of exercise training on maximal oxygen consumption* (Δ ml/kg/min)**

In Fig. S6A, the individual changes in body weight normalized V̇O_2_max (Δ ml/kg/min) for all training groups are presented and divided into training intensity categories. Unadjusted for covariates and pooled with a fixed effect model, the mean change in V̇O_2_max were 4.3 ± 0.4, 4.2 ± 0.5 and 2.9 ± 0.8 ml/kg/min for ET, HIT and SIT, respectively. Adjusted for covariates (to mean intervention weeks and -frequency, initial fitness level, disease status, sex, and age; *Model 14*), all training intensity categories were associated with increases in V̇O_2_max (ET, 3.6 ± 0.3 ml/kg/min; HIT, 4.2 ± 0.5 ml/kg/min; SIT, 2.4 ± 1.0 ml/kg/min; all *P* < 0.001; similar to *Model 14* in the main text), with a significantly larger increase for HIT than ET (P=0.046) and SIT (P=0.004) and larger for ET than SIT (P=0.044). When studying the time course of adaptation, all training intensity categories increased V̇O_2_max after only two weeks of training (ET, 1.3 ± 0.8 ml/kg/min; HIT, 1.8 ± 1.2 ml/kg/min; SIT, 2.7 ± 1.3 ml/kg/min; all *P* < 0.01; Fig. S6B; *Model 14*). The increase in V̇O_2_max by intervention weeks followed log-linear relationships, with ET and HIT showing significant increases in V̇O_2_max between 2-6 weeks and 6-10 weeks of training (all, *P* < 0.001). However, SIT did not alter V̇O_2_max after the initial change occurring within 2 weeks of training (*P* = 1.00; Fig. S6B). The training frequency had a log-linear impact on V̇O_2_max, with 6 sessions/week being more potent than four and four sessions/week being more potent than two (both, P < 0.001; Fig. S6C; *Model 14*). V̇O_2_max increased with exercise training irrespective of initial fitness level, including well-trained participants (1.1 ± 1.1 ml/kg/min; *P* = 0.057; Fig. S6D). However, V̇O_2_max increased to a greater extent in previously moderately trained participants compared to well-trained participants (mean difference: Δ 1.8 ± 1.5 ml/kg/min; *P* = 0.007; Fig. S6D), and more in previously untrained participants compared to moderately trained participants (Δ 1.0 ± 0.9 ml/kg/min; *P* = 0.008; Fig. S6D) and compared to well-trained participants (Δ 2.8 ± 1.5 ml/kg/min; *P* < 0.001; Fig. S6D; *Model 14*). Participants with a disease (all diseased pooled) responded poorer than healthy participants (Δ -0.6 ± 0.6 ml/kg/min; *P* = 0.039; Fig. S6E; *Model 14*). The exercise training response was not affected by sex (P=0.976; Fig. S6F; Model 14). However, those younger than 35 years responded better than those aged 35-55 years (Δ 2.0 ± 1.0 ml/kg/min; *P* < 0.001; Fig. S6G) and > 55 years (Δ 2.7 ± 0.9 ml/kg/min; *P* < 0.001; Fig. S6G). When split into disease groups, neither young participants (< 35 years) with metabolic diseases (*P* = 0.423) nor old participants (> 55 years) with metabolic diseases (*P* = 0.566), CVD (*P* = 0.099), or COPD (*P* = 0.566) responded differently to exercise training compared to healthy, age-matched and initial fitness level-matched (i.e., only untrained) participants, when training intensity, training frequency and intervention weeks were controlled for (Fig. S7A; *Model 15*). Young (< 35 years), healthy, untrained women and men increased their V̇O_2_max more than older (>55years) sex-, health- and fitness status matched groups (P ≤ 0.001) after controlling for training intensity, training frequency and intervention weeks (Fig. S7B; *Model 16*). No difference between sex was observed in young (P=0.382) nor old groups (P=0.347) in contrast to percentage changes as presented within the main manuscript.

<< Supplementary Fig. S6-7 here >>

| Fig. S4: Absolute change (Δ) in capillary-to-fiber ratio (Similar to model 7 in the main text), capillary density (Model 8) and muscle fiber cross-sectional area (Model 9) in response to exercise training. In the upper panel, the effects of training intensity on training-induced changes in capillary-to-fiber ratio (A), capillary density (B) and muscle fiber cross-sectional area (C) are shown. In the second panel (D-F), the effects of initial fitness level are displayed, while in the third panel (G-I), the impact of training intervention duration on the same variables are presented. In J-L, the individual changes (Δ) in capillary-to-fiber ratio (C/F), capillary density (CD), and muscle fiber cross-sectional area (CSA) from before to after a period of ET, HIT and SIT. For the number of training groups and participants, see Table 2 in the main text. Values are estimated marginal means with 95% confidence limits. *, *P* < 0.05; ET, endurance training; HIT, high-intensity interval training; SIT, sprint interval training. |
| --- |
|  |
| Fig. S5: The interaction between age and disease group (A; Model 10 in the main text) and sex (B: Model 11) on training-induced absolute changes (Δ N/N) in capillary-to-fiber ratio. Only previously untrained participants were included. In B, data from only healthy participants were included. COPD, chronic obstructive pulmonary disease; CVD, cardiovascular diseases. Values are estimated marginal means with 95% confidence limits. P-values in A denote the comparison between disease groups and the healthy age-matched group. Numbers in A (N studies = 56, N training groups/observations = 75, N participants = 845, where the numbers in parentheses denotes the number of training groups in each subgroup (young/old)) and numbers in B (N studies = 37, N training groups/observations = 47, N participants = 504, where the numbers in parentheses denotes the number of training groups in each subgroup (young/old)). |
|  |

| Fig. S6: In A, the unadjusted individual raw changes (Δ ml/kg/min) in maximal oxygen consumption (V̇O_2_max) are presented and divided into training intensity categories (ET, endurance training; HIT, high-intensity interval training; SIT, sprint interval training). The remaining figures show the effects of intervention weeks for each training intensity category (B), training frequency (C), initial fitness level (D), disease status (E), sex (F), and age (G), on training-induced changes (Δ ml/kg/min) in V̇O_2_max after adjusting for covariates (similar to Model 14 in the main text). Values are estimated marginal means with 95% confidence limits (N studies = 208, N training groups = 302, N participants = 3632). |
| --- |
|  |

| Fig. S7: The interaction between age and disease group (A; Model 15) and sex (B; Model 16) on training-induced changes (Δ ml/kg/min) in V̇O_2_max adjusted for training intensity, training frequency and the interaction between training intensity and intervention weeks. Only previously untrained participants were included in A and B. In B, data from only healthy participants were included. COPD, chronic obstructive pulmonary disease; CVD, cardiovascular diseases. Values are estimated marginal means with 95% confidence limits. P-values in B denote the comparison between disease groups and the healthy age-matched group. Numbers in A (N studies = 105, N training groups = 146, N participants = 1658, where the numbers in parentheses denotes the number of training groups in each subgroup (young/old)) and numbers in B (N studies = 69, N training groups = 95, N participants = 984, where the numbers in parentheses denotes the number of training groups in each subgroup (young/old)). |
| --- |
|  |
