# Supplementary material for: Effects of Exercise Training on Mitochondrial and Capillary Growth in Human Skeletal Muscle: A Systematic Review and Meta-Regression
Source: Sports Med. 2024 Oct 10;55(1):115–44. doi: 10.1007/s40279-024-02120-2 (PMC11787188; doi:10.1007/s40279-024-02120-2)
Supplement: Supplementary file 4 — Supplementary file4 Supplementary Information 4: Reference list of research articles included in mitochondrial analysis (PDF 248 KB) [file 40279_2024_2120_MOESM4_ESM.pdf]

References to research articles in $\text{Mito}_{\text{pooled}}$

1. Varnauskas E, Björntorp P, Fahlén M, Prerovský I, Stenberg J. Effects of physical training on exercise blood flow and enzymatic activity in skeletal muscle. Cardiovasc Res [Internet]. 1970;4:418–22. Available from: http://www.ncbi.nlm.nih.gov/pubmed/5533086

2. Gollnick PD, Armstrong RB, Saltin B, Saubert CW, Sembrowich WL, Shepherd RE. Effect of training on enzyme activity and fiber composition of human skeletal muscle. J Appl Physiol. 1973;34:107–11.

3. Eriksson BO, Gollnick PD, Saltin B. Muscle Metabolism and Enzyme Activities after Training in Boys 11–13 Years Old. Acta Physiol Scand. 1973;87:485–97.

4. Kiessling KH, Pilström L, Karlsson J, Piehl K. Mitochondrial volume in skeletal muscle from young and old physically untrained and trained healthy men and from alcoholics. Clin Sci [Internet]. 1973;44:547–54. Available from: http://www.ncbi.nlm.nih.gov/pubmed/4713662

5. Bjorntorp P, de Jounge K, Sjostrom L, Sullivan L. Physical training in human obesity. II. Effects on plasma insulin in glucose-intolerant subjects without marked hyperinsulinemia. ScandJ Clin Lab Invest. 1973;32:41–5.

6. Bergman H, Björntorp P, Conradson T -B, Fahlén M, Stenberg J, Vamauskas E. Enzymatic and Circulatory Adjustments to Physical Training in Middle-Aged Men. Eur J Clin Invest. 1973;3:414–8.

7. Saltin B, Nazar K, Costill DL, Stein E, Jansson E, Essén B, et al. The nature of the training response; peripheral and central adaptations of one-legged exercise. Acta Physiol Scand [Internet]. 1976;96:289–305. Available from: http://www.ncbi.nlm.nih.gov/pubmed/132082

8. Henriksson J, Reitman JS. Quantitative measures of enzyme activities in type I and type II muscle fibres of man after training. Acta Physiol Scand [Internet]. 1976;97:392–7. Available from: http://www.ncbi.nlm.nih.gov/pubmed/134623

9. Howald H. Ultrastructure and biochemical function of skeletal muscle in twins. Ann Hum Biol. 1976;3:455–62.

10. Henriksson J, Reitman JS. Time course of changes in human skeletal muscle succinate dehydrogenase and cytochrome oxidase activities and maximal oxygen uptake with physical activity and inactivity. Acta Physiol Scand [Internet]. 1977 [cited 2015 Sep 27];99:91–7. Available from: http://www.ncbi.nlm.nih.gov/pubmed/190867

11. Andersen P, Henriksson J. Capillary supply of the quadriceps femoris muscle of man: adaptive response to exercise. J Physiol. 1977;270:677–90.

12. Henriksson J. Training induced adaptation of skeletal muscle and metabolism during submaximal exercise. J Physiol. 1977;270:661–75.

13. Suominen H, Heikkinen E, Liesen H, Michel D, Hollmann W. Effects of 8 weeks' endurance training on skeletal muscle metabolism in 56-70-year-old sedentary men. Eur J Appl Physiol Occup Physiol [Internet]. 1977;37:173–80. Available from: http://www.ncbi.nlm.nih.gov/pubmed/913382

14. Örlander J, Kiessling K-H, Karlsson J, Ekblom B. Low Intensity Training, Inactivity and Resumed Training in Sedentary Men. Acta Physiol Scand [Internet]. 1977 [cited 2015 Sep 28];101:351–62. Available from: http://www.ncbi.nlm.nih.gov/pubmed/596209

15. Saltin B, Lindgarde F, Houston M, Hörlin R, Nygaard E, Gad P. Physical training and glucose tolerance in middle-aged men with chemical diabetes. Diabetes. 1979;28:30–2.

16. ÖRLANDER J, KIESSLING K -H, EKBLOM B. Time course of adaptation to low intensity training in sedentary men: dissociation of central and local effects. Acta Physiol Scand. 1980;108:85–90.

17. Orlander J, Aniansson A. Effect of physical training on skeletal muscle metabolism and ultrastructure in 70 to 75-year-old men. Acta Physiol Scand [Internet]. 1980;109:149–54. Available from: http://www.ncbi.nlm.nih.gov/pubmed/6252748

18. Klausen K, Andersen LB, Pelle I. Adaptive changes in work capacity, skeletal muscle capillarization and enzyme levels during training and detraining. Acta Physiol Scand [Internet]. 1981;113:9–16. Available from: http://www.ncbi.nlm.nih.gov/pubmed/7315443

19. Sjödin B, Jacobs I, Svedenhag J. Changes in onset of blood lactate accumulation (OBLA) and muscle enzymes after training at OBLA. Eur J Appl Physiol Occup Physiol. 1982;49:45–57.

20. Fournier M, Ricci J, Taylor AW, Ferguson RJ, Montpetit RR, Chaitman BR. Skeletal muscle adaptation in adolescent boys: sprint and endurance training and detraining. Med Sci Sports Exerc. 1982;14:453–6.

21. Roberts AD, Billeter R, Howald H. Anaerobic muscle enzyme changes after interval training. Int J Sports Med [Internet]. 1982;3:18–21. Available from: https://www.thieme-connect.com.ubproxy.ub.uni-heidelberg.de/ejournals/pdf/sportsmed/doi/10.1055/s-2008-1026055.pdf%5Cnpapers3://publication/uuid/85521B0B-A98C-4794-9877-39A7A5B1CB34

22. Daub WD, Green HJ, Houston ME, Thomson JA, Fraser IG, Ranney DA. Cross-adaptive responses to different forms of leg training: skeletal muscle biochemistry and histochemistry. Can J Physiol Pharmacol [Internet]. 1982;60:628–33. Available from: http://www.ncbi.nlm.nih.gov/pubmed/7104850

23. Wallberg-Henriksson H, Gunnarsson R, Henriksson J, DeFronzo R, Felig P, Ostman J, et al. Increased peripheral insulin sensitivity and muscle mitochondrial enzymes but unchanged blood glucose control in type I diabetics after physical training. Diabetes [Internet]. 1982;31:1044–50. Available from: http://www.ncbi.nlm.nih.gov/pubmed/6757018

24. Svedenhag J, Henriksson J, Sylvén C. Dissociation of training effects on skeletal muscle mitochondrial enzymes and myoglobin in man. Acta Physiol Scand [Internet]. 1983 [cited 2015 Sep 28];117:213–8. Available from: http://www.ncbi.nlm.nih.gov/pubmed/6306998

25. Krotkiewski M, Bylund-Fallenius AC, Holm J, Bjorntorp P, Grimby G, Mandroukas K. Relationship between muscle morphology and metabolism in obese women: the effects of long-term physical training. Eur J Clin Invest [Internet]. 1983;13:5–12. Available from: http://www.ncbi.nlm.nih.gov/entrez/query.fcgi?cmd=Retrieve&db=PubMed&dopt=Citation&list_uids=6409624

26. Schantz P, Henriksson J, Jansson E. Adaptation of human skeletal muscle to endurance training of long duration. Clin Physiol [Internet]. 1983;3:141–51. Available from: http://www.ncbi.nlm.nih.gov/pubmed/6682735

27. Seals DR, Sinacore DR, Hurley BF, Nemeth PM, Hagberg JM. Failure of endurance training to alter the cardiovascular response to static contraction. Clin Physiol. 1983;3:219–26.

28. Mandroukas K, Krotkiewski M, Hedberg M, Wroblewski Z, Björntorp P, Grimby G. Physical training in obese women. Effects of muscle morphology, biochemistry and function. Eur J Appl Physiol Occup Physiol [Internet]. 1984;52:355–61. Available from: http://www.ncbi.nlm.nih.gov/pubmed/6381044

29. Wallberg-Henriksson H, Gunnarsson R, Henriksson J. Influence of physical training on formation of muscle capillaries in type I diabetes. Diabetes. 1984;33:851–7.

30. Sjøgaard G. Muscle morphology and metabolic potential in elite road cyclists during a season. Int J Sports Med. 1984;5:250–4.

31. Rösler K, Hoppeler H, Conley KE, Claassen H, Gehr P, Howald H. Transfer effects in endurance exercise. Adaptations in trained and untrained muscles. Eur J Appl Physiol Occup Physiol [Internet]. 1985;54:355–62. Available from: http://www.ncbi.nlm.nih.gov/pubmed/4065122

32. Howald H, Hoppeler H, Claassen H, Mathieu O, Straub R. Influences of endurance training on the ultrastructural composition of the different muscle fiber types in humans. Pfl??gers Arch Eur J Physiol. 1985;403:369–76.

33. Hurley BF, Nemeth PM, Martin WH, Hagberg JM, Dalsky GP, Holloszy JO. Muscle triglyceride utilization during exercise: effect of training. J Appl Physiol [Internet]. 1986;60:562–7. Available from: http://www.ncbi.nlm.nih.gov/pubmed/3512511

34. Mandroukas K, Krotkiewski M, Holm G, Strömblad G, Grimby G, Lithell H, et al. Muscle adaptations and glucose control after physical training in insulin-dependent diabetes mellitus. Clin Physiol [Internet]. 1986;6:39–52. Available from: http://search.ebscohost.com/login.aspx?direct=true&db=sph&AN=SPH193187&site=ehost-live

35. Simoneau J a, Lortie G, Boulay MR, Marcotte M, Thibault MC, Bouchard C. Inheritance of human skeletal muscle and anaerobic capacity adaptation to high-intensity intermittent training. Int J Sports Med [Internet]. 1986;7:167–71. Available from: http://www.ncbi.nlm.nih.gov/pubmed/3733313

36. Wolfel EE, Hiatt WR, Brammell HL, Carry MR, Ringel SP, Travis V, et al. Effects of selective and nonselective beta-adrenergic blockade on mechanisms of exercise conditioning. Circulation [Internet]. 1986;74:664–74. Available from: http://circ.ahajournals.org/cgi/doi/10.1161/01.CIR.74.4.664

37. Hamel P, Simoneau JA, Lortie G, Boulay MR, Bouchard C. Heredity and muscle adaptation to endurance training. Med Sci Sports Exerc. 1986;18:690–6.

38. Simoneau JA, Lortie G, Boulay MR, Marcotte M, Thibault MC, Bouchard C. Effects of two high-intensity intermittent training programs interspaced by detraining on human skeletal muscle and performance. Eur J Appl Physiol Occup Physiol. 1987;56:516–21.

39. Jacobs I, Esbjörnsson M, Sylvén C, Holm I, Jansson E. Sprint training effects on muscle myoglobin, enzymes, fiber types,

and blood lactate. Med Sci Sports Exerc [Internet]. 1987;19:368–74. Available from: http://www.ncbi.nlm.nih.gov/pubmed/2958671

40. Moore RL, Thacker EM, Kelley G a, Musch TI, Sinoway LI, Foster VL, et al. Effect of training/detraining on submaximal exercise responses in humans. J Appl Physiol [Internet]. 1987;63:1719–24. Available from: http://www.ncbi.nlm.nih.gov/pubmed/3693207

41. Terrados N, Melichna J, Sylven C, Jansson E, Kaijser L. Effects of training at simulated altitude on performance and muscle metabolic capacity in competitive road cyclists. Eur J Appl Physiol Occup Physiol [Internet]. 1988;57:203–9. Available from: http://www.ncbi.nlm.nih.gov/pubmed/3349988

42. Allenberg K, Johansen K, Saltin B. Skeletal muscle adaptations to physical training in type II (non-insulin-dependent) diabetes mellitus. Acta Med Scand. 1988;223:365–73.

43. Martin WH, Coggan AR, Spina RJ, Saffitz JE. Effects of fiber type and training on beta-adrenoceptor density in human skeletal muscle. Am J Physiol [Internet]. 1989;257:E736-42. Available from: http://www.ncbi.nlm.nih.gov/pubmed/2556938

44. Sale DG, MacDougall JD, Jacobs I, Garner S. Interaction between concurrent strength and endurance training. J Appl Physiol [Internet]. 1990;68:260–70. Available from: http://www.scopus.com/inward/record.url?eid=2-s2.0-0025014889&partnerID=40&md5=2bdd11f7215f5b8607f78613bc08dd80

45. Mizuno M, Juel C, Bro-Rasmussen T, Mygind E, Schibye B, Rasmussen B, et al. Limb skeletal muscle adaptation in athletes after training at altitude. J Appl Physiol. 1990;68:496–502.

46. Nelson AG, Arnall DA, Loy SF, Silvester J. Consequences of Combining Strength and Endurance Training Regimens. Phys Ther. 1990;70:287–94.

47. Terrados N, Jansson E, Sylvén C, Kaijser L. Is hypoxia a stimulus for synthesis of oxidative enzymes and myoglobin? J Appl Physiol. 1990;68:2369–72.

48. Kaijser L, Sundberg CJ, Eiken O, Nygren a, Esbjörnsson M, Sylvén C, et al. Muscle oxidative capacity and work performance after training under local leg ischemia. J Appl Physiol. 1990;69:785–7.

49. Cadefau J, Casademont J, Grau JM, Fernández J, Balaguer a, Vernet M, et al. Biochemical and histochemical adaptation to sprint training in young athletes. Acta Physiol Scand. 1990;140:341–51.

50. Gorostiaga EM, Walter CB, Foster C, Hickson RC. Uniqueness of interval and continuous training at the same maintained exercise intensity. Eur J Appl Physiol Occup Physiol [Internet]. 1991;63:101–7. Available from: http://www.ncbi.nlm.nih.gov/pubmed/1748098

51. Green HJ, Jones S, Ball-Burnett ME, Smith D, Livesey J, Farrance BW. Early muscular and metabolic adaptations to prolonged exercise training in humans. J Appl Physiol. 1991;70:2032–8.

52. Keith SP, Jacobs I, McLellan TM. Adaptations to training at the individual anaerobic threshold. Eur J Appl Physiol Occup Physiol. 1992;65:316–23.

53. Neary JP, Martin TP, Reid DC, Burnham R, Quinney HA. The effects of a reduced exercise duration taper programme on performance and muscle enzymes of endurance cyclists. Eur J Appl Physiol Occup Physiol [Internet]. 1992;65:30–6. Available from: http://www.ncbi.nlm.nih.gov/pubmed/1505537

54. Green HJ, Helyar R, Ball-Burnett M, Kowalchuk N, Symon S, Farrance B. Metabolic adaptations to training precede changes in muscle mitochondrial capacity. J Appl Physiol [Internet]. 1992;72:484–91. Available from: http://eutils.ncbi.nlm.nih.gov/entrez/eutils/elink.fcgi?dbfrom=pubmed&id=1559923&retmode=ref&cmd=prlinks%5Cnpapers2://publication/uuid/B2CC699E-BEA3-43E0-A92C-B710CCBDC1AC

55. Coggan a R, Spina RJ, King DS, Rogers M a, Brown M, Nemeth PM, et al. Skeletal muscle adaptations to endurance training in 60- to 70-yr-old men and women. J Appl Physiol. 1992;72:1780–6.

56. Wibom R, Hultman E, Johansson M, Matherei K, Constantin-Teodosiu D, Schantz PG. Adaptation of mitochondrial ATP production in human skeletal muscle to endurance training and detraining. J Appl Physiol [Internet]. 1992;73:2004–10. Available from: http://jap.physiology.org/content/73/5/2004.short

57. Coggan AR, Spina RJ, Kohrt WM, Holloszy JO. Effect of prolonged exercise on muscle citrate concentration before and after endurance training in men. Am J Physiol [Internet]. 1993;264:E215–20. Available from: http://www.ncbi.nlm.nih.gov/pubmed/8447387

58. Houmard JA, Shinebarger MH, Dolan PL, Leggett-Frazier N, Bruner RK, McCammon MR, et al. Exercise training increases GLUT-4 protein concentration in previously sedentary middle-aged men. Am J Physiol. 1993;264:896–901.

59. Esbjörnsson M, Jansson E, Sundberg CJ, Sylvén C, Eiken O, Nygren A, et al. Muscle fibre types and enzyme activities after training with local leg ischaemia in man. Acta Physiol Scand [Internet]. 1993;148:233–41. Available from: http://www.ncbi.nlm.nih.gov/pubmed/8213179

60. Young a J, Sawka MN, Quigley MD, Cadarette BS, Neufer PD, Dennis RC, et al. Role of thermal factors on aerobic capacity improvements with endurance training. J Appl Physiol. 1993;75:49–54.

61. Moore GE, Parsons DB, Stray-Gundersen J, Painter PL, Brinker KR, Mitchell JH. Uremic myopathy limits aerobic capacity in hemodialysis patients. Am J Kidney Dis [Internet]. National Kidney Foundation, Inc.; 1993;22:277–87. Available from: http://dx.doi.org/10.1016/S0272-6386(12)70319-0

62. Kiens B, Essen-Gustavsson B, Christensen NJ, Saltin B. Skeletal muscle substrate utilization during submaximal exercise in man: effect of endurance training. J Physiol. 1993;469:459–78.

63. Dela F, Handberg A, Mikines KJ, Vinten J, Galbo H. GLUT 4 and insulin receptor binding and kinase activity in trained human muscle. J Physiol [Internet]. 1993;469:615–24. Available from: http://www.ncbi.nlm.nih.gov/pubmed/8271219

64. Desplanches D, Hoppeler H, Linossier MT, Denis C, Claassen H, Dormois D, et al. Effects of training in normoxia and normobaric hypoxia on human muscle ultrastructure. Pflugers Arch [Internet]. 1993;425:263–7. Available from: http://www.ncbi.nlm.nih.gov/pubmed/8309787

65. Arenas J, Huertas R, Campos Y, Díaz AE, Villalón JM, Vilas E. Effects of L-carnitine on the pyruvate dehydrogenase complex and carnitine palmitoyl transferase activities in muscle of endurance athletes. FEBS Lett [Internet]. 1994;341:91–3. Available from: http://doi.wiley.com/10.1016/0014-5793%2894%2980246-7

66. Tremblay A, Simoneau JA, Bouchard C. Impact of exercise intensity on body fatness and skeletal muscle metabolism. Metabolism [Internet]. 1994;43:814–8. Available from: http://www.ncbi.nlm.nih.gov/pubmed/8028502

67. Green HJ, Jones S, Ball-Burnett M, Farrance B, Ranney D. Adaptations in muscle metabolism to prolonged voluntary exercise and training. J Appl Physiol. 1995;78:138–45.

68. Suter E, Hoppeler H, Claassen H, Billeter R, Aebi U, Horber F, et al. Ultrastructural modification of human skeletal muscle tissue with 6-month moderate-intensity exercise training. Int J Sport Med [Internet]. 1995;16:160–6. Available from: http://www.ncbi.nlm.nih.gov/entrez/query.fcgi?cmd=Retrieve&db=PubMed&dopt=Citation&list_uids=7649706

69. Hambrecht R, Niebauer J, Fiehn E, Kälberer B, Offner B, Hauer K, et al. Physical training in patients with stable chronic heart failure: Effects on cardiorespiratory fitness and ultrastructural abnormalities of leg muscles. J Am Coll Cardiol. 1995;25:1239–49.

70. Berthon P, Freyssenet D, Chatard JC, Castells J, Mujika I, Geyssant A, et al. Mitochondrial ATP production rate in 55 to 73-year-old men: effect of endurance training. Acta Physiol Scand [Internet]. 1995;154:269–74. Available from: http://www.ncbi.nlm.nih.gov/entrez/query.fcgi?cmd=Retrieve&db=PubMed&dopt=Citation&list_uids=7572222

71. Phillips SM, Green HJ, Tarnopolsky MA, Grant SM. Decreased glucose turnover after short-term training is unaccompanied by changes in muscle oxidative potential. Am J Physiol [Internet]. 1995;269:E222-30. Available from: http://www.ncbi.nlm.nih.gov/pubmed/7653539

72. Belardinelli R, Georgiou D, Scocco V, Barstow TJ, Purcaro A. Low intensity exercise training in patients with chronic heart failure. J Am Coll Cardiol. 1995;26:975–82.

73. Phillips SM, Green HJ, Macdonald MJ, Hughson RL. Progressive effect of endurance training at the onset of submaximal exercise on a vo. Am Physiol Soc. 1995;1914–20.

74. Ploutz-Snyder LL, Simoneau JA, Gilders RM, Staron RS, Hagerman FC. Cardiorespiratory and metabolic adaptations to hyperoxic training. Eur J Appl Physiol Occup Physiol [Internet]. 1996;73:38–48. Available from: http://www.ncbi.nlm.nih.gov/pubmed/8861667

75. Chesley A, Heigenhauser GJ, Spriet LL. Regulation of muscle glycogen phosphorylase activity following short-term endurance training. Am J Physiol [Internet]. 1996;270:E328-35. Available from: http://www.ncbi.nlm.nih.gov/pubmed/8779956

76. Gordon A, Tyni-Lenné R, Persson H, Kaijser L, Hultman E, Sylvén C. Markedly improved skeletal muscle function with local muscle training in patients with chronic heart failure. Clin Cardiol [Internet]. 1996;19:568–74. Available from: http://www.ncbi.nlm.nih.gov/pubmed/8818438

77. Maltais F, LeBlanc P, Simard C, Jobin J, Bérubé C, Bruneau J, et al. Skeletal muscle adaptation to endurance training in patients with chronic obstructive pulmonary disease. Am J Respir Crit Care Med [Internet]. 1996;154:442–7. Available from: http://www.ncbi.nlm.nih.gov/pubmed/8756820

78. Ades PA, Waldmann ML, Meyer WL, Brown KA, Poehlman ET, Pendlebury WW, et al. Skeletal muscle and cardiovascular adaptations to exercise conditioning in older coronary patients. Circulation [Internet]. 1996;94:323–30. Available from: http://www.ncbi.nlm.nih.gov/pubmed/8759072

79. Hiatt WR, Regensteiner JG, Wolfel EE, Carry MR, Brass EP. Effect of exercise training on skeletal muscle histology and metabolism in peripheral arterial disease. J Appl Physiol [Internet]. 1996;81:780–8. Available from: https://www.scopus.com/inward/record.uri?eid=2-s2.0-0029746681&partnerID=40&md5=80601423c53c762daea1ef6289ea5006

80. Tiidus P, Pushkarenko J, Houston M. Lack of antioxidant adaptation to short-term aerobic training in human muscle. Am J Physiol Regul Integr Comp Physiol [Internet]. 1996;271:832–6. Available from: http://www.ncbi.nlm.nih.gov/entrez/query.fcgi?cmd=Retrieve&db=PubMed&dopt=Citation&list_uids=8897971

81. Desplanches D, Hoppeler H, Tüscher L, Mayet MH, Spielvogel H, Ferretti G, et al. Muscle tissue adaptations of high-altitude natives to training in chronic hypoxia or acute normoxia. J Appl Physiol. 1996;81:1946–51.

82. Weston AR, Myburgh KH, Lindsay FH, Dennis SC, Noakes TD, Hawley JA. Skeletal muscle buffering capacity and endurance performance after high-intensity interval training by well-trained cyclists. Eur J Appl Physiol Occup Physiol [Internet]. 1997;75:7–13. Available from: http://www.ncbi.nlm.nih.gov/pubmed/9007451

83. Melissa L, MacDougall JD, Tarnopolsky MA, Cipriano N, Green HJ. Skeletal muscle adaptations to training under normobaric hypoxic versus normoxic conditions. Med Sci Sports Exerc [Internet]. 1997;29:238–43. Available from: http://www.ncbi.nlm.nih.gov/pubmed/9044229

84. Kiens B, Kristiansen S, Jensen P, Richter EA, Turcotte LP. Membrane associated fatty acid binding protein (FABPpm) in human skeletal muscle is increased by endurance training. Biochem Biophys Res Commun [Internet]. 1997;231:463–5. Available from: http://www.ncbi.nlm.nih.gov/entrez/query.fcgi?cmd=Retrieve&db=PubMed&dopt=Citation&list_uids=9070301%5Cnhttp://ac.els-cdn.com/S0006291X97961180/1-s2.0-S0006291X97961180-main.pdf?_tid=88b51efa-b3c4-11e3-811c-00000aab0f6c&acdnat=1395714424_a007e8ff9ef6c7583

85. Gordon A, Tyni-Lenné R, Jansson E, Kaijser L, Theodorsson-Norheim E, Sylvén C. Improved ventilation and decreased sympathetic stress in chronic heart failure patients following local endurance training with leg muscles. J Card Fail [Internet]. 1997;3:3–12. Available from: http://www.ncbi.nlm.nih.gov/pubmed/9110249

86. Margaritis I, Tessier F, Prou E, Marconnet P, Marini JF. Effects of endurance training on skeletal muscle oxidative capacities with and without selenium supplementation. J Trace Elem Med Biol [Internet]. Gustav Fischer Verlag; 1997;11:37–43. Available from: http://www.ncbi.nlm.nih.gov/pubmed/9176870

87. Helge JW, Kiens B. Muscle enzyme activity in humans: role of substrate availability and training. Am J Physiol. 1997;272:R1620–4.

88. Tyni-Lenné R, Gordon A, Jansson E, Bermann G, Sylvén C. Skeletal muscle endurance training improves peripheral oxidative capacity, exercise tolerance, and health-related quality of life in women with chronic congestive heart failure secondary to either ischemic cardiomyopathy or idiopathic dilated cardiomyopat. Am J Cardiol [Internet]. 1997;80:1025–9. Available from: http://www.ncbi.nlm.nih.gov/pubmed/9352972

89. Linossier MT, Dormois D, Perier C, Frey J, Geyssant A, Denis C. Enzyme adaptations of human skeletal muscle during bicycle short-sprint training and detraining. Acta Physiol Scand [Internet]. 1997;161:439–45. Available from: http://www.ncbi.nlm.nih.gov/pubmed/9429650

90. Turner DL, Hoppeler H, Claassen H, Vock P, Kayser B, Schena F, et al. Effects of endurance training on oxidative capacity and structural composition of human arm and leg muscles. Acta Physiol Scand [Internet]. 1997;161:459–64. Available from: http://www.ncbi.nlm.nih.gov/pubmed/9429652

91. Tyni-Lenné R, Gordon A, Europe E, Jansson E, Sylvén C. Exercise-based rehabilitation improves skeletal muscle capacity, exercise tolerance, and quality of life in both women and men with chronic heart failure. J Card Fail [Internet]. 1998;4:9–17. Available from: http://www.ncbi.nlm.nih.gov/pubmed/9573499

92. MacDougall JD, Hicks AL, MacDonald JR, McKelvie RS, Green HJ, Smith KM. Muscle performance and enzymatic adaptations to sprint interval training. J Appl Physiol [Internet]. 1998;84:2138–42. Available from: http://www.ncbi.nlm.nih.gov/pubmed/9609810

93. Putman CT, Jones NL, Hultman E, Hollidge-Horvat MG, Bonen A, McConachie DR, et al. Effects of short-term submaximal training in humans on muscle metabolism in exercise. Am J Physiol [Internet]. 1998;275:E132-9. Available from: http://www.ncbi.nlm.nih.gov/pubmed/9688884

94. Dawson B, Fitzsimons M, Green S, Goodman C, Carey M, Cole K. Changes in performance, muscle metabolites, enzymes

and fibre types after short sprint training. Eur J Appl Physiol Occup Physiol [Internet]. 1998;78:163–9. Available from: http://www.ncbi.nlm.nih.gov/pubmed/9694316

95. Lampert E, Mettauer B, Hoppeler H, Charloux A, Charpentier A, Lonsdorfer J. Skeletal muscle response to short endurance training in heart transplant recipients. Jacc. 1998;32:420–6.

96. Ferketich AK, Kirby TE, Alway SE. Cardiovascular and muscular adaptations to combined endurance and strength training in elderly women. Acta Physiol Scand. 1998;164:259–67.

97. Starritt EC, Angus D, Hargreaves M. Effect of short-term training on mitochondrial ATP production rate in human skeletal muscle. J Appl Physiol [Internet]. 1999;86:450–4. Available from: http://www.ncbi.nlm.nih.gov/pubmed/9931175

98. Green HJ, Dahly A, Shoemaker K, Goreham C, Bombardier E, Ball-Burnett M. Serial effects of high-resistance and prolonged endurance training on Na+-K+pump concentration and enzymatic activities in human vastus lateralis. Acta Physiol Scand. 1999;165:177–84.

99. Green H, MacDougall J, Tarnopolsky M, Melissa NL. Downregulation of Na+-K+-ATPase pumps in skeletal muscle with training in normobaric hypoxia. J Appl Physiol [Internet]. 1999;86:1745–8. Available from: http://www.ncbi.nlm.nih.gov/pubmed/10233143

100. Green H, Grant S, Bombardier E, Ranney D. Initial aerobic power does not alter muscle metabolic adaptations to short-term training. Am J Physiol. 1999;277:E39–48.

101. Greiwe JS, Hickner RC, Hansen PA, Racette SB, Chen MM, Holloszy JO. Effects of endurance exercise training on muscle glycogen accumulation in humans. J Appl Physiol [Internet]. 1999;87:222–6. Available from: http://jap.physiology.org/content/87/1/222%5Cnhttp://jap.physiology.org/content/87/1/222.long%5Cnhttp://jap.physiology.org/content/jap/87/1/222.full.pdf%5Cnhttp://www.ncbi.nlm.nih.gov/pubmed/10409578

102. Evertsen F, Medbø JI, Jebens E, Gjøvaag TF. Effect of training on the activity of five muscle enzymes studied on elite cross-country skiers. Acta Physiol Scand. 1999;167:247–57.

103. Dubouchaud H, Butterfield GE, Wolfel EE, Bergman BC, Brooks G a. Endurance training, expression, and physiology of LDH, MCT1, and MCT4 in human skeletal muscle. Am J Physiol Endocrinol Metab [Internet]. 2000;278:571–9. Available from: http://ajpendo.physiology.org/content/278/4/E571.short

104. McKenzie S, Phillips SM, Carter SL, Lowther S, Gibala MJ, Tarnopolsky M a. Endurance exercise training attenuates leucine oxidation and BCOAD activation during exercise in humans. Am J Physiol Endocrinol Metab. 2000;278:E580–7.

105. Parra J, Cadefau JA, Rodas G, Amigó N, Cussö R. The distribution of rest periods affects performance and adaptations of energy metabolism induced by high-intensity training in human muscle. Acta Physiol Scand. 2000;169:157–65.

106. Daugaard JR, Nielsen JN, Kristiansen S, Andersen JL, Hargreaves M, Richter EA. Fiber type - specific expression of GLUT4 in human skeletal muscle. Influence of exercise training. Diabetes. 2000;49:1092–5.

107. Horowitz JF, Leone TC, Feng W, Kelly DP, Klein S. Effect of endurance training on lipid metabolism in women: a potential role for PPARalpha in the metabolic response to training. Am J Physiol Endocrinol Metab [Internet]. 2000;279:E348-55. Available from: http://www.ncbi.nlm.nih.gov/pubmed/10913035

108. Frandsen U, Höffner L, Betak a, Saltin B, Bangsbo J, Hellsten Y. Endurance training does not alter the level of neuronal nitric oxide synthase in human skeletal muscle. J Appl Physiol [Internet]. 2000;89:1033–8. Available from: http://www.ncbi.nlm.nih.gov/pubmed/10956347

109. Rodas G, Ventura JL, Cadefau JA, Cussó R, Parra J. A short training programme for the rapid improvement of both aerobic and anaerobic metabolism. Eur J Appl Physiol [Internet]. 2000;82:480–6. Available from: http://www.ncbi.nlm.nih.gov/pubmed/10985604

110. Lange KH, Isaksson F, Juul A, Rasmussen MH, Bülow J, Kjaer M. Growth hormone enhances effects of endurance training on oxidative muscle metabolism in elderly women. Am J Physiol Endocrinol Metab [Internet]. 2000;279:E989-96. Available from: http://www.ncbi.nlm.nih.gov/pubmed/11052952

111. Helge JW, Wu BJ, Willer M, Daugaard JR, Storlien LH, Kiens B. Training affects muscle phospholipid fatty acid composition in humans. J Appl Physiol [Internet]. 2001;90:670–7. Available from: http://www.ncbi.nlm.nih.gov/pubmed/11160068

112. Youngren JF, Keen S, Kulp JL, Tanner CJ, Houmard J a, Goldfine ID. Enhanced muscle insulin receptor autophosphorylation with short-term aerobic exercise training. Am J Physiol Endocrinol Metab [Internet]. 2001;280:E528-33. Available from: http://www.ncbi.nlm.nih.gov/pubmed/11171609

113. Malenfant P, Tremblay a, Doucet E, Imbeault P, Simoneau J a, Joanisse DR. Elevated intramyocellular lipid concentration in obese subjects is not reduced after diet and exercise training. Am J Physiol Endocrinol Metab [Internet]. 2001;280:E632-9. Available from: http://www.ncbi.nlm.nih.gov/pubmed/11254471

114. Gustafsson T, Bodin K, Sylven C, Gordon A, Tyni-Lenne R, Jansson E. Increased expression of VEGF following exercise training in patients with heart failure. Eur J Clin Invest [Internet]. 2001;31:362–6. Available from: http://www.ncbi.nlm.nih.gov/pubmed/11298785

115. Jubrias S a, Esselman PC, Price LB, Cress ME, Conley KE. Large energetic adaptations of elderly muscle to resistance and endurance training. J Appl Physiol. 2001;90:1663–70.

116. Carter S, Rennie C, Hamilton S, Tarnopolsky M. Changes in skeletal muscle in males and females following endurance training. Can J Physiol Pharmacol. 2001;79:386–92.

117. Taivassalo T, Shoubridge EA, Chen J, Kennaway NG, DiMauro S, Arnold DL, et al. Aerobic conditioning in patients with mitochondrial myopathies: physiological, biochemical, and genetic effects. Ann Neurol [Internet]. 2001;50:133–41. Available from: http://www.ncbi.nlm.nih.gov/pubmed/11506394

118. Costes F, Prieur F, Féasson L, Geyssant a, Barthélémy JC, Denis C. Influence of training on NIRS muscle oxygen saturation during submaximal exercise. Med Sci Sports Exerc. 2001;33:1484–9.

119. Bell C, Paterson DH, Kowalchuk JM, Moy a P, Thorp DB, Noble EG, et al. Determinants of oxygen uptake kinetics in older humans following single-limb endurance exercise training. Exp Physiol. 2001;86:659–65.

120. Masuda K, Okazaki K, Kuno S, Asano K, Shimojo H, Katsuta S. Endurance training under 2500-m hypoxia does not increase myoglobin content in human skeletal muscle. Eur J Appl Physiol [Internet]. 2001;85:486–90. Available from: http://www.ncbi.nlm.nih.gov/pubmed/11606019

121. Bengtsson J, Gustafsson T, Widegren U, Jansson E, Sundberg CJ. Mitochondrial transcription factor A and respiratory complex IV increase in response to exercise training in humans. Pflugers Arch Eur J Physiol. 2001;443:61–6.

122. Geiser J, Vogt M, Billeter R, Zuleger C, Belforti F, Hoppeler H. Training high - living low: changes of aerobic performance and muscle structure with training at simulated altitude. / Entrainement en altitude et vie en basse altitude: evolution des performances aerobiques et de la structure des muscles avec un entraine. Int J Sports Med [Internet]. 2001;22:579–85. Available from: http://articles.sirc.ca/search.cfm?id=S-833340%5Cnhttp://ezproxy.library.yorku.ca/login?url=http://search.ebscohost.com/login.aspx?direct=true&db=sph&AN=SPHS-833340&site=ehost-live%5Cnhttp://www.thieme.com

123. Murakami H, Ota A, Simojo H, Okada M, Ajisaka R, Kuno S. Polymorphisms in control region of mtDNA relates to individual differences in endurance capacity or trainability. Jpn J Physiol [Internet]. 2002;52:247–56. Available from: http://www.ncbi.nlm.nih.gov/pubmed/12230801

124. Gustafsson T, Knutsson A, Puntschart A, Kaijser L, Nordqvist SAC, Sundberg C, et al. Increased expression of vascular endothelial growth factor in human skeletal muscle in response to short-term one-legged exercise training. Pflugers Arch Eur J Physiol. 2002;444:752–9.

125. Puente-Maestu L, Tena T, Trascasa C, Pérez-Parra J, Godoy R, García MJ, et al. Training improves muscle oxidative capacity and oxygenation recovery kinetics in patients with chronic obstructive pulmonary disease. Eur J Appl Physiol. 2003;88:580–7.

126. Pilegaard H, Saltin B, Neufer PD. Exercise induces transient transcriptional activation of the PGC-1α gene in human skeletal muscle. J Physiol [Internet]. 2003;546:851–8. Available from: http://doi.wiley.com/10.1113/jphysiol.2002.034850

127. Keteyian SJ, Duscha BD, Brawner CA, Green HJ, Marks CRC, Schachat FH, et al. Differential effects of exercise training in men and women with chronic heart failure. Am Heart J. 2003;145:912–8.

128. Charifi N, Kadi F, Féasson L, Denis C. Effects of endurance training on satellite cell frequency in skeletal muscle of old men. Muscle Nerve [Internet]. 2003;28:87–92. Available from: http://onlinelibrary.wiley.com/doi/10.1002/mus.10394/abstract%5Cnhttp://onlinelibrary.wiley.com/doi/10.1002/mus.10394/pdf

129. Zoll J, N'Guessan B, Ribera F, Lampert E, Fortin D, Veksler V, et al. Preserved response of mitochondrial function to short-term endurance training in skeletal muscle of heart transplant recipients. J Am Coll Cardiol. 2003;42:126–32.

130. Short KR, Vittone JL, Bigelow ML, Proctor DN, Rizza RA, Coenen-Schimke JM, et al. Impact of aerobic exercise training on age-related changes in insulin sensitivity and muscle oxidative capacity. Diabetes [Internet]. 2003;52:1888–96. Available from: citeulike-article-id:12641907%5Cnhttp://dx.doi.org/10.2337/diabetes.52.8.1888

131. Fernström M, Tonkonogi M, Sahlin K. Effects of acute and chronic endurance exercise on mitochondrial uncoupling in human skeletal muscle. J Physiol. 2004;554:755–63.

132. Bruce CR, Kriketos AD, Cooney GJ, Hawley JA. Disassociation of muscle triglyceride content and insulin sensitivity after exercise training in patients with Type 2 diabetes. Diabetologia. 2004;47:23–30.

133. LeBlanc PJ, Peters SJ, Tunstall RJ, Cameron-Smith D, Heigenhauser GJF. Effects of aerobic training on pyruvate dehydrogenase and pyruvate dehydrogenase kinase in human skeletal muscle. J Physiol. 2004;557:559–70.

134. Howarth KR, LeBlanc PJ, Heigenhauser GJF, Gibala MJ. Effect of endurance training on muscle TCA cycle metabolism during exercise in humans. J Appl Physiol [Internet]. 2004;97:579–84. Available from: http://jap.physiology.org/content/97/2/579.abstract

135. He J, Goodpaster BH, Kelley DE. Effects of Weight Loss and Physical Activity on Muscle Lipid Content and Droplet Size. Obes Res [Internet]. 2004;12:761–9. Available from: http://doi.wiley.com/10.1038/oby.2004.92

136. Kraniou GN, Cameron-Smith D, Hargreaves M. Effect of short-term training on GLUT-4 mRNA and protein expression in human skeletal muscle. Exp Physiol [Internet]. 2004;89:559–63. Available from: http://www.ncbi.nlm.nih.gov/pubmed/15184360

137. Stewart BG, Tarnopolsky MA, Hicks AL, McCartney N, Mahoney DJ, Staron R, et al. Treadmill training-induced adaptations in muscle phenotype in persons with incomplete spinal cord injury. Muscle and Nerve. 2004;30:61–8.

138. Pruchnic R, Katsiaras A, He J, Kelley DE, Winters C, Goodpaster BH. Exercise training increases intramyocellular lipid and oxidative capacity in older adults. Am J Physiol Endocrinol Metab. 2004;287:E857-62.

139. Fischer CP, Plomgaard P, Hansen AK, Pilegaard H, Saltin B, Pedersen BK. Endurance training reduces the contraction-induced interleukin-6 mRNA expression in human skeletal muscle. Am J Physiol Endocrinol Metab. 2004;287:1189–94.

140. Hansen AK, Fischer CP, Plomgaard P, Andersen JL, Saltin B, Pedersen BK. Skeletal muscle adaptation: training twice every second day vs. training once daily. J Appl Physiol. 2005;98:93–9.

141. Kim HJ, Lee JS, Kim CK. Effect of exercise training on muscle glucose transporter 4 protein and intramuscular lipid content in elderly men with impaired glucose tolerance. Eur J Appl Physiol. 2004;93:353–8.

142. Barnett C, Carey M, Proietto J, Cerin E, Febbraio MA, Jenkins D. Muscle metabolism during sprint exercise in man: Influence of sprint training. J Sci Med Sport. 2004;7:314–22.

143. Menshikova E V, Ritov VB, Toledo FG, Ferrell RE, Goodpaster BH, Kelley DE. Effects of weight loss and physical activity on skeletal muscle mitochondrial function in obesity. AmJPhysiol EndocrinolMetab. 2005;288:E818–25.

144. Burgomaster KA, Hughes SC, Heigenhauser GJF, Bradwell SN, Gibala MJ. Six sessions of sprint interval training increases muscle oxidative potential and cycle endurance capacity in humans. J Appl Physiol [Internet]. 2005;98:1985–90. Available from: http://www.ncbi.nlm.nih.gov/pubmed/15705728

145. Messonnier L, Denis C, Prieur F, Lacour JR. Are the effects of training on fat metabolism involved in the improvement of performance during high-intensity exercise? Eur J Appl Physiol. 2005;94:434–41.

146. Gielen S, Adams V, Linke A, Erbs S, Möbius-Winkler S, Schubert A, et al. Exercise training in chronic heart failure: correlation between reduced local inflammation and improved oxidative capacity in the skeletal muscle. Eur J Cardiovasc Prev {&} Rehabil [Internet]. 2005;12:393–400. Available from: http://eutils.ncbi.nlm.nih.gov/entrez/eutils/elink.fcgi?dbfrom=pubmed&id=16079649&retmode=ref&cmd=prlinks

147. Zoll J, Steiner R, Meyer K, Vogt M, Hoppeler H, Flück M. Gene expression in skeletal muscle of coronary artery disease patients after concentric and eccentric endurance training. Eur J Appl Physiol. 2006;96:413–22.

148. Gosker HR, Schrauwen P, Broekhuizen R, Hesselink MKC, Moonen-Kornips E, Ward KA, et al. Exercise training restores uncoupling protein-3 content in limb muscles of patients with chronic obstructive pulmonary disease. Am J Physiol Endocrinol Metab [Internet]. 2006;290:E976-81. Available from: http://www.ncbi.nlm.nih.gov/pubmed/16352674

149. Østergård T, Andersen JL, Nyholm B, Lund S, Nair KS, Saltin B, et al. Impact of exercise training on insulin sensitivity, physical fitness, and muscle oxidative capacity in first-degree relatives of type 2 diabetic patients. Am J Physiol Endocrinol Metab [Internet]. 2006;290:E998-1005. Available from: http://www.ncbi.nlm.nih.gov/pubmed/16352678

150. Vogiatzis I, Terzis G, Nanas S, Stratakos G, Simoes DCM, Georgiadou O, et al. Skeletal muscle adaptations to interval training in patients with advanced COPD. Chest [Internet]. The American College of Chest Physicians; 2005;128:3838–45. Available from: http://dx.doi.org/10.1378/chest.128.6.3838

151. Messonnier L, Freund H, Denis C, F??asson L, Lacour JR. Effects of training on lactate kinetics parameters and their

influence on short high-intensity exercise performance. Int J Sports Med. 2006;27:60–6.

152. Helge JW, Overgaard K, Damsgaard R, S??rensen K, Andersen JL, Dyrskog SEU, et al. Repeated prolonged whole-body low-intensity exercise: Effects on insulin sensitivity and limb muscle adaptations. Metabolism. 2006;55:217–23.

153. Bruce CR, Thrush AB, Mertz VA, Bezaire V, Chabowski A, Heigenhauser GJF, et al. Endurance training in obese humans improves glucose tolerance and mitochondrial fatty acid oxidation and alters muscle lipid content. Am J Physiol Endocrinol Metab. 2010;291:99–107.

154. Burgomaster K a, Heigenhauser GJF, Gibala MJ, Kirsten A. Effect of short-term sprint interval training on human skeletal muscle carbohydrate metabolism during exercise and time-trial performance. J Appl Physiol. 2006;1:2041–7.

155. Zoll J. Exercise training in normobaric hypoxia in endurance runners. III. Muscular adjustments of selected gene transcripts. J Appl Physiol [Internet]. 2005;100:1258–66. Available from: http://jap.physiology.org/cgi/doi/10.1152/japplphysiol.00359.2005

156. Haller RG, Wyrick P, Taivassalo T, Vissing J. Aerobic conditioning: An effective therapy in McArdle's disease. Ann Neurol. 2006;59:922–8.

157. Menshikova E V, Ritov VB, Fairfull L, Ferrell RE, Kelley DE, Goodpaster BH. Effects of exercise on mitochondrial content and function in aging human skeletal muscle. J Gerontol A Biol Sci Med Sci [Internet]. 2006;61:534–40. Available from: http://www.ncbi.nlm.nih.gov/pubmed/16799133

158. Jeppesen TD, Schwartz M, Olsen DB, Wibrand F, Krag T, Dunø M, et al. Aerobic training is safe and improves exercise capacity in patients with mitochondrial myopathy. Brain. 2006;129:3402–12.

159. Gibala MJ, Little JP, van Essen M, Wilkin GP, Burgomaster K a, Safdar A, et al. Short-term sprint interval versus traditional endurance training: similar initial adaptations in human skeletal muscle and exercise performance. J Physiol. 2006;575:901–11.

160. Roels B, Thomas C, Bentley DJ, Mercier J, Hayot M, Millet G. Effects of intermittent hypoxic training on amino and fatty acid oxidative combustion in human permeabilized muscle fibers. J Appl Physiol. 2007;102:79–86.

161. Lee JK, Lee JS, Park H, Cha Y-S, Yoon CS, Kim CK. Effect of L-carnitine supplementation and aerobic training on FABPc content and beta-HAD activity in human skeletal muscle. Eur J Appl Physiol [Internet]. 2007;99:193–9. Available from: http://www.ncbi.nlm.nih.gov/pubmed/17089153

162. Tarnopolsky MA, Rennie CD, Robertshaw HA, Fedak-Tarnopolsky SN, Devries MC, Hamadeh MJ. Influence of endurance exercise training and sex on intramyocellular lipid and mitochondrial ultrastructure, substrate use, and mitochondrial enzyme activity. AJP Regul Integr Comp Physiol [Internet]. 2006;292:R1271–8. Available from: http://ajpregu.physiology.org/cgi/doi/10.1152/ajpregu.00472.2006

163. Perry CGR, Talanian JL, Heigenhauser GJF, Spriet LL. The effects of training in hyperoxia vs. normoxia on skeletal muscle enzyme activities and exercise performance. J Appl Physiol [Internet]. 2006;102:1022–7. Available from: http://jap.physiology.org/cgi/doi/10.1152/japplphysiol.01215.2006

164. Talanian JL, Galloway SDR, Heigenhauser GJF, Bonen A, Spriet LL. Two weeks of high-intensity aerobic interval training increases the capacity for fat oxidation during exercise in women. J Appl Physiol. 2007;102:1439–47.

165. Heilbronn LK, Seng KG, Turner N, Campbell L V., Chisholm DJ. Markers of mitochondrial biogenesis and metabolism are lower in overweight and obese insulin-resistant subjects. J Clin Endocrinol Metab. 2007;92:1467–73.

166. Burgomaster KA, Cermak NM, Phillips SM, Benton CR, Bonen A, Gibala MJ. Divergent response of metabolite transport proteins in human skeletal muscle after sprint interval training and detraining. Am J Physiol Regul Integr Comp Physiol [Internet]. 2007;292:R1970-6. Available from: http://www.ncbi.nlm.nih.gov/pubmed/17303684

167. Menshikova E V, Ritov VB, Ferrell RE, Azuma K, Goodpaster BH, Kelley DE. Characteristics of skeletal muscle mitochondrial biogenesis induced by moderate-intensity exercise and weight loss in obesity. J Appl Physiol [Internet]. 2007;103:21–7. Available from: http://www.ncbi.nlm.nih.gov/pubmed/17332268

168. Helge JW, Damsgaard R, Overgaard K, Andersen JL, Donsmark M, Dyrskog SE, et al. Low-intensity training dissociates metabolic from aerobic fitness. Scand J Med Sci Sports [Internet]. 2008;18:86–94. Available from: http://www.ncbi.nlm.nih.gov/pubmed/17355324

169. Bakkman L, Sahlin K, Holmberg HC, Tonkonogi M. Quantitative and qualitative adaptation of human skeletal muscle mitochondria to hypoxic compared with normoxic training at the same relative work rate. Acta Physiol. 2007;190:243–51.

170. Helge JW, Bentley D, Schjerling P, Willer M, Gibala MJ, Franch J, et al. Four weeks one-leg training and high fat diet

does not alter PPARα protein or mRNA expression in human skeletal muscle. Eur J Appl Physiol. 2007;101:105–14.

171. Toledo FGS, Menshikova E V, Ritov VB, Azuma K, Radikova Z, DeLany J, et al. Effects of Physical Activity and Weight Loss on Skeletal Muscle Mitochondria and Relationship With Glucose Control in Type 2 Diabetes. Diabetes [Internet]. 2007;56:2142–7. Available from: http://diabetes.diabetesjournals.org/cgi/doi/10.2337/db07-0141%5Cnpapers3://publication/doi/10.2337/db07-0141

172. Adhihetty PJ, Taivassalo T, Haller RG, Walkinshaw DR, Hood DA. The effect of training on the expression of mitochondrial biogenesis- and apoptosis-related proteins in skeletal muscle of patients with mtDNA defects. AJP Endocrinol Metab [Internet]. 2007;293:E672–80. Available from: http://ajpendo.physiology.org/cgi/doi/10.1152/ajpendo.00043.2007

173. Burgomaster KA, Howarth KR, Phillips SM, Rakobowchuk M, Macdonald MJ, McGee SL, et al. Similar metabolic adaptations during exercise after low volume sprint interval and traditional endurance training in humans. J Physiol [Internet]. 2008;586:151–60. Available from: http://www.ncbi.nlm.nih.gov/pubmed/17991697

174. Toledo FGS, Menshikova E V, Azuma K, Radiková Z, Kelley CA, Ritov VB, et al. Mitochondrial capacity in skeletal muscle is not stimulated by weight loss despite increases in insulin action and decreases in intramyocellular lipid content. Diabetes [Internet]. 2008;57:987–94. Available from: http://www.ncbi.nlm.nih.gov/pubmed/18252894

175. Dubé JJ, Amati F, Stefanovic-Racic M, Toledo FGS, Sauers SE, Goodpaster BH. Exercise-induced alterations in intramyocellular lipids and insulin resistance: the athlete's paradox revisited. Am J Physiol Endocrinol Metab [Internet]. 2008;294:E882-8. Available from: http://www.ncbi.nlm.nih.gov/pubmed/18319352

176. Bordenave S, Metz L, Flavier S, Lambert K, Ghanassia E, Dupuy A-M, et al. Training-induced improvement in lipid oxidation in type 2 diabetes mellitus is related to alterations in muscle mitochondrial activity. Effect of endurance training in type 2 diabetes. Diabetes Metab [Internet]. 2008;34:162–8. Available from: http://www.ncbi.nlm.nih.gov/pubmed/18396088

177. Green HJ, Duhamel TA, Stewart RD, Tupling AR, Ouyang J. Dissociation between changes in muscle Na+-K+-ATPase isoform abundance and activity with consecutive days of exercise and recovery. Am J Physiol Endocrinol Metab [Internet]. 2008;294:E761-7. Available from: http://www.ncbi.nlm.nih.gov/pubmed/18230697

178. Harmer AR, Chisholm DJ, McKenna MJ, Hunter SK, Ruell PA, Naylor JM, et al. Sprint training increases muscle oxidative metabolism during high-intensity exercise in patients with type 1 diabetes. Diabetes Care [Internet]. 2008;31:2097–102. Available from: http://www.ncbi.nlm.nih.gov/pubmed/18716051

179. Yeo WK, Paton CD, Garnham AP, Burke LM, Carey AL, Hawley JA. Skeletal muscle adaptation and performance responses to once a day versus twice every second day endurance training regimens. J Appl Physiol [Internet]. 2008;105:1462–70. Available from: http://www.ncbi.nlm.nih.gov/pubmed/18772325

180. Iaia FM, Hellsten Y, Nielsen JJ, Fernstrom M, Sahlin K, Bangsbo J. Four weeks of speed endurance training reduces energy expenditure during exercise and maintains muscle oxidative capacity despite a reduction in training volume. J Appl Physiol [Internet]. 2008;106:73–80. Available from: http://jap.physiology.org/cgi/doi/10.1152/japplphysiol.90676.2008

181. Perry CGR, Heigenhauser GJF, Bonen A, Spriet LL. High-intensity aerobic interval training increases fat and carbohydrate metabolic capacities in human skeletal muscle. Appl Physiol Nutr Metab [Internet]. 2008;33:1112–23. Available from: http://www.nrcresearchpress.com/doi/abs/10.1139/H08-097

182. Preisler N, Andersen G, Thøgersen F, Crone C, Jeppesen TD, Wibrand F, et al. Effect of aerobic training in patients with spinal and bulbar muscular atrophy (Kennedy disease). Neurology [Internet]. 2009;72:317–23. Available from: http://www.ncbi.nlm.nih.gov/pubmed/19171827

183. Vollaard NBJ, Constantin-Teodosiu D, Fredriksson K, Rooyackers O, Jansson E, Greenhaff PL, et al. Systematic analysis of adaptations in aerobic capacity and submaximal energy metabolism provides a unique insight into determinants of human aerobic performance. J Appl Physiol [Internet]. 2009;106:1479–86. Available from: http://www.ncbi.nlm.nih.gov/pubmed/19196912

184. Bajpeyi S, Tanner CJ, Slentz CA, Duscha BD, McCartney JS, Hickner RC, et al. Effect of exercise intensity and volume on persistence of insulin sensitivity during training cessation. J Appl Physiol [Internet]. 2009;106:1079–85. Available from: http://www.ncbi.nlm.nih.gov/pubmed/19196913

185. Akerstrom TCA, Fischer CP, Plomgaard P, Thomsen C, van Hall G, Pedersen BK. Glucose ingestion during endurance training does not alter adaptation. J Appl Physiol [Internet]. 2009;106:1771–9. Available from: http://www.ncbi.nlm.nih.gov/pubmed/19228984

186. Morton JP, Croft L, Bartlett JD, Maclaren DPM, Reilly T, Evans L, et al. Reduced carbohydrate availability does not modulate training-induced heat shock protein adaptations but does upregulate oxidative enzyme activity in human skeletal muscle. J Appl Physiol [Internet]. 2009;106:1513–21. Available from: http://www.ncbi.nlm.nih.gov/pubmed/19265068

187. Hansen D, Dendale P, Jonkers RAM, Beelen M, Manders RJF, Corluy L, et al. Continuous low- to moderate-intensity exercise training is as effective as moderate- to high-intensity exercise training at lowering blood HbA(1c) in obese type 2 diabetes patients. Diabetologia [Internet]. 2009;52:1789–97. Available from: http://www.ncbi.nlm.nih.gov/pubmed/19370339

188. Nybo L, Pedersen K, Christensen B, Aagaard P, Brandt N, Kiens B. Impact of carbohydrate supplementation during endurance training on glycogen storage and performance. Acta Physiol (Oxf) [Internet]. 2009;197:117–27. Available from: http://www.ncbi.nlm.nih.gov/pubmed/19432594

189. Mogensen M, Vind BF, Højlund K, Beck-Nielsen H, Sahlin K. Maximal lipid oxidation in patients with type 2 diabetes is normal and shows an adequate increase in response to aerobic training. Diabetes Obes Metab [Internet]. 2009;11:874–83. Available from: http://www.ncbi.nlm.nih.gov/pubmed/19531056

190. Green HJ, Bombardier E, Burnett ME, Smith IC, Tupling SM, Ranney DA. Time-dependent effects of short-term training on muscle metabolism during the early phase of exercise. Am J Physiol Regul Integr Comp Physiol [Internet]. 2009;297:R1383-91. Available from: http://www.ncbi.nlm.nih.gov/pubmed/19710384

191. Bangsbo J, Gunnarsson TP, Wendell J, Nybo L, Thomassen M. Reduced volume and increased training intensity elevate muscle Na+-K+ pump alpha2-subunit expression as well as short- and long-term work capacity in humans. J Appl Physiol [Internet]. 2009;107:1771–80. Available from: http://www.ncbi.nlm.nih.gov/pubmed/19797693

192. Bangsbo J, Nielsen JJ, Mohr M, Randers MB, Krustrup BR, Brito J, et al. Performance enhancements and muscular adaptations of a 16-week recreational football intervention for untrained women. Scand J Med Sci Sports [Internet]. 2010;20 Suppl 1:24–30. Available from: http://www.ncbi.nlm.nih.gov/pubmed/19954496

193. YFANTI C, ÅKERSTRÖM T, NIELSEN S, NIELSEN AR, MOUNIER R, MORTENSEN OH, et al. Antioxidant Supplementation Does Not Alter Endurance Training Adaptation. Med Sci Sport Exerc [Internet]. 2010;42:1388–95. Available from: http://content.wkhealth.com/linkback/openurl?sid=WKPTLP:landingpage&an=00005768-201007000-00020

194. Nielsen J, Mogensen M, Vind BF, Sahlin K, Hojlund K, Schroder HD, et al. Increased subsarcolemmal lipids in type 2 diabetes: effect of training on localization of lipids, mitochondria, and glycogen in sedentary human skeletal muscle. AJP Endocrinol Metab [Internet]. 2010;298:E706–13. Available from: http://ajpendo.physiology.org/cgi/doi/10.1152/ajpendo.00692.2009

195. Krustrup P, Christensen JF, Randers MB, Pedersen H, Sundstrup E, Jakobsen MD, et al. Muscle adaptations and performance enhancements of soccer training for untrained men. Eur J Appl Physiol. 2010;108:1247–58.

196. Camera DM, Anderson MJ, Hawley JA, Carey AL. Short-term endurance training does not alter the oxidative capacity of human subcutaneous adipose tissue. Eur J Appl Physiol. 2010;109:307–16.

197. Little JP, Safdar A, Wilkin GP, Tarnopolsky M a, Gibala MJ. A practical model of low-volume high-intensity interval training induces mitochondrial biogenesis in human skeletal muscle: potential mechanisms. J Physiol. 2010;588:1011–22.

198. Schmutz S, Däpp C, Wittwer M, Durieux A-C, Mueller M, Weinstein F, et al. A hypoxia complement differentiates the muscle response to endurance exercise. Exp Physiol. 2010;95:723–35.

199. Hulston CJ, Venables MC, Mann CH, Martin C, Philp A, Baar K, et al. Training with low muscle glycogen enhances fat metabolism in well-trained cyclists. Med Sci Sports Exerc [Internet]. 2010;42:2046–55. Available from: http://www.ncbi.nlm.nih.gov/pubmed/20351596

200. Stannard SR, Buckley AJ, Edge JA, Thompson MW. Adaptations to skeletal muscle with endurance exercise training in the acutely fed versus overnight-fasted state. J Sci Med Sport [Internet]. Sports Medicine Australia; 2010;13:465–9. Available from: http://dx.doi.org/10.1016/j.jsams.2010.03.002

201. Cox GR, Clark SA, Cox AJ, Halson SL, Hargreaves M, Hawley JA, et al. Daily training with high carbohydrate availability increases exogenous carbohydrate oxidation during endurance cycling. J Appl Physiol [Internet]. 2010;109:126–34. Available from: http://www.ncbi.nlm.nih.gov/pubmed/20466803

202. Talanian JL, Holloway GP, Snook LA, Heigenhauser GJF, Bonen A, Spriet LL. Exercise training increases sarcolemmal and mitochondrial fatty acid transport proteins in human skeletal muscle. Am J Physiol Endocrinol Metab [Internet]. 2010;299:E180-8. Available from: http://www.ncbi.nlm.nih.gov/pubmed/20484014

203. Kohn TA, Essén-Gustavsson B, Myburgh KH. Specific muscle adaptations in type II fibers after high-intensity interval training of well-trained runners. Scand J Med Sci Sports [Internet]. 2011;21:765–72. Available from: http://www.ncbi.nlm.nih.gov/pubmed/20492589

204. Hey-Mogensen M, Højlund K, Vind BF, Wang L, Dela F, Beck-Nielsen H, et al. Effect of physical training on mitochondrial respiration and reactive oxygen species release in skeletal muscle in patients with obesity and type 2

diabetes. Diabetologia. 2010;53:1976–85.

205. Gurd BJ, Perry CGR, Heigenhauser GJF, Spriet LL, Bonen A. High-intensity interval training increases SIRT1 activity in human skeletal muscle. Appl Physiol Nutr Metab [Internet]. 2010;35:350–7. Available from: http://www.nrcresearchpress.com/doi/10.1139/H10-030

206. Konopka AR, Douglass MD, Kaminsky LA, Jemiolo B, Trappe TA, Trappe S, et al. Molecular adaptations to aerobic exercise training in skeletal muscle of older women. J Gerontol A Biol Sci Med Sci [Internet]. 2010;65:1201–7. Available from: http://www.ncbi.nlm.nih.gov/pubmed/20566734

207. Perry CGR, Lally J, Holloway GP, Heigenhauser GJF, Bonen A, Spriet LL. Repeated transient mRNA bursts precede increases in transcriptional and mitochondrial proteins during training in human skeletal muscle. J Physiol [Internet]. 2010;588:4795–810. Available from: http://www.ncbi.nlm.nih.gov/pubmed/20921196

208. Van Proeyen K, Szlufcik K, Nielens H, Ramaekers M, Hespel P. Beneficial metabolic adaptations due to endurance exercise training in the fasted state. J Appl Physiol [Internet]. 2011;110:236–45. Available from: http://www.ncbi.nlm.nih.gov/pubmed/21051570

209. Christensen PM, Krustrup P, Gunnarsson TP, Kiilerich K, Nybo L, Bangsbo J. VO2 kinetics and performance in soccer players after intense training and inactivity. Med Sci Sports Exerc [Internet]. 2011;43:1716–24. Available from: http://www.ncbi.nlm.nih.gov/pubmed/21311360

210. Abbiss CR, Karagounis LG, Laursen PB, Peiffer JJ, Martin DT, Hawley JA, et al. Single-leg cycle training is superior to double-leg cycling in improving the oxidative potential and metabolic profile of trained skeletal muscle. J Appl Physiol. 2011;110:1248–55.

211. McPhee JS, Perez-Schindler J, Degens H, Tomlinson D, Hennis P, Baar K, et al. HIF1A P582S gene association with endurance training responses in young women. Eur J Appl Physiol [Internet]. 2011;111:2339–47. Available from: http://www.ncbi.nlm.nih.gov/pubmed/21344271

212. Hood MS, Little JP, Tarnopolsky MA, Myslik F, Gibala MJ. Low-volume interval training improves muscle oxidative capacity in sedentary adults. Med Sci Sports Exerc. 2011;43:1849–56.

213. Luden N, Hayes E, Minchev K, Louis E, Raue U, Conley T, et al. Skeletal muscle plasticity with marathon training in novice runners. Scand J Med Sci Sport. 2012;22:662–70.

214. Irving BA, Short KR, Nair KS, Stump CS. Nine days of intensive exercise training improves mitochondrial function but not insulin action in adult offspring of mothers with type 2 diabetes (Journal of Clinical Endocrinology and Metabolism (2011) 96, (E1137-E1141)). J Clin Endocrinol Metab. 2011;96:2936.

215. McPhee JS, Williams AG, Perez-Schindler J, Degens H, Baar K, Jones DA. Variability in the magnitude of response of metabolic enzymes reveals patterns of co-ordinated expression following endurance training in women. Exp Physiol. 2011;96:699–707.

216. Blomstrand E, Krustrup P, Søndergaard H, Rådegran G, Calbet JAL, Saltin B. Exercise training induces similar elevations in the activity of oxoglutarate dehydrogenase and peak oxygen uptake in the human quadriceps muscle. Pflugers Arch Eur J Physiol. 2011;462:257–65.

217. Murias JM, Kowalchuk JM, Ritchie D, Hepple RT, Doherty TJ, Paterson DH. Adaptations in capillarization and citrate synthase activity in response to endurance training in older and young men. J Gerontol A Biol Sci Med Sci [Internet]. 2011;66:957–64. Available from: http://www.ncbi.nlm.nih.gov/pubmed/21715648

218. Slivka DR, Dumke CL, Hailes WS, Cuddy JS, Ruby BC. Substrate use and biochemical response to a 3,211-km bicycle tour in trained cyclists. Eur J Appl Physiol [Internet]. 2012;112:1621–30. Available from: http://www.ncbi.nlm.nih.gov/pubmed/21866362

219. Little JP, Gillen JB, Percival ME, Safdar A, Tarnopolsky MA, Punthakee Z, et al. Low-volume high-intensity interval training reduces hyperglycemia and increases muscle mitochondrial capacity in patients with type 2 diabetes. J Appl Physiol [Internet]. 2011;111:1554–60. Available from: http://www.ncbi.nlm.nih.gov/pubmed/21868679

220. Duscha BD, Robbins JL, Jones WS, Kraus WE, Lye RJ, Sanders JM, et al. Angiogenesis in skeletal muscle precede improvements in peak oxygen uptake in peripheral artery disease patients. Arterioscler Thromb Vasc Biol [Internet]. 2011;31:2742–8. Available from: http://www.ncbi.nlm.nih.gov/pubmed/21868709

221. Esposito F, Reese V, Shabetai R, Wagner PD, Richardson RS. Isolated quadriceps training increases maximal exercise capacity in chronic heart failure: The role of skeletal muscle convective and diffusive oxygen transport. J Am Coll Cardiol [Internet]. Elsevier Inc.; 2011;58:1353–62. Available from: http://dx.doi.org/10.1016/j.jacc.2011.06.025

222. Ngo KTA, Denis C, Saafi MA, Feasson L, Verney J. Endurance but not resistance training increases intra-myocellular lipid content and β-hydroxyacyl coenzyme A dehydrogenase activity in active elderly men. Acta Physiol (Oxf) [Internet]. 2012;205:133–44. Available from: http://www.ncbi.nlm.nih.gov/pubmed/22017921

223. Rud B, Foss O, Krustrup P, Secher NH, Hallén J. One-legged endurance training: leg blood flow and oxygen extraction during cycling exercise. Acta Physiol (Oxf) [Internet]. 2012;205:177–85. Available from: http://www.ncbi.nlm.nih.gov/pubmed/22059600

224. Serpiello FR, McKenna MJ, Bishop DJ, Aughey RJ, Caldow MK, Cameron-Smith D, et al. Repeated sprints alter signaling related to mitochondrial biogenesis in humans. Med Sci Sports Exerc [Internet]. 2012;44:827–34. Available from: http://www.ncbi.nlm.nih.gov/pubmed/23059859

225. Jeppesen J, Jordy AB, Sjøberg KA, Füllekrug J, Stahl A, Nybo L, et al. Enhanced fatty acid oxidation and FATP4 protein expression after endurance exercise training in human skeletal muscle. PLoS One. 2012;7:4–12.

226. Hutchison SK, Teede HJ, Rachoń D, Harrison CL, Strauss BJ, Stepto NK. Effect of exercise training on insulin sensitivity, mitochondria and computed tomography muscle attenuation in overweight women with and without polycystic ovary syndrome. Diabetologia. 2012;55:1424–34.

227. Duscha BD, Annex BH, Johnson JL, Huffman K, Houmard J, Kraus WE. Exercise dose response in muscle. Int J Sports Med. 2012;33:218–23.

228. Brønstad E, Rognmo O, Tjonna AE, Dedichen HH, Kirkeby-Garstad I, Håberg AK, et al. High-intensity knee extensor training restores skeletal muscle function in COPD patients. Eur Respir J. 2012;40:1130–6.

229. Robach P, Siebenmann C, Jacobs RA, Rasmussen P, Nordsborg N, Pesta D, et al. The role of haemoglobin mass on VO2max following normobaric “live high-train low” in endurance-trained athletes. Br J Sports Med. 2012;46:822–7.

230. Greene NP, Fluckey JD, Lambert BS, Greene ES, Riechman SE, Crouse SF. Regulators of blood lipids and lipoproteins? PPAR and AMPK, induced by exercise, are correlated with lipids and lipoproteins in overweight/obese men and women. AJP Endocrinol Metab [Internet]. 2012;303:E1212–21. Available from: http://ajpendo.physiology.org/cgi/doi/10.1152/ajpendo.00309.2012

231. Battaglia GM, Zheng D, Hickner RC, Houmard JA. Effect of exercise training on metabolic flexibility in response to a high-fat diet in obese individuals. AJP Endocrinol Metab [Internet]. 2012;303:E1440–5. Available from: http://ajpendo.physiology.org/cgi/doi/10.1152/ajpendo.00355.2012

232. Zoladz JA, Grassi B, Majerczak J, Szkutnik Z, Korostyński M, Karasiński J, et al. Training-induced acceleration of O(2) uptake on-kinetics precedes muscle mitochondrial biogenesis in humans. Exp Physiol [Internet]. 2013;98:883–98. Available from: http://www.ncbi.nlm.nih.gov/pubmed/23204290

233. Stepto NK, Benziane B, Wadley GD, Chibalin A V, Canny BJ, Eynon N, et al. Short-term intensified cycle training alters acute and chronic responses of PGC1α and Cytochrome C oxidase IV to exercise in human skeletal muscle. PLoS One [Internet]. 2012;7:e53080. Available from: http://www.ncbi.nlm.nih.gov/pubmed/23285255

234. Sparks LM, Johannsen NM, Church TS, Earnest CP, Moonen-Kornips E, Moro C, et al. Nine months of combined training improves ex vivo skeletal muscle metabolism in individuals with type 2 diabetes. J Clin Endocrinol Metab [Internet]. 2013;98:1694–702. Available from: http://www.ncbi.nlm.nih.gov/pubmed/23463651

235. Mikus CR, Boyle LJ, Borengasser SJ, Oberlin DJ, Naples SP, Fletcher J, et al. Simvastatin impairs exercise training adaptations. J Am Coll Cardiol [Internet]. 2013;62:709–14. Available from: http://www.ncbi.nlm.nih.gov/pubmed/23583255

236. Puype J, Van Proeyen K, Raymackers J, Deldicque L, Hespel P. Sprint interval training in hypoxia stimulates glycolytic enzyme activity. Med Sci Sports Exerc [Internet]. 2013;45:2166–74. Available from: http://www.ncbi.nlm.nih.gov/pubmed/23604068

237. Gillen JB, Percival ME, Ludzki A, Tarnopolsky MA, Gibala MJ. Interval training in the fed or fasted state improves body composition and muscle oxidative capacity in overweight women. Obesity (Silver Spring) [Internet]. 2013;21:2249–55. Available from: http://www.ncbi.nlm.nih.gov/pubmed/23723099

238. Skleryk JR, Karagounis LG, Hawley JA, Sharman MJ, Laursen PB, Watson G. Two weeks of reduced-volume sprint interval or traditional exercise training does not improve metabolic functioning in sedentary obese men. Diabetes Obes Metab [Internet]. 2013;15:1146–53. Available from: http://www.ncbi.nlm.nih.gov/pubmed/23802920

239. Boyd JC, Simpson CA, Jung ME, Gurd BJ. Reducing the intensity and volume of interval training diminishes cardiovascular adaptation but not mitochondrial biogenesis in overweight/obese men. PLoS One [Internet]. 2013;8:e68091. Available from: http://www.ncbi.nlm.nih.gov/pubmed/23861854

240. Konopka AR, Suer MK, Wolff CA, Harber MP. Markers of human skeletal muscle mitochondrial biogenesis and quality control: effects of age and aerobic exercise training. J Gerontol A Biol Sci Med Sci [Internet]. 2014;69:371–8. Available from: http://www.ncbi.nlm.nih.gov/pubmed/23873965

241. Alemo Munters L, Dastmalchi M, Katz A, Esbjörnsson M, Loell I, Hanna B, et al. Improved exercise performance and increased aerobic capacity after endurance training of patients with stable polymyositis and dermatomyositis. Arthritis Res Ther [Internet]. 2013;15:R83. Available from: http://www.ncbi.nlm.nih.gov/pubmed/23941324

242. Egan B, O'Connor PL, Zierath JR, O'Gorman DJ. Time course analysis reveals gene-specific transcript and protein kinetics of adaptation to short-term aerobic exercise training in human skeletal muscle. PLoS One [Internet]. 2013;8:e74098. Available from: http://www.ncbi.nlm.nih.gov/pubmed/24069271

243. Devries MC, Samjoo IA, Hamadeh MJ, McCready C, Raha S, Watt MJ, et al. Endurance training modulates intramyocellular lipid compartmentalization and morphology in skeletal muscle of lean and obese women. J Clin Endocrinol Metab [Internet]. 2013;98:4852–62. Available from: http://www.ncbi.nlm.nih.gov/pubmed/24081737

244. Desplanches D, Amami M, Dupré-Aucouturier S, Valdivieso P, Schmutz S, Mueller M, et al. Hypoxia refines plasticity of mitochondrial respiration to repeated muscle work. Eur J Appl Physiol [Internet]. 2014;114:405–17. Available from: http://www.ncbi.nlm.nih.gov/pubmed/24327174

245. Rosenkilde M, Reichkendler MH, Auerbach P, Bonne TC, Sjödin A, Ploug T, et al. Changes in peak fat oxidation in response to different doses of endurance training. Scand J Med Sci Sports [Internet]. 2015;25:41–52. Available from: http://www.ncbi.nlm.nih.gov/pubmed/24350597

246. Ryan AS, Katzel LI, Prior SJ, McLenithan JC, Goldberg AP, Ortmeyer HK. Aerobic exercise plus weight loss improves insulin sensitivity and increases skeletal muscle glycogen synthase activity in older men. J Gerontol A Biol Sci Med Sci [Internet]. 2014;69:790–8. Available from: http://www.ncbi.nlm.nih.gov/pubmed/24357038

247. Broskey NT, Greggio C, Boss A, Boutant M, Dwyer A, Schlueter L, et al. Skeletal muscle mitochondria in the elderly: effects of physical fitness and exercise training. J Clin Endocrinol Metab [Internet]. 2014;99:1852–61. Available from: http://www.ncbi.nlm.nih.gov/pubmed/24438376

248. Olesen J, Gliemann L, Biensø R, Schmidt J, Hellsten Y, Pilegaard H. Exercise training, but not resveratrol, improves metabolic and inflammatory status in skeletal muscle of aged men. J Physiol [Internet]. 2014;592:1873–86. Available from: http://www.ncbi.nlm.nih.gov/pubmed/24514907

249. Hatle H, Støbakk PK, Mølmen HE, Brønstad E, Tjønna AE, Steinshamn S, et al. Effect of 24 sessions of high-intensity aerobic interval training carried out at either high or moderate frequency, a randomized trial. PLoS One [Internet]. 2014;9:e88375. Available from: http://www.ncbi.nlm.nih.gov/pubmed/24516645

250. Cochran AJR, Percival ME, Tricarico S, Little JP, Cermak N, Gillen JB, et al. Intermittent and continuous high-intensity exercise training induce similar acute but different chronic muscle adaptations. Exp Physiol [Internet]. 2014;99:782–91. Available from: http://www.ncbi.nlm.nih.gov/pubmed/24532598

251. Scalzo RL, Peltonen GL, Binns SE, Shankaran M, Giordano GR, Hartley D a., et al. Greater muscle protein synthesis and mitochondrial biogenesis in males compared with females during sprint interval training. FASEB J. 2014;28:2705–14.

252. Larsen S, Danielsen JH, Søndergård SD, Søgaard D, Vigelsoe A, Dybboe R, et al. The effect of high-intensity training on mitochondrial fat oxidation in skeletal muscle and subcutaneous adipose tissue. Scand J Med Sci Sports [Internet]. 2015;25:e59-69. Available from: http://www.ncbi.nlm.nih.gov/pubmed/24845952

253. Zoladz JA, Grassi B, Majerczak J, Szkutnik Z, Korostyński M, Grandys M, et al. Mechanisms responsible for the acceleration of pulmonary V̇O2 on-kinetics in humans after prolonged endurance training. Am J Physiol Regul Integr Comp Physiol [Internet]. 2014;307:R1101-14. Available from: http://www.ncbi.nlm.nih.gov/pubmed/25163914

254. Gram M, Vigelsø A, Yokota T, Hansen CN, Helge JW, Hey-Mogensen M, et al. Two weeks of one-leg immobilization decreases skeletal muscle respiratory capacity equally in young and elderly men. Exp Gerontol [Internet]. Elsevier Inc.; 2014;58:269–78. Available from: http://dx.doi.org/10.1016/j.exger.2014.08.013

255. Gillen JB, Percival ME, Skelly LE, Martin BJ, Tan RB, Tarnopolsky MA, et al. Three minutes of all-out intermittent exercise per week increases skeletal muscle oxidative capacity and improves cardiometabolic health. PLoS One. 2014;9:1–9.

256. Psilander N, Frank P, Flockhart M, Sahlin K. Adding strength to endurance training does not enhance aerobic capacity in cyclists. Scand J Med Sci Sports [Internet]. 2015;25:e353-9. Available from: http://www.ncbi.nlm.nih.gov/pubmed/25438613

257. Vincent G, Lamon S, Gant N, Vincent PJ, MacDonald JR, Markworth JF, et al. Changes in mitochondrial function and mitochondria associated protein expression in response to 2-weeks of high intensity interval training. Front Physiol

[Internet]. 2015;6:51. Available from: http://www.ncbi.nlm.nih.gov/pubmed/25759671

258. Cochran AJ, Myslik F, MacInnis MJ, Percival ME, Bishop D, Tarnopolsky MA, et al. Manipulating Carbohydrate Availability Between Twice-Daily Sessions of High-Intensity Interval Training Over 2 Weeks Improves Time-Trial Performance. Int J Sport Nutr Exerc Metab [Internet]. 2015;25:463–70. Available from: http://www.ncbi.nlm.nih.gov/pubmed/25811132

259. Cochran AJR, Percival ME, Thompson S, Gillen JB, MacInnis MJ, Potter MA, et al. β-Alanine Supplementation Does Not Augment the Skeletal Muscle Adaptive Response to 6 Weeks of Sprint Interval Training. Int J Sport Nutr Exerc Metab [Internet]. 2015;25:541–9. Available from: http://www.ncbi.nlm.nih.gov/pubmed/26008634

260. Nordsborg NB, Connolly L, Weihe P, Iuliano E, Krustrup P, Saltin B, et al. Oxidative capacity and glycogen content increase more in arm than leg muscle in sedentary women after intense training. J Appl Physiol [Internet]. 2015;119:116–23. Available from: http://www.ncbi.nlm.nih.gov/pubmed/26023221

261. Ihsan M, Markworth JF, Watson G, Choo HC, Govus A, Pham T, et al. Regular postexercise cooling enhances mitochondrial biogenesis through AMPK and p38 MAPK in human skeletal muscle. Am J Physiol Regul Integr Comp Physiol [Internet]. 2015;309:R286-94. Available from: http://www.ncbi.nlm.nih.gov/pubmed/26041108

262. Prior SJ, Goldberg AP, Ortmeyer HK, Chin ER, Chen D, Blumenthal JB, et al. Increased Skeletal Muscle Capillarization Independently Enhances Insulin Sensitivity in Older Adults After Exercise Training and Detraining. Diabetes [Internet]. 2015;64:3386–95. Available from: http://www.ncbi.nlm.nih.gov/pubmed/26068543

263. Montero D, Cathomen A, Jacobs RA, Flück D, de Leur J, Keiser S, et al. Haematological rather than skeletal muscle adaptations contribute to the increase in peak oxygen uptake induced by moderate endurance training. J Physiol [Internet]. 2015;593:4677–88. Available from: http://www.ncbi.nlm.nih.gov/pubmed/26282186

264. Coen PM, Menshikova E V, Distefano G, Zheng D, Tanner CJ, Standley RA, et al. Exercise and Weight Loss Improve Muscle Mitochondrial Respiration, Lipid Partitioning, and Insulin Sensitivity After Gastric Bypass Surgery. Diabetes [Internet]. 2015;64:3737–50. Available from: http://www.ncbi.nlm.nih.gov/pubmed/26293505

265. Larsen FJ, Schiffer TA, Ørtenblad N, Zinner C, Morales-Alamo D, Willis SJ, et al. High-intensity sprint training inhibits mitochondrial respiration through aconitase inactivation. FASEB J [Internet]. 2016;30:417–27. Available from: http://www.ncbi.nlm.nih.gov/pubmed/26452378

266. Morrison D, Hughes J, Della Gatta PA, Mason S, Lamon S, Russell AP, et al. Vitamin C and E supplementation prevents some of the cellular adaptations to endurance-training in humans. Free Radic Biol Med [Internet]. Elsevier; 2015;89:852–62. Available from: http://dx.doi.org/10.1016/j.freeradbiomed.2015.10.412

267. Granata C, Oliveira RSF, Little JP, Renner K, Bishop DJ. Training intensity modulates changes in PGC-1α and p53 protein content and mitochondrial respiration, but not markers of mitochondrial content in human skeletal muscle. FASEB J [Internet]. 2016;30:959–70. Available from: http://www.ncbi.nlm.nih.gov/pubmed/26572168

268. Boushel R, Gnaiger E, Larsen FJ, Helge JW, González-Alonso J, Ara I, et al. Maintained peak leg and pulmonary VO2 despite substantial reduction in muscle mitochondrial capacity. Scand J Med Sci Sports [Internet]. 2015;25 Suppl 4:135–43. Available from: http://www.ncbi.nlm.nih.gov/pubmed/26589127

269. Mora-Rodríguez R, Sanchez-Roncero A, Fernández-Elías VE, Guadalupe-Grau A, Ortega JF, Dela F, et al. Aerobic Exercise Training Increases Muscle Water Content in Obese Middle-Age Men. Med Sci Sports Exerc [Internet]. 2016;48:822–8. Available from: http://www.ncbi.nlm.nih.gov/pubmed/26694843

270. Christensen PM, Jacobs RA, Bonne T, Flück D, Bangsbo J, Lundby C. A short period of high-intensity interval training improves skeletal muscle mitochondrial function and pulmonary oxygen uptake kinetics. J Appl Physiol [Internet]. 2016;120:1319–27. Available from: http://www.ncbi.nlm.nih.gov/pubmed/26846547

271. Nyberg M, Fiorenza M, Lund A, Christensen M, Rømer T, Piil P, et al. Adaptations to Speed Endurance Training in Highly Trained Soccer Players. Med Sci Sports Exerc [Internet]. 2016;48:1355–64. Available from: http://www.ncbi.nlm.nih.gov/pubmed/26885636

272. Gillen JB, Martin BJ, MacInnis MJ, Skelly LE, Tarnopolsky MA, Gibala MJ. Twelve Weeks of Sprint Interval Training Improves Indices of Cardiometabolic Health Similar to Traditional Endurance Training despite a Five-Fold Lower Exercise Volume and Time Commitment. PLoS One [Internet]. 2016;11:e0154075. Available from: http://dx.doi.org/10.1371/journal.pone.0154075

273. Stray-Gundersen J, Howden EJ, Parsons DB, Thompson JR. Neither Hematocrit Normalization nor Exercise Training Restores Oxygen Consumption to Normal Levels in Hemodialysis Patients. J Am Soc Nephrol [Internet]. 2016;27:3769–79. Available from: http://www.ncbi.nlm.nih.gov/pubmed/27153927

274. Aguiar PF, Magalhães SM, Fonseca IAT, da Costa Santos VB, de Matos MA, Peixoto MFD, et al. Post-exercise cold water immersion does not alter high intensity interval training-induced exercise performance and Hsp72 responses, but enhances mitochondrial markers. Cell Stress Chaperones [Internet]. Cell Stress and Chaperones; 2016;21:793–804. Available from: http://dx.doi.org/10.1007/s12192-016-0704-6

275. MacInnis MJ, Zacharewicz E, Martin BJ, Haikalis ME, Skelly LE, Tarnopolsky MA, et al. Superior mitochondrial adaptations in human skeletal muscle after interval compared to continuous single-leg cycling matched for total work. J Physiol. 2017;595:2955–68.

276. Granata C, Oliveira RSF, Little JP, Renner K, Bishop DJ. Mitochondrial adaptations to high-volume exercise training are rapidly reversed after a reduction in training volume in human skeletal muscle. FASEB J [Internet]. 2016;30:3413–23. Available from: http://www.ncbi.nlm.nih.gov/pubmed/27402675

277. Bankolé L-C, Millet GY, Temesi J, Bachasson D, Ravelojaona M, Wuyam B, et al. Safety and efficacy of a 6-month home-based exercise program in patients with facioscapulohumeral muscular dystrophy: A randomized controlled trial. Medicine (Baltimore) [Internet]. 2016;95:e4497. Available from: http://www.ncbi.nlm.nih.gov/pubmed/27495097

278. Morville T, Rosenkilde M, Munch-andersen T, Andersen PR, K KKJRG, Helbo S, et al. Repeated Prolonged Exercise Decreases Maximal Fat Oxidation in Older Men. 2017;308–16.

279. Zinner C, Morales-Alamo D, Ørtenblad N, Larsen FJ, Schiffer TA, Willis SJ, et al. The Physiological Mechanisms of Performance Enhancement with Sprint Interval Training Differ between the Upper and Lower Extremities in Humans. Front Physiol [Internet]. 2016;7:426. Available from: http://www.ncbi.nlm.nih.gov/pubmed/27746738

280. Skovgaard C, Almquist NW, Bangsbo J. Effect of increased and maintained frequency of speed endurance training on performance and muscle adaptations in runners. J Appl Physiol [Internet]. 2017;122:48–59. Available from: http://www.ncbi.nlm.nih.gov/pubmed/27856713

281. Greggio C, Jha P, Kulkarni SS, Lagarrigue S, Broskey NT, Boutant M, et al. Enhanced Respiratory Chain Supercomplex Formation in Response to Exercise in Human Skeletal Muscle. Cell Metab [Internet]. 2017;25:301–11. Available from: http://www.ncbi.nlm.nih.gov/pubmed/27916530

282. Brocherie F, Millet GP, D'Hulst G, Van Thienen R, Deldicque L, Girard O. Repeated maximal-intensity hypoxic exercise superimposed to hypoxic residence boosts skeletal muscle transcriptional responses in elite team-sport athletes. Acta Physiol (Oxf) [Internet]. 2018;222. Available from: http://www.ncbi.nlm.nih.gov/pubmed/28103427

283. Menshikova E V, Ritov VB, Dube JJ, Amati F, Stefanovic-Racic M, Toledo FGS, et al. Calorie Restriction-induced Weight Loss and Exercise Have Differential Effects on Skeletal Muscle Mitochondria Despite Similar Effects on Insulin Sensitivity. J Gerontol A Biol Sci Med Sci [Internet]. 2017;73:81–7. Available from: http://www.ncbi.nlm.nih.gov/pubmed/28158621

284. Bonafiglia JT, Edgett BA, Baechler BL, Nelms MW, Simpson CA, Quadrilatero J, et al. Acute upregulation of PGC-1α mRNA correlates with training-induced increases in SDH activity in human skeletal muscle. Appl Physiol Nutr Metab [Internet]. 2017;42:656–66. Available from: http://www.ncbi.nlm.nih.gov/pubmed/28177701

285. Nyberg M, Egelund J, Mandrup CM, Andersen CB, Hansen KMBE, Hergel IF, et al. Leg vascular and skeletal muscle mitochondrial adaptations to aerobic high-intensity exercise training are enhanced in the early postmenopausal phase. J Physiol [Internet]. 2017;595:2969–83. Available from: http://www.ncbi.nlm.nih.gov/pubmed/28231611

286. Wyckelsma VL, Levinger I, McKenna MJ, Formosa LE, Ryan MT, Petersen AC, et al. Preservation of skeletal muscle mitochondrial content in older adults: relationship between mitochondria, fibre type and high-intensity exercise training. J Physiol [Internet]. 2017;595:3345–59. Available from: http://www.ncbi.nlm.nih.gov/pubmed/28251664

287. Guadalupe-Grau A, Fernández-Elías VE, Ortega JF, Dela F, Helge JW, Mora-Rodriguez R. Effects of 6-month aerobic interval training on skeletal muscle metabolism in middle-aged metabolic syndrome patients. Scand J Med Sci Sports [Internet]. 2018;28:585–95. Available from: http://www.ncbi.nlm.nih.gov/pubmed/28321925

288. Valdivieso P, Toigo M, Hoppeler H, Flück M. T/T homozygosity of the tenascin-C gene polymorphism rs2104772 negatively influences exercise-induced angiogenesis. PLoS One [Internet]. 2017;12:e0174864. Available from: http://www.ncbi.nlm.nih.gov/pubmed/28384286

289. Brinkmann C, Przyklenk A, Metten A, Schiffer T, Bloch W, Brixius K, et al. Influence of endurance training on skeletal muscle mitophagy regulatory proteins in type 2 diabetic men. Endocr Res [Internet]. Taylor & Francis; 2017;42:325–30. Available from: https://doi.org/10.1080/07435800.2017.1323914

290. Ortmeyer HK, Goldberg AP, Ryan AS. Exercise with weight loss improves adipose tissue and skeletal muscle markers of fatty acid metabolism in postmenopausal women. Obesity (Silver Spring) [Internet]. 2017;25:1246–53. Available from: http://www.ncbi.nlm.nih.gov/pubmed/28547918

291. Meinild Lundby A-K, Jacobs RA, Gehrig S, de Leur J, Hauser M, Bonne TC, et al. Exercise training increases skeletal muscle mitochondrial volume density by enlargement of existing mitochondria and not de novo biogenesis. Acta Physiol (Oxf) [Internet]. 2018;222. Available from: http://www.ncbi.nlm.nih.gov/pubmed/28580772

292. Broatch JR, Petersen A, Bishop DJ. Cold-water immersion following sprint interval training does not alter endurance signaling pathways or training adaptations in human skeletal muscle. Am J Physiol Regul Integr Comp Physiol [Internet]. 2017;313:R372–84. Available from: http://www.ncbi.nlm.nih.gov/pubmed/28679683

293. Gejl KD, Thams LB, Hansen M, Rokkedal-Lausch T, Plomgaard P, Nybo L, et al. No Superior Adaptations to Carbohydrate Periodization in Elite Endurance Athletes. Med Sci Sports Exerc [Internet]. 2017;49:2486–97. Available from: http://www.ncbi.nlm.nih.gov/pubmed/28723843

294. Shepherd SO, Cocks M, Meikle PJ, Mellett NA, Ranasinghe AM, Barker TA, et al. Lipid droplet remodelling and reduced muscle ceramides following sprint interval and moderate-intensity continuous exercise training in obese males. Int J Obes (Lond) [Internet]. Nature Publishing Group; 2017;41:1745–54. Available from: http://www.ncbi.nlm.nih.gov/pubmed/28736444

295. Fransson D, Nielsen TS, Olsson K, Christensson T, Bradley PS, Fatouros IG, et al. Skeletal muscle and performance adaptations to high-intensity training in elite male soccer players: speed endurance runs versus small-sided game training. Eur J Appl Physiol [Internet]. Springer Berlin Heidelberg; 2018;118:111–21. Available from: http://www.ncbi.nlm.nih.gov/pubmed/29119246

296. Mueller SM, Gehrig SM, Petersen JA, Frese S, Mihaylova V, Ligon-Auer M, et al. Effects of endurance training on skeletal muscle mitochondrial function in Huntington disease patients. Orphanet J Rare Dis [Internet]. Orphanet Journal of Rare Diseases; 2017;12:184. Available from: http://www.ncbi.nlm.nih.gov/pubmed/29258585

297. Kelly DT, Tobin C, Egan B, McCarren A, O'Connor PL, McCaffrey N, et al. Comparison of Sprint Interval and Endurance Training in Team Sport Athletes. J strength Cond Res [Internet]. 2018;32:3051–8. Available from: http://www.ncbi.nlm.nih.gov/pubmed/29373432

298. Skovgaard C, Christiansen D, Christensen PM, Almquist NW, Thomassen M, Bangsbo J. Effect of speed endurance training and reduced training volume on running economy and single muscle fiber adaptations in trained runners. Physiol Rep [Internet]. 2018;6:1–12. Available from: http://www.ncbi.nlm.nih.gov/pubmed/29417745

299. Raleigh JP, Giles MD, Islam H, Nelms M, Bentley RF, Jones JH, et al. Contribution of central and peripheral adaptations to changes in maximal oxygen uptake following 4 weeks of sprint interval training. Appl Physiol Nutr Metab [Internet]. 2018;43:1059–68. Available from: http://www.ncbi.nlm.nih.gov/pubmed/29733694

300. Mijwel S, Cardinale DA, Norrbom J, Chapman M, Ivarsson N, Wengström Y, et al. Exercise training during chemotherapy preserves skeletal muscle fiber area, capillarization, and mitochondrial content in patients with breast cancer. FASEB J [Internet]. 2018;32:5495–505. Available from: http://www.ncbi.nlm.nih.gov/pubmed/29750574

301. Wolff CA, Konopka AR, Suer MK, Trappe TA, Kaminsky LA, Harber MP. Increased cardiorespiratory fitness and skeletal muscle size following single-leg knee extension exercise training. J Sports Med Phys Fitness [Internet]. 2019;59:934–40. Available from: http://www.ncbi.nlm.nih.gov/pubmed/29845842

302. Conceição MS, Junior EMM, Telles GD, Libardi CA, Castro A, Andrade ALL, et al. Augmented Anabolic Responses after 8-wk Cycling with Blood Flow Restriction. Med Sci Sports Exerc [Internet]. 2019;51:84–93. Available from: http://www.ncbi.nlm.nih.gov/pubmed/30113523

303. Arribat Y, Broskey NT, Greggio C, Boutant M, Conde Alonso S, Kulkarni SS, et al. Distinct patterns of skeletal muscle mitochondria fusion, fission and mitophagy upon duration of exercise training. Acta Physiol (Oxf) [Internet]. 2019;225:e13179. Available from: http://www.ncbi.nlm.nih.gov/pubmed/30144291

304. Esposito F, Mathieu-Costello O, Wagner PD, Richardson RS. Acute and chronic exercise in patients with heart failure with reduced ejection fraction: evidence of structural and functional plasticity and intact angiogenic signalling in skeletal muscle. J Physiol [Internet]. 2018;596:5149–61. Available from: http://www.ncbi.nlm.nih.gov/pubmed/30192995

305. Richardson RS, Wagner H, Mudaliar SRD, Saucedo E, Henry R, Wagner PD. Exercise adaptation attenuates VEGF gene expression in human skeletal muscle. Am J Physiol Heart Circ Physiol [Internet]. 2000;279:H772-8. Available from: http://www.ncbi.nlm.nih.gov/pubmed/10924077

306. Dohlmann TL, Hindsø M, Dela F, Helge JW, Larsen S. High-intensity interval training changes mitochondrial respiratory capacity differently in adipose tissue and skeletal muscle. Physiol Rep [Internet]. 2018;6:e13857. Available from: http://www.ncbi.nlm.nih.gov/pubmed/30221839

307. Axelrod CL, Fealy CE, Mulya A, Kirwan JP. Exercise training remodels human skeletal muscle mitochondrial fission and fusion machinery towards a pro-elongation phenotype. Acta Physiol (Oxf) [Internet]. 2019;225:e13216. Available from:

http://www.ncbi.nlm.nih.gov/pubmed/30408342

308. Miyamoto-Mikami E, Tsuji K, Horii N, Hasegawa N, Fujie S, Homma T, et al. Gene expression profile of muscle adaptation to high-intensity intermittent exercise training in young men. Sci Rep [Internet]. 2018;8:16811. Available from: http://www.ncbi.nlm.nih.gov/pubmed/30429512

309. Flores-Opazo M, Boland E, Garnham A, Murphy RM, McGee SL, Hargreaves M. Exercise and GLUT4 in human subcutaneous adipose tissue. Physiol Rep [Internet]. 2018;6:e13918. Available from: http://www.ncbi.nlm.nih.gov/pubmed/30450826

310. Hedges CP, Woodhead JST, Wang HW, Mitchell CJ, Cameron-Smith D, Hickey AJR, et al. Peripheral blood mononuclear cells do not reflect skeletal muscle mitochondrial function or adaptation to high-intensity interval training in healthy young men. J Appl Physiol [Internet]. 2019;126:454–61. Available from: http://www.ncbi.nlm.nih.gov/pubmed/30571281

311. Newman AA, Grimm NC, Wilburn JR, Schoenberg HM, Trikha SRJ, Luckasen GJ, et al. Influence of Sodium Glucose Cotransporter 2 Inhibition on Physiological Adaptation to Endurance Exercise Training. J Clin Endocrinol Metab [Internet]. 2019;104:1953–66. Available from: http://www.ncbi.nlm.nih.gov/pubmed/30597042

312. Schwarz NA, Blahnik ZJ, Prahadeeswaran S, McKinley-Barnard SK, Holden SL, Waldhelm A. (-)-Epicatechin Supplementation Inhibits Aerobic Adaptations to Cycling Exercise in Humans. Front Nutr [Internet]. 2018;5:132. Available from: http://www.ncbi.nlm.nih.gov/pubmed/30622947

313. Gunnarsson TP, Brandt N, Fiorenza M, Hostrup M, Pilegaard H, Bangsbo J. Inclusion of sprints in moderate intensity continuous training leads to muscle oxidative adaptations in trained individuals. Physiol Rep [Internet]. 2019;7:e13976. Available from: http://www.ncbi.nlm.nih.gov/pubmed/30793541

314. Fritzen AM, Thøgersen FB, Thybo K, Vissing CR, Krag TO, Ruiz-Ruiz C, et al. Adaptations in Mitochondrial Enzymatic Activity Occurs Independent of Genomic Dosage in Response to Aerobic Exercise Training and Deconditioning in Human Skeletal Muscle. Cells [Internet]. 2019;8:237. Available from: http://www.ncbi.nlm.nih.gov/pubmed/30871120

315. Fiorenza M, Lemminger AK, Marker M, Eibye K, Iaia FM, Bangsbo J, et al. High-intensity exercise training enhances mitochondrial oxidative phosphorylation efficiency in a temperature-dependent manner in human skeletal muscle: implications for exercise performance. FASEB J [Internet]. www.fasebj.org; 2019;33:8976–89. Available from: http://www.ncbi.nlm.nih.gov/pubmed/31136218

316. Ghiarone T, Andrade-Souza VA, Learsi SK, Tomazini F, Ataide-Silva T, Sansonio A, et al. Twice-a-day training improves mitochondrial efficiency, but not mitochondrial biogenesis, compared with once-daily training. J Appl Physiol [Internet]. 2019;127:713–25. Available from: http://www.ncbi.nlm.nih.gov/pubmed/31246557

317. MacInnis MJ, Skelly LE, Godkin FE, Martin BJ, Tripp TR, Tarnopolsky MA, et al. Effect of short-term, high-intensity exercise training on human skeletal muscle citrate synthase maximal activity: single versus multiple bouts per session. Appl Physiol Nutr Metab [Internet]. 2019;44:1391–4. Available from: http://www.ncbi.nlm.nih.gov/pubmed/31618598

318. Bylund AC, Bjurö T, Cederblad G, Holm J, Lundholm K, Sjöstroöm M, et al. Physical training in man. Skeletal muscle metabolism in relation to muscle morphology and running ability. Eur J Appl Physiol Occup Physiol [Internet]. 1977;36:151–69. Available from: http://www.ncbi.nlm.nih.gov/pubmed/323004

319. Ferguson RJ, Taylor AW, Côté P, Charlebois J, Dinelle Y, Péronnet F, et al. Skeletal muscle and cardiac changes with training in patients with angina pectoris. Am J Physiol [Internet]. 1982;243:H830-6. Available from: http://www.ncbi.nlm.nih.gov/pubmed/6215866

320. Svedenhag J, Henriksson J, Juhlin-Dannfelt A. Beta-adrenergic blockade and training in human subjects: effects on muscle metabolic capacity. Am J Physiol. 1984;247:E305–11.

321. Chrøis KM, Dohlmann TL, Søgaard D, Hansen CV, Dela F, Helge JW, et al. Mitochondrial adaptations to high intensity interval training in older females and males. Eur J Sport Sci [Internet]. Taylor & Francis; 2020;20:135–45. Available from: http://www.ncbi.nlm.nih.gov/pubmed/31145037

322. Olek RA, Kujach S, Ziemann E, Ziolkowski W, Waz P, Laskowski R. Adaptive Changes After 2 Weeks of 10-s Sprint Interval Training With Various Recovery Times. Front Physiol [Internet]. 2018;9:392. Available from: http://www.ncbi.nlm.nih.gov/pubmed/29719513

323. Holm J, Dahllöf AG, Scherstén T. Metabolic activity of skeletal muscle in patients with peripheral arterial insufficiency. Effect of arterial reconstructive surgery. Scand J Clin Lab Invest [Internet]. 1975;35:81–6. Available from: http://www.ncbi.nlm.nih.gov/pubmed/1129595

324. St-Pierre D, Brassard L, Ferguson RJ, Montpetit RR, Taylor AW. The effects of endurance and power training on skeletal muscle enzyme activities in young females. J Sports Med Phys Fitness [Internet]. 1983;23:281–5. Available from:

http://www.ncbi.nlm.nih.gov/pubmed/6228691

325. Lithell H, Krotkiewski M, Kiens B, Wroblewski Z, Holm G, Strömblad G, et al. Non-response of muscle capillary density and lipoprotein-lipase activity to regular training in diabetic patients. Diabetes Res [Internet]. 1985;2:17–21. Available from: http://www.ncbi.nlm.nih.gov/pubmed/3995872

326. Vogt M, Puntschart A, Geiser J, Zuleger C, Billeter R, Hoppeler H. Molecular adaptations in human skeletal muscle to endurance training under simulated hypoxic conditions. J Appl Physiol [Internet]. 2001;91:173–82. Available from: http://www.ncbi.nlm.nih.gov/pubmed/11408428

327. Mitchell EA, Martin NRW, Turner MC, Taylor CW, Ferguson RA. The combined effect of sprint interval training and postexercise blood flow restriction on critical power, capillary growth, and mitochondrial proteins in trained cyclists. J Appl Physiol. 2019;126:51–9.

328. Watson EL, Baker LA, Wilkinson TJ, Gould DW, Graham-Brown MPM, Major RW, et al. Reductions in skeletal muscle mitochondrial mass are not restored following exercise training in patients with chronic kidney disease. FASEB J [Internet]. 2020;34:1755–67. Available from: http://www.ncbi.nlm.nih.gov/pubmed/31914685

329. Morales-Palomo F, Ramirez-Jimenez M, Ortega JF, Moreno-Cabañas A, Mora-Rodriguez R. Exercise Training Adaptations in Metabolic Syndrome Individuals on Chronic Statin Treatment. J Clin Endocrinol Metab [Internet]. 2020;105:1–24. Available from: http://www.ncbi.nlm.nih.gov/pubmed/31875915

330. Merlet AN, Féasson L, Bartolucci P, Hourdé C, Schwalm C, Gellen B, et al. Muscle structural, energetic and functional benefits of endurance exercise training in sickle cell disease. Am J Hematol [Internet]. 2020;95:1257–68. Available from: http://www.ncbi.nlm.nih.gov/pubmed/32681734

331. Dela F, Ingersen A, Andersen NB, Nielsen MB, Petersen HHH, Hansen CN, et al. Effects of one-legged high-intensity interval training on insulin-mediated skeletal muscle glucose homeostasis in patients with type 2 diabetes. Acta Physiol (Oxf) [Internet]. 2019;226:e13245. Available from: http://www.ncbi.nlm.nih.gov/pubmed/30585698

332. Chobanyan-Jürgens K, Scheibe RJ, Potthast AB, Hein M, Smith A, Freund R, et al. Influences of Hypoxia Exercise on Whole-Body Insulin Sensitivity and Oxidative Metabolism in Older Individuals. J Clin Endocrinol Metab [Internet]. 2019;104:5238–48. Available from: http://www.ncbi.nlm.nih.gov/pubmed/30942862

333. Fiorenza M, Gunnarsson TP, Ehlers TS, Bangsbo J. High-intensity exercise training ameliorates aberrant expression of markers of mitochondrial turnover but not oxidative damage in skeletal muscle of men with essential hypertension. Acta Physiol (Oxf) [Internet]. 2019;225:e13208. Available from: http://www.ncbi.nlm.nih.gov/pubmed/30339318

334. Hoffmann C, Schneeweiss P, Randrianarisoa E, Schnauder G, Kappler L, Machann J, et al. Response of Mitochondrial Respiration in Adipose Tissue and Muscle to 8 Weeks of Endurance Exercise in Obese Subjects. J Clin Endocrinol Metab [Internet]. 2020;105. Available from: http://www.ncbi.nlm.nih.gov/pubmed/32827042

335. Hoier B, Olsen K, Hanskov DJA, Jorgensen M, Norup LR, Hellsten Y. Early time course of change in angiogenic proteins in human skeletal muscle and vascular cells with endurance training. Scand J Med Sci Sports [Internet]. 2020;30:1117–31. Available from: http://www.ncbi.nlm.nih.gov/pubmed/32246511

336. Islam H, Bonafiglia JT, Del Giudice M, Pathmarajan R, Simpson CA, Quadrilatero J, et al. Repeatability of training-induced skeletal muscle adaptations in active young males. J Sci Med Sport [Internet]. Sports Medicine Australia; 2021;24:494–8. Available from: http://www.ncbi.nlm.nih.gov/pubmed/33160857

337. Knuiman P, Van Loon LJC, Wouters J, Hopman M, Mensink M. Protein supplementation elicits greater gains in maximal oxygen uptake capacity and stimulates lean mass accretion during prolonged endurance training: A double-blind randomized controlled trial. Am J Clin Nutr. 2019;110:508–18.

338. Bonafiglia JT, Islam H, Preobrazenski N, Ma A, Deschenes M, Erlich AT, et al. Examining interindividual differences in select muscle and whole-body adaptations to continuous endurance training. Exp Physiol [Internet]. 2021;106:2168–76. Available from: https://doi.org/10.1113/EP089421

339. Skattebo Ø, Capelli C, Rud B, Auensen M, Calbet JAL, Hallén J. Increased oxygen extraction and mitochondrial protein expression after small muscle mass endurance training. Scand J Med Sci Sports [Internet]. 2020;30:1615–31. Available from: http://www.ncbi.nlm.nih.gov/pubmed/32403173

340. Skattebo Ø, Bjerring AW, Auensen M, Sarvari SI, Cumming KT, Capelli C, et al. Blood volume expansion does not explain the increase in peak oxygen uptake induced by 10 weeks of endurance training. Eur J Appl Physiol [Internet]. Springer Berlin Heidelberg; 2020;120:985–99. Available from: http://www.ncbi.nlm.nih.gov/pubmed/32172291

341. Aird TP, Farquharson AJ, Bermingham KM, O'Sulllivan A, Drew JE, Carson BP. Divergent serum metabolomic, skeletal muscle signaling, transcriptomic, and performance adaptations to fasted versus whey protein-fed sprint interval training.

Am J Physiol - Endocrinol Metab. 2021;321:E802–20.

342. Apostolopoulou M, Mastrototaro L, Hartwig S, Pesta D, Straßburger K, de Filippo E, et al. Metabolic responsiveness to training depends on insulin sensitivity and protein content of exosomes in insulin-resistant males. Sci Adv. 2021;7:1–14.

343. Cardinale DA, Gejl KD, Petersen KG, Nielsen J, Ortenblad N, Larsen FJ. Short-term intensified training temporarily impairs mitochondrial respiratory capacity in elite endurance athletes. J Appl Physiol. 2021;131:388–400.

344. Blackwell JEM, Gharahdaghi N, Brook MS, Watanabe S, Boereboom CL, Doleman B, et al. The physiological impact of high-intensity interval training in octogenarians with comorbidities. J Cachexia Sarcopenia Muscle. 2021;12:866–79.

345. Lim C, Dunford EC, Valentino SE, Oikawa SY, McGlory C, Baker SK, et al. Both Traditional and Stair Climbing-based HIIT Cardiac Rehabilitation Induce Beneficial Muscle Adaptations. Med Sci Sports Exerc. 2021;53:1114–24.

346. Baasch-Skytte T, Gunnarsson TP, Fiorenza M, Bangsbo J. Skeletal muscle proteins important for work capacity are altered with type 2 diabetes — Effect of 10-20-30 training. Physiol Rep. 2021;9:1–13.

347. Maunder E, Plews DJ, Wallis GA, Brick MJ, Leigh WB, Chang WL, et al. Temperate performance and metabolic adaptations following endurance training performed under environmental heat stress. Physiol Rep. 2021;9:1–14.

348. Granata C, Caruana NJ, Botella J, Jamnick NA, Huynh K, Kuang J, et al. High-intensity training induces non-stoichiometric changes in the mitochondrial proteome of human skeletal muscle without reorganisation of respiratory chain content. Nat Commun. Springer US; 2021;12.

349. Jacques M, Landen S, Alvarez Romero J, Yan X, Garnham A, Hiam D, et al. Individual physiological and mitochondrial responses during 12 weeks of intensified exercise. Physiol Rep. 2021;9:1–12.

350. Almquist NW, Wilhelmsen M, Ellefsen S, Sandbakk Ø, Rønnestad BR. Effects of Including Sprints in LIT Sessions during a 14-d Camp on Muscle Biology and Performance Measures in Elite Cyclists. Med Sci Sports Exerc. 2021;53:2333–45.

351. Almquist NW, Eriksen HB, Wilhelmsen M, Hamarsland H, Ing S, Ellefsen S, et al. No Differences Between 12 Weeks of Block- vs. Traditional-Periodized Training in Performance Adaptations in Trained Cyclists. Front Physiol [Internet]. 2022;13:837634. Available from: http://www.ncbi.nlm.nih.gov/pubmed/35299664

352. Gordon A, Tyni-Lenne R, Jansson E, Jensen-Urstad M, Kaijser L. Beneficial effects of exercise training in heart failure patients with low cardiac output response to exercise - a comparison of two training models. J Intern Med [Internet]. 1999;246:175–82. Available from: http://ovidsp.ovid.com/ovidweb.cgi?T=JS&CSC=Y&NEWS=N&PAGE=fulltext&D=med4&AN=10447786%5Cnhttp://nhs5531173.on.worldcat.org/atoztitles/link?sid=OVID:medline&id=pmid:10447786&id=doi:&issn=0954-6820&isbn=&volume=246&issue=2&spage=175&pages=175-82&date=1999&t

353. Tyni-Lenné R, Gordon a, Jensen-Urstad M, Dencker K, Jansson E, Sylvén C. Aerobic training involving a minor muscle mass shows greater efficiency than training involving a major muscle mass in chronic heart failure patients. J Card Fail [Internet]. 1999;5:300–7. Available from: http://www.ncbi.nlm.nih.gov/pubmed/10634671
