# Supplementary material for: Effects of Exercise Training on Mitochondrial and Capillary Growth in Human Skeletal Muscle: A Systematic Review and Meta-Regression
Source: Sports Med. 2024 Oct 10;55(1):115–44. doi: 10.1007/s40279-024-02120-2 (PMC11787188; doi:10.1007/s40279-024-02120-2)
Supplement: Supplementary file 5 — Supplementary file5 Supplementary Information 5: Reference list of research articles included in capillary analysis (PDF 159 KB) [file 40279_2024_2120_MOESM5_ESM.pdf]

References to muscle capillary research articles

1. Andersen P, Henriksson J. Capillary supply of the quadriceps femoris muscle of man: adaptive response to exercise. J Physiol. 1977;270:677–90.

2. Ingjer F. Effects of endurance training on muscle fibre ATP-ase activity, capillary supply and mitochondrial content in man. J Physiol. 1979;294:419–32.

3. Klausen K, Andersen LB, Pelle I. Adaptive changes in work capacity, skeletal muscle capillarization and enzyme levels during training and detraining. Acta Physiol Scand [Internet]. 1981;113:9–16. Available from: http://www.ncbi.nlm.nih.gov/pubmed/7315443

4. Daub WD, Green HJ, Houston ME, Thomson JA, Fraser IG, Ranney DA. Cross-adaptive responses to different forms of leg training: skeletal muscle biochemistry and histochemistry. Can J Physiol Pharmacol [Internet]. 1982;60:628–33. Available from: http://www.ncbi.nlm.nih.gov/pubmed/7104850

5. Wallberg-Henriksson H, Gunnarsson R, Henriksson J, DeFronzo R, Felig P, Ostman J, et al. Increased peripheral insulin sensitivity and muscle mitochondrial enzymes but unchanged blood glucose control in type I diabetics after physical training. Diabetes [Internet]. 1982;31:1044–50. Available from: http://www.ncbi.nlm.nih.gov/pubmed/6757018

6. Krotkiewski M, Bylund-Fallenius AC, Holm J, Bjorntorp P, Grimby G, Mandroukas K. Relationship between muscle morphology and metabolism in obese women: the effects of long-term physical training. Eur J Clin Invest [Internet]. 1983;13:5–12. Available from: http://www.ncbi.nlm.nih.gov/entrez/query.fcgi?cmd=Retrieve&db=PubMed&dopt=Citation&list_uids=6409624

7. Schantz P, Henriksson J, Jansson E. Adaptation of human skeletal muscle to endurance training of long duration. Clin Physiol [Internet]. 1983;3:141–51. Available from: http://www.ncbi.nlm.nih.gov/pubmed/6682735

8. Svedenhag J, Lithell H, Juhlin-Dannfelt A, Henriksson J. Increase in skeletal muscle lipoprotein lipase following endurance training in man. Atherosclerosis [Internet]. 1983;49:203–7. Available from: http://www.ncbi.nlm.nih.gov/pubmed/6381044

9. Mandroukas K, Krotkiewski M, Hedberg M, Wroblewski Z, Björntorp P, Grimby G. Physical training in obese women. Effects of muscle morphology, biochemistry and function. Eur J Appl Physiol Occup Physiol [Internet]. 1984;52:355–61. Available from: http://www.ncbi.nlm.nih.gov/pubmed/6381044

10. Wallberg-Henriksson H, Gunnarsson R, Henriksson J. Influence of physical training on formation of muscle capillaries in type I diabetes. Diabetes. 1984;33:851–7.

11. Rösler K, Hoppeler H, Conley KE, Claassen H, Gehr P, Howald H. Transfer effects in endurance exercise. Adaptations in trained and untrained muscles. Eur J Appl Physiol Occup Physiol [Internet]. 1985;54:355–62. Available from: http://www.ncbi.nlm.nih.gov/pubmed/4065122

12. Hoppeler H, Howald H, Conley K, Lindstedt SL, Claassen H, Vock P, et al. Endurance training in humans: aerobic capacity and structure of skeletal muscle. J Appl Physiol. 1985;59:320–7.

13. Denis C, Chatard JC, Dormois D, Linossier MT, Geyssant A, Lacour JR. Effects of endurance training on capillary supply of human skeletal muscle on two age groups (20 and 60 years). J Physiol (Paris). 1986;81:379–83.

14. Mandroukas K, Krotkiewski M, Holm G, Strömblad G, Grimby G, Lithell H, et al. Muscle adaptations and glucose control after physical training in insulin-dependent diabetes mellitus. Clin Physiol [Internet]. 1986;6:39–52. Available from: http://search.ebscohost.com/login.aspx?direct=true&db=sph&AN=SPH193187&site=ehost-live

15. Wolfel EE, Hiatt WR, Brammell HL, Carry MR, Ringel SP, Travis V, et al. Effects of selective and nonselective beta-adrenergic blockade on mechanisms of exercise conditioning. Circulation [Internet]. 1986;74:664–74. Available from: http://circ.ahajournals.org/cgi/doi/10.1161/01.CIR.74.4.664

16. Terrados N, Melichna J, Sylven C, Jansson E, Kaijser L. Effects of training at simulated altitude on performance and muscle metabolic capacity in competitive road cyclists. Eur J Appl Physiol Occup Physiol [Internet]. 1988;57:203–9. Available from: http://www.ncbi.nlm.nih.gov/pubmed/3349988

17. Allenberg K, Johansen K, Saltin B. Skeletal muscle adaptations to physical training in type II (non-insulin-dependent) diabetes mellitus. Acta Med Scand. 1988;223:365–73.

18. Kiens B, Lithell H. Lipoprotein metabolism influenced by training-induced changes in human skeletal muscle. J Clin Invest. 1989;83:558–64.

19. Sale DG, MacDougall JD, Jacobs I, Garner S. Interaction between concurrent strength and endurance training. J Appl Physiol [Internet]. 1990;68:260–70. Available from: http://www.scopus.com/inward/record.url?eid=2-s2.0-0025014889&partnerID=40&md5=2bdd11f7215f5b8607f78613bc08dd80

20. Terrados N, Jansson E, Sylvén C, Kaijser L. Is hypoxia a stimulus for synthesis of oxidative enzymes and myoglobin? J Appl Physiol. 1990;68:2369–72.

21. Keith SP, Jacobs I, McLellan TM. Adaptations to training at the individual anaerobic threshold. Eur J Appl Physiol Occup Physiol. 1992;65:316–23.

22. Coggan a R, Spina RJ, King DS, Rogers M a, Brown M, Nemeth PM, et al. Skeletal muscle adaptations to endurance training in 60- to 70-yr-old men and women. J Appl Physiol. 1992;72:1780–6.

23. Esbjörnsson M, Jansson E, Sundberg CJ, Sylvén C, Eiken O, Nygren A, et al. Muscle fibre types and enzyme activities after training with local leg ischaemia in man. Acta Physiol Scand [Internet]. 1993;148:233–41. Available from: http://www.ncbi.nlm.nih.gov/pubmed/8213179

24. Moore GE, Parsons DB, Stray-Gundersen J, Painter PL, Brinker KR, Mitchell JH. Uremic myopathy limits aerobic capacity in hemodialysis patients. Am J Kidney Dis [Internet]. National Kidney Foundation, Inc.; 1993;22:277–87. Available from: http://dx.doi.org/10.1016/S0272-6386(12)70319-0

25. Desplanches D, Hoppeler H, Linossier MT, Denis C, Claassen H, Dormois D, et al. Effects of training in normoxia and normobaric hypoxia on human muscle ultrastructure. Pflugers Arch [Internet]. 1993;425:263–7. Available from: http://www.ncbi.nlm.nih.gov/pubmed/8309787

26. Suter E, Hoppeler H, Claassen H, Billeter R, Aebi U, Horber F, et al. Ultrastructural modification of human skeletal muscle tissue with 6-month moderate-intensity exercise training. Int J Sport Med [Internet]. 1995;16:160–6. Available from: http://www.ncbi.nlm.nih.gov/entrez/query.fcgi?cmd=Retrieve&db=PubMed&dopt=Citation&list_uids=7649706

27. Belardinelli R, Georgiou D, Scocco V, Barstow TJ, Purcaro A. Low intensity exercise training in patients with chronic heart failure. J Am Coll Cardiol. 1995;26:975–82.

28. Freyssenet D, Berthon P, Denis C, Barthelemy JC, Guezennec CY, Chatard JC. Effect of a 6-week endurance training programme and branched-chain amino acid supplementation on histomorphometric characteristics of aged human muscle. Arch Physiol Biochem [Internet]. 1996;104:157–62. Available from: http://www.ncbi.nlm.nih.gov/pubmed/8818199

29. Ades PA, Waldmann ML, Meyer WL, Brown KA, Poehlman ET, Pendlebury WW, et al. Skeletal muscle and cardiovascular adaptations to exercise conditioning in older coronary patients. Circulation [Internet]. 1996;94:323–30. Available from: http://www.ncbi.nlm.nih.gov/pubmed/8759072

30. Desplanches D, Hoppeler H, Tüscher L, Mayet MH, Spielvogel H, Ferretti G, et al. Muscle tissue adaptations of high-altitude natives to training in chronic hypoxia or acute normoxia. J Appl Physiol. 1996;81:1946–51.

31. Melissa L, MacDougall JD, Tarnopolsky MA, Cipriano N, Green HJ. Skeletal muscle adaptations to training under normobaric hypoxic versus normoxic conditions. Med Sci Sports Exerc [Internet]. 1997;29:238–43. Available from: http://www.ncbi.nlm.nih.gov/pubmed/9044229

32. Hepple RT, Mackinnon SL, Goodman JM, Thomas SG, Plyley MJ. Resistance and aerobic training in older men: effects on VO2peak and the capillary supply to skeletal muscle. J Appl Physiol [Internet]. 1997;82:1305–10. Available from: http://www.ncbi.nlm.nih.gov/pubmed/9104869

33. Turner DL, Hoppeler H, Claassen H, Vock P, Kayser B, Schena F, et al. Effects of endurance training on oxidative capacity and structural composition of human arm and leg muscles. Acta Physiol Scand [Internet]. 1997;161:459–64. Available from: http://www.ncbi.nlm.nih.gov/pubmed/9429652

34. Lampert E, Mettauer B, Hoppeler H, Charloux A, Charpentier A, Lonsdorfer J. Skeletal muscle response to short endurance training in heart transplant recipients. Jacc. 1998;32:420–6.

35. Ferketich AK, Kirby TE, Alway SE. Cardiovascular and muscular adaptations to combined endurance and strength training in elderly women. Acta Physiol Scand. 1998;164:259–67.

36. Kiilavuori K, Näveri H, Salmi T, Härkönen M. The effect of physical training on skeletal muscle in patients with chronic heart failure. Eur J Hear Fail J Work Gr Hear Fail Eur Soc Cardiol. 2000;2:53–63.

37. Bell GJ, Syrotuik D, Martin TP, Burnham R, Quinney HA. Effect of concurrent strength and endurance training on skeletal muscle properties and hormone concentrations in humans. Eur J Appl Physiol [Internet]. 2000;81:418–27. Available from: http://link.springer.com/10.1007/s004210050063

38. Frandsen U, Höffner L, Betak a, Saltin B, Bangsbo J, Hellsten Y. Endurance training does not alter the level of neuronal nitric oxide synthase in human skeletal muscle. J Appl Physiol [Internet]. 2000;89:1033–8. Available from: http://www.ncbi.nlm.nih.gov/pubmed/10956347

39. Costes F, Prieur F, Féasson L, Geyssant a, Barthélémy JC, Denis C. Influence of training on NIRS muscle oxygen saturation during submaximal exercise. Med Sci Sports Exerc. 2001;33:1484–9.

40. Masuda K, Okazaki K, Kuno S, Asano K, Shimojo H, Katsuta S. Endurance training under 2500-m hypoxia does not increase myoglobin content in human skeletal muscle. Eur J Appl Physiol [Internet]. 2001;85:486–90. Available from: http://www.ncbi.nlm.nih.gov/pubmed/11606019

41. Shono N, Urata H, Saltin B, Mizuno M, Harada T, Shindo M, et al. Effects of low intensity aerobic training on skeletal muscle capillary and blood lipoprotein profiles. J Atheroscler Thromb [Internet]. 2002;9:78–85. Available from: http://www.ncbi.nlm.nih.gov/pubmed/12238642

42. Keteyian SJ, Duscha BD, Brawner CA, Green HJ, Marks CRC, Schachat FH, et al. Differential effects of exercise training in men and women with chronic heart failure. Am Heart J. 2003;145:912–8.

43. Sakkas GK, Sargeant AJ, Mercer TH, Ball D, Koufaki P, Karatzaferi C, et al. Changes in muscle morphology in dialysis patients after 6 months of aerobic exercise training. Nephrol Dial Transplant. 2003;18:1854–61.

44. Charifi N, Kadi F, Féasson L, Costes F, Geyssant A, Denis C. Enhancement of microvessel tortuosity in the vastus lateralis muscle of old men in response to endurance training. J Physiol. 2004;554:559–69.

45. Jensen L, Bangsbo J, Hellsten Y. Effect of high intensity training on capillarization and presence of angiogenic factors in human skeletal muscle. J Physiol. 2004;557:571–82.

46. Mourtzakis M, González-Alonso J, Graham TE, Saltin B. Hemodynamics and O2 uptake during maximal knee extensor exercise in untrained and trained human quadriceps muscle: effects of hyperoxia. J Appl Physiol. 2004;97:1796–802.

47. Hansen AK, Fischer CP, Plomgaard P, Andersen JL, Saltin B, Pedersen BK. Skeletal muscle adaptation: training twice every second day vs. training once daily. J Appl Physiol. 2005;98:93–9.

48. Østergård T, Andersen JL, Nyholm B, Lund S, Nair KS, Saltin B, et al. Impact of exercise training on insulin sensitivity, physical fitness, and muscle oxidative capacity in first-degree relatives of type 2 diabetic patients. Am J Physiol Endocrinol Metab [Internet]. 2006;290:E998-1005. Available from: http://www.ncbi.nlm.nih.gov/pubmed/16352678

49. Vogiatzis I, Terzis G, Nanas S, Stratakos G, Simoes DCM, Georgiadou O, et al. Skeletal muscle adaptations to interval training in patients with advanced COPD. Chest [Internet]. The American College of Chest Physicians; 2005;128:3838–45. Available from: http://dx.doi.org/10.1378/chest.128.6.3838

50. Messonnier L, Freund H, Denis C, F??asson L, Lacour JR. Effects of training on lactate kinetics parameters and their influence on short high-intensity exercise performance. Int J Sports Med. 2006;27:60–6.

51. Charles M, Charifi N, Verney J, Pichot V, Feasson L, Costes F, et al. Effect of endurance training on muscle microvascular filtration capacity and vascular bed morphometry in the elderly. Acta Physiol. 2006;187:399–406.

52. Jeppesen TD, Schwartz M, Olsen DB, Wibrand F, Krag T, Dunø M, et al. Aerobic training is safe and improves exercise capacity in patients with mitochondrial myopathy. Brain. 2006;129:3402–12.

53. Dubé JJ, Amati F, Stefanovic-Racic M, Toledo FGS, Sauers SE, Goodpaster BH. Exercise-induced alterations in intramyocellular lipids and insulin resistance: the athlete's paradox revisited. Am J Physiol Endocrinol Metab [Internet]. 2008;294:E882-8. Available from: http://www.ncbi.nlm.nih.gov/pubmed/18319352

54. Daussin FN, Zoll J, Dufour SP, Ponsot E, Lonsdorfer-Wolf E, Doutreleau S, et al. Effect of interval versus continuous training on cardiorespiratory and mitochondrial functions: relationship to aerobic performance improvements in sedentary subjects. Am J Physiol Regul Integr Comp Physiol [Internet]. 2008;295:R264-72. Available from: http://www.ncbi.nlm.nih.gov/pubmed/18417645

55. Wang J, Zhou S, Bronks R, Graham J, Myers S. Effects of supervised treadmill walking training on calf muscle capillarization in patients with intermittent claudication. Angiology [Internet]. 2009;60:36–41. Available from: http://www.ncbi.nlm.nih.gov/pubmed/18505746

56. Iaia FM, Hellsten Y, Nielsen JJ, Fernstrom M, Sahlin K, Bangsbo J. Four weeks of speed endurance training reduces energy expenditure during exercise and maintains muscle oxidative capacity despite a reduction in training volume. J Appl Physiol [Internet]. 2008;106:73–80. Available from: http://jap.physiology.org/cgi/doi/10.1152/japplphysiol.90676.2008

57. Robbins JL, Duscha BD, Bensimhon DR, Wasserman K, Hansen JE, Houmard JA, et al. A sex-specific relationship between

capillary density and anaerobic threshold. J Appl Physiol [Internet]. 2009;106:1181–6. Available from: http://www.ncbi.nlm.nih.gov/pubmed/19164774

58. Bajpeyi S, Tanner CJ, Slentz CA, Duscha BD, McCartney JS, Hickner RC, et al. Effect of exercise intensity and volume on persistence of insulin sensitivity during training cessation. J Appl Physiol [Internet]. 2009;106:1079–85. Available from: http://www.ncbi.nlm.nih.gov/pubmed/19196913

59. Bangsbo J, Nielsen JJ, Mohr M, Randers MB, Krustrup BR, Brito J, et al. Performance enhancements and muscular adaptations of a 16-week recreational football intervention for untrained women. Scand J Med Sci Sports [Internet]. 2010;20 Suppl 1:24–30. Available from: http://www.ncbi.nlm.nih.gov/pubmed/19954496

60. Krustrup P, Christensen JF, Randers MB, Pedersen H, Sundstrup E, Jakobsen MD, et al. Muscle adaptations and performance enhancements of soccer training for untrained men. Eur J Appl Physiol. 2010;108:1247–58.

61. Schmutz S, Däpp C, Wittwer M, Durieux A-C, Mueller M, Weinstein F, et al. A hypoxia complement differentiates the muscle response to endurance exercise. Exp Physiol. 2010;95:723–35.

62. Krustrup P, Hansen PR, Randers MB, Nybo L, Martone D, Andersen LJ, et al. Beneficial effects of recreational football on the cardiovascular risk profile in untrained premenopausal women. Scand J Med Sci Sports [Internet]. 2010;20 Suppl 1:40–9. Available from: http://www.ncbi.nlm.nih.gov/pubmed/20210906

63. Erbs S, Höllriegel R, Linke A, Beck EB, Adams V, Gielen S, et al. Exercise training in patients with advanced chronic heart failure (NYHA IIIb) promotes restoration of peripheral vasomotor function, induction of endogenous regeneration, and improvement of left ventricular function. Circ Heart Fail [Internet]. 2010;3:486–94. Available from: http://www.ncbi.nlm.nih.gov/pubmed/20430934

64. Kohn TA, Essén-Gustavsson B, Myburgh KH. Specific muscle adaptations in type II fibers after high-intensity interval training of well-trained runners. Scand J Med Sci Sports [Internet]. 2011;21:765–72. Available from: http://www.ncbi.nlm.nih.gov/pubmed/20492589

65. Van Proeyen K, Szlufcik K, Nielens H, Ramaekers M, Hespel P. Beneficial metabolic adaptations due to endurance exercise training in the fasted state. J Appl Physiol [Internet]. 2011;110:236–45. Available from: http://www.ncbi.nlm.nih.gov/pubmed/21051570

66. Aagaard P, Andersen JL, Bennekou M, Larsson B, Olesen JL, Crameri R, et al. Effects of resistance training on endurance capacity and muscle fiber composition in young top-level cyclists. Scand J Med Sci Sports [Internet]. 2011;21:e298-307. Available from: http://www.ncbi.nlm.nih.gov/pubmed/21362056

67. Vogiatzis I, Terzis G, Stratakos G, Cherouveim E, Athanasopoulos D, Spetsioti S, et al. Effect of pulmonary rehabilitation on peripheral muscle fiber remodeling in patients with COPD in GOLD stages II to IV. Chest [Internet]. The American College of Chest Physicians; 2011;140:744–52. Available from: http://dx.doi.org/10.1378/chest.10-3058

68. Huber-Abel FAM, Gerber M, Hoppeler H, Baum O. Exercise-induced angiogenesis correlates with the up-regulated expression of neuronal nitric oxide synthase (nNOS) in human skeletal muscle. Eur J Appl Physiol [Internet]. 2012;112:155–62. Available from: http://link.springer.com/10.1007/s00421-011-1960-x

69. St-Amand J, Yoshioka M, Nishida Y, Tobina T, Shono N, Tanaka H. Effects of mild-exercise training cessation in human skeletal muscle. Eur J Appl Physiol [Internet]. 2012;112:853–69. Available from: http://www.ncbi.nlm.nih.gov/pubmed/21681480

70. Murias JM, Kowalchuk JM, Ritchie D, Hepple RT, Doherty TJ, Paterson DH. Adaptations in capillarization and citrate synthase activity in response to endurance training in older and young men. J Gerontol A Biol Sci Med Sci [Internet]. 2011;66:957–64. Available from: http://www.ncbi.nlm.nih.gov/pubmed/21715648

71. Duscha BD, Robbins JL, Jones WS, Kraus WE, Lye RJ, Sanders JM, et al. Angiogenesis in skeletal muscle precede improvements in peak oxygen uptake in peripheral artery disease patients. Arterioscler Thromb Vasc Biol [Internet]. 2011;31:2742–8. Available from: http://www.ncbi.nlm.nih.gov/pubmed/21868709

72. Esposito F, Reese V, Shabetai R, Wagner PD, Richardson RS. Isolated quadriceps training increases maximal exercise capacity in chronic heart failure: The role of skeletal muscle convective and diffusive oxygen transport. J Am Coll Cardiol [Internet]. Elsevier Inc.; 2011;58:1353–62. Available from: http://dx.doi.org/10.1016/j.jacc.2011.06.025

73. Hoier B, Nordsborg N, Andersen S, Jensen L, Nybo L, Bangsbo J, et al. Pro- and anti-angiogenic factors in human skeletal muscle in response to acute exercise and training. J Physiol. 2012;590:595–606.

74. Duscha BD, Annex BH, Johnson JL, Huffman K, Houmard J, Kraus WE. Exercise dose response in muscle. Int J Sports Med. 2012;33:218–23.

75. Jones WS, Duscha BD, Robbins JL, Duggan NN, Regensteiner JG, Kraus WE, et al. Alteration in angiogenic and anti-angiogenic forms of vascular endothelial growth factor-A in skeletal muscle of patients with intermittent claudication following exercise training. Vasc Med (United Kingdom). 2012;17:94–100.

76. Green HJ, Burnett M, Kollias H, Ouyang J, Smith I, Tupling S. Can increases in capillarization explain the early adaptations in metabolic regulation in human muscle to short-term training? Can J Physiol Pharmacol [Internet]. 2012;90:557–66. Available from: http://www.nrcresearchpress.com/doi/10.1139/y2012-013

77. Zoladz JA, Grassi B, Majerczak J, Szkutnik Z, Korostyński M, Karasiński J, et al. Training-induced acceleration of O(2) uptake on-kinetics precedes muscle mitochondrial biogenesis in humans. Exp Physiol [Internet]. 2013;98:883–98. Available from: http://www.ncbi.nlm.nih.gov/pubmed/23204290

78. Desplanches D, Amami M, Dupré-Aucouturier S, Valdivieso P, Schmutz S, Mueller M, et al. Hypoxia refines plasticity of mitochondrial respiration to repeated muscle work. Eur J Appl Physiol [Internet]. 2014;114:405–17. Available from: http://www.ncbi.nlm.nih.gov/pubmed/24327174

79. Boushel R, Ara I, Gnaiger E, Helge JW, González-Alonso J, Munck-Andersen T, et al. Low-intensity training increases peak arm VO2 by enhancing both convective and diffusive O2 delivery. Acta Physiol (Oxf) [Internet]. 2014;211:122–34. Available from: http://www.ncbi.nlm.nih.gov/pubmed/24528535

80. Prior SJ, Blumenthal JB, Katzel LI, Goldberg AP, Ryan AS. Increased skeletal muscle capillarization after aerobic exercise training and weight loss improves insulin sensitivity in adults with IGT. Diabetes Care [Internet]. 2014;37:1469–75. Available from: http://www.ncbi.nlm.nih.gov/pubmed/24595633

81. Scribbans TD, Edgett BA, Vorobej K, Mitchell AS, Joanisse SD, Matusiak JBL, et al. Fibre-specific responses to endurance and low volume high intensity interval training: striking similarities in acute and chronic adaptation. PLoS One [Internet]. 2014;9:e98119. Available from: http://www.ncbi.nlm.nih.gov/pubmed/24901767

82. Gliemann L, Olesen J, Biensø RS, Schmidt JF, Akerstrom T, Nyberg M, et al. Resveratrol modulates the angiogenic response to exercise training in skeletal muscles of aged men. Am J Physiol Heart Circ Physiol [Internet]. 2014;307:H1111-9. Available from: http://www.ncbi.nlm.nih.gov/pubmed/25128170

83. Larsen MS, Vissing K, Thams L, Sieljacks P, Dalgas U, Nellemann B, et al. Erythropoietin administration alone or in combination with endurance training affects neither skeletal muscle morphology nor angiogenesis in healthy young men. Exp Physiol [Internet]. 2014;99:1409–20. Available from: http://www.ncbi.nlm.nih.gov/pubmed/25128327

84. Gliemann L, Gunnarsson TP, Hellsten Y, Bangsbo J. 10-20-30 training increases performance and lowers blood pressure and VEGF in runners. Scand J Med Sci Sports [Internet]. 2015;25:e479-89. Available from: http://www.ncbi.nlm.nih.gov/pubmed/25439558

85. Cocks M, Shaw CS, Shepherd SO, Fisher JP, Ranasinghe A, Barker TA, et al. Sprint interval and moderate-intensity continuous training have equal benefits on aerobic capacity, insulin sensitivity, muscle capillarisation and endothelial eNOS/NAD(P)Hoxidase protein ratio in obese men. J Physiol [Internet]. 2016;594:2307–21. Available from: http://www.ncbi.nlm.nih.gov/pubmed/25645978

86. Gliemann L, Buess R, Nyberg M, Hoppeler H, Odriozola A, Thaning P, et al. Capillary growth, ultrastructure remodelling and exercise training in skeletal muscle of essential hypertensive patients. Acta Physiol (Oxf) [Internet]. 2015;214:210–20. Available from: http://www.ncbi.nlm.nih.gov/pubmed/25846822

87. Vigelsø A, Gram M, Wiuff C, Andersen JL, Helge JW, Dela F. Six weeks' aerobic retraining after two weeks' immobilization restores leg lean mass and aerobic capacity but does not fully rehabilitate leg strength in young and older men. J Rehabil Med [Internet]. 2015;47:552–60. Available from: http://www.ncbi.nlm.nih.gov/pubmed/25898161

88. Walton RG, Finlin BS, Mula J, Long DE, Zhu B, Fry CS, et al. Insulin-resistant subjects have normal angiogenic response to aerobic exercise training in skeletal muscle, but not in adipose tissue. Physiol Rep [Internet]. 2015;3:1–15. Available from: http://www.ncbi.nlm.nih.gov/pubmed/26038468

89. Prior SJ, Goldberg AP, Ortmeyer HK, Chin ER, Chen D, Blumenthal JB, et al. Increased Skeletal Muscle Capillarization Independently Enhances Insulin Sensitivity in Older Adults After Exercise Training and Detraining. Diabetes [Internet]. 2015;64:3386–95. Available from: http://www.ncbi.nlm.nih.gov/pubmed/26068543

90. Lewis MI, Fournier M, Wang H, Storer TW, Casaburi R, Kopple JD. Effect of endurance and/or strength training on muscle fiber size, oxidative capacity, and capillarity in hemodialysis patients. J Appl Physiol [Internet]. 2015;119:865–71. Available from: http://www.ncbi.nlm.nih.gov/pubmed/26183484

91. Montero D, Cathomen A, Jacobs RA, Flück D, de Leur J, Keiser S, et al. Haematological rather than skeletal muscle adaptations contribute to the increase in peak oxygen uptake induced by moderate endurance training. J Physiol [Internet]. 2015;593:4677–88. Available from: http://www.ncbi.nlm.nih.gov/pubmed/26282186

92. Baum O, Gübeli J, Frese S, Torchetti E, Malik C, Odriozola A, et al. Angiogenesis-related ultrastructural changes to capillaries in human skeletal muscle in response to endurance exercise. J Appl Physiol [Internet]. 2015;119:1118–26. Available from: http://www.ncbi.nlm.nih.gov/pubmed/26384412

93. Boushel R, Gnaiger E, Larsen FJ, Helge JW, González-Alonso J, Ara I, et al. Maintained peak leg and pulmonary VO2 despite substantial reduction in muscle mitochondrial capacity. Scand J Med Sci Sports [Internet]. 2015;25 Suppl 4:135–43. Available from: http://www.ncbi.nlm.nih.gov/pubmed/26589127

94. Munters LA, Loell I, Ossipova E, Raouf J, Dastmalchi M, Lindroos E, et al. Endurance Exercise Improves Molecular Pathways of Aerobic Metabolism in Patients With Myositis. Arthritis Rheumatol (Hoboken, NJ) [Internet]. 2016;68:1738–50. Available from: http://www.ncbi.nlm.nih.gov/pubmed/26867141

95. Nyberg M, Fiorenza M, Lund A, Christensen M, Rømer T, Piil P, et al. Adaptations to Speed Endurance Training in Highly Trained Soccer Players. Med Sci Sports Exerc [Internet]. 2016;48:1355–64. Available from: http://www.ncbi.nlm.nih.gov/pubmed/26885636

96. Stray-Gundersen J, Howden EJ, Parsons DB, Thompson JR. Neither Hematocrit Normalization nor Exercise Training Restores Oxygen Consumption to Normal Levels in Hemodialysis Patients. J Am Soc Nephrol [Internet]. 2016;27:3769–79. Available from: http://www.ncbi.nlm.nih.gov/pubmed/27153927

97. Tzanis G, Philippou A, Karatzanos E, Dimopoulos S, Kaldara E, Nana E, et al. Effects of High-Intensity Interval Exercise Training on Skeletal Myopathy of Chronic Heart Failure. J Card Fail [Internet]. Elsevier Inc.; 2017;23:36–46. Available from: http://www.ncbi.nlm.nih.gov/pubmed/27327970

98. Morville T, Rosenkilde M, Munch-andersen T, Andersen PR, K KKJRG, Helbo S, et al. Repeated Prolonged Exercise Decreases Maximal Fat Oxidation in Older Men. 2017;308–16.

99. Zinner C, Morales-Alamo D, Ørtenblad N, Larsen FJ, Schiffer TA, Willis SJ, et al. The Physiological Mechanisms of Performance Enhancement with Sprint Interval Training Differ between the Upper and Lower Extremities in Humans. Front Physiol [Internet]. 2016;7:426. Available from: http://www.ncbi.nlm.nih.gov/pubmed/27746738

100. Iepsen UW, Munch GDW, Rugbjerg M, Rinnov AR, Zacho M, Mortensen SP, et al. Effect of endurance versus resistance training on quadriceps muscle dysfunction in COPD: a pilot study. Int J Chron Obstruct Pulmon Dis [Internet]. 2016;11:2659–69. Available from: https://www.dovepress.com/effect-of-endurance-versus-resistance-training-on-quadriceps-muscle-dy-peer-reviewed-article-COPD

101. Bonafiglia JT, Edgett BA, Baechler BL, Nelms MW, Simpson CA, Quadrilatero J, et al. Acute upregulation of PGC-1α mRNA correlates with training-induced increases in SDH activity in human skeletal muscle. Appl Physiol Nutr Metab [Internet]. 2017;42:656–66. Available from: http://www.ncbi.nlm.nih.gov/pubmed/28177701

102. Valdivieso P, Toigo M, Hoppeler H, Flück M. T/T homozygosity of the tenascin-C gene polymorphism rs2104772 negatively influences exercise-induced angiogenesis. PLoS One [Internet]. 2017;12:e0174864. Available from: http://www.ncbi.nlm.nih.gov/pubmed/28384286

103. Mueller SM, Gehrig SM, Petersen JA, Frese S, Mihaylova V, Ligon-Auer M, et al. Effects of endurance training on skeletal muscle mitochondrial function in Huntington disease patients. Orphanet J Rare Dis [Internet]. Orphanet Journal of Rare Diseases; 2017;12:184. Available from: http://www.ncbi.nlm.nih.gov/pubmed/29258585

104. van der Zwaard S, Brocherie F, Kom BLG, Millet GP, Deldicque L, van der Laarse WJ, et al. Adaptations in muscle oxidative capacity, fiber size, and oxygen supply capacity after repeated-sprint training in hypoxia combined with chronic hypoxic exposure. J Appl Physiol [Internet]. 2018;124:1403–12. Available from: http://www.ncbi.nlm.nih.gov/pubmed/29420150

105. Tan R, Nederveen JP, Gillen JB, Joanisse S, Parise G, Tarnopolsky MA, et al. Skeletal muscle fiber-type-specific changes in markers of capillary and mitochondrial content after low-volume interval training in overweight women. Physiol Rep [Internet]. 2018;6:1–8. Available from: http://www.ncbi.nlm.nih.gov/pubmed/29484852

106. Raleigh JP, Giles MD, Islam H, Nelms M, Bentley RF, Jones JH, et al. Contribution of central and peripheral adaptations to changes in maximal oxygen uptake following 4 weeks of sprint interval training. Appl Physiol Nutr Metab [Internet]. 2018;43:1059–68. Available from: http://www.ncbi.nlm.nih.gov/pubmed/29733694

107. Mijwel S, Cardinale DA, Norrbom J, Chapman M, Ivarsson N, Wengström Y, et al. Exercise training during chemotherapy preserves skeletal muscle fiber area, capillarization, and mitochondrial content in patients with breast cancer. FASEB J [Internet]. 2018;32:5495–505. Available from: http://www.ncbi.nlm.nih.gov/pubmed/29750574

108. Esposito F, Mathieu-Costello O, Wagner PD, Richardson RS. Acute and chronic exercise in patients with heart failure with reduced ejection fraction: evidence of structural and functional plasticity and intact angiogenic signalling in skeletal muscle. J Physiol [Internet]. 2018;596:5149–61. Available from: http://www.ncbi.nlm.nih.gov/pubmed/30192995

109. Richardson RS, Wagner H, Mudaliar SRD, Saucedo E, Henry R, Wagner PD. Exercise adaptation attenuates VEGF gene expression in human skeletal muscle. Am J Physiol Heart Circ Physiol [Internet]. 2000;279:H772-8. Available from: http://www.ncbi.nlm.nih.gov/pubmed/10924077

110. Mortensen SP, Winding KM, Iepsen UW, Munch GW, Marcussen N, Hellsten Y, et al. The effect of two exercise modalities on skeletal muscle capillary ultrastructure in individuals with type 2 diabetes. Scand J Med Sci Sports [Internet]. 2019;29:360–8. Available from: http://www.ncbi.nlm.nih.gov/pubmed/30480353

111. Hesketh K, Shepherd SO, Strauss JA, Low DA, Cooper RJ, Wagenmakers AJM, et al. Passive heat therapy in sedentary humans increases skeletal muscle capillarization and eNOS content but not mitochondrial density or GLUT4 content. Am J Physiol Heart Circ Physiol [Internet]. 2019;317:H114–23. Available from: http://www.ncbi.nlm.nih.gov/pubmed/31074654

112. Svedenhag J, Henriksson J, Juhlin-Dannfelt A. Beta-adrenergic blockade and training in human subjects: effects on muscle metabolic capacity. Am J Physiol. 1984;247:E305–11.

113. de Moraes R, Van Bavel D, Gomes M de B, Tibiriçá E. Effects of non-supervised low intensity aerobic excise training on the microvascular endothelial function of patients with type 1 diabetes: a non-pharmacological interventional study. BMC Cardiovasc Disord [Internet]. BMC Cardiovascular Disorders; 2016;16:23. Available from: http://dx.doi.org/10.1186/s12872-016-0191-9

114. Scarpelli M, Belardinelli R, Tulli D, Provinciali L. Quantitative analysis of changes occurring in muscle vastus lateralis in patients with heart failure after low-intensity training. Anal Quant Cytol Histol [Internet]. 1999;21:374–80. Available from: http://www.ncbi.nlm.nih.gov/pubmed/10560519

115. Lithell H, Krotkiewski M, Kiens B, Wroblewski Z, Holm G, Strömblad G, et al. Non-response of muscle capillary density and lipoprotein-lipase activity to regular training in diabetic patients. Diabetes Res [Internet]. 1985;2:17–21. Available from: http://www.ncbi.nlm.nih.gov/pubmed/3995872

116. Mitchell EA, Martin NRW, Turner MC, Taylor CW, Ferguson RA. The combined effect of sprint interval training and postexercise blood flow restriction on critical power, capillary growth, and mitochondrial proteins in trained cyclists. J Appl Physiol. 2019;126:51–9.

117. Scott SN, Shepherd SO, Hopkins N, Dawson EA, Strauss JA, Wright DJ, et al. Home-hit improves muscle capillarisation and eNOS/NAD(P)Hoxidase protein ratio in obese individuals with elevated cardiovascular disease risk. J Physiol [Internet]. 2019;597:4203–25. Available from: http://www.ncbi.nlm.nih.gov/pubmed/31218680

118. Merlet AN, Messonnier LA, Coudy-Gandilhon C, Béchet D, Gellen B, Rupp T, et al. Beneficial effects of endurance exercise training on skeletal muscle microvasculature in sickle cell disease patients. Blood [Internet]. 2019;134:2233–41. Available from: http://www.ncbi.nlm.nih.gov/pubmed/31742587

119. Blervaque L, Passerieux E, Pomiès P, Catteau M, Héraud N, Blaquière M, et al. Impaired training-induced angiogenesis process with loss of pericyte-endothelium interactions is associated with an abnormal capillary remodelling in the skeletal muscle of COPD patients. Respir Res [Internet]. Respiratory Research; 2019;20:278. Available from: http://www.ncbi.nlm.nih.gov/pubmed/31806021

120. Fiorenza M, Gunnarsson TP, Ehlers TS, Bangsbo J. High-intensity exercise training ameliorates aberrant expression of markers of mitochondrial turnover but not oxidative damage in skeletal muscle of men with essential hypertension. Acta Physiol (Oxf) [Internet]. 2019;225:e13208. Available from: http://www.ncbi.nlm.nih.gov/pubmed/30339318

121. Islam H, Bonafiglia JT, Del Giudice M, Pathmarajan R, Simpson CA, Quadrilatero J, et al. Repeatability of training-induced skeletal muscle adaptations in active young males. J Sci Med Sport [Internet]. Sports Medicine Australia; 2021;24:494–8. Available from: http://www.ncbi.nlm.nih.gov/pubmed/33160857

122. Bonafiglia JT, Islam H, Preobrazenski N, Ma A, Deschenes M, Erlich AT, et al. Examining interindividual differences in select muscle and whole-body adaptations to continuous endurance training. Exp Physiol [Internet]. 2021;106:2168–76. Available from: https://doi.org/10.1113/EP089421

123. Leuchtmann AB, Mueller SM, Aguayo D, Petersen JA, Ligon-Auer M, Flück M, et al. Resistance training preserves high-intensity interval training induced improvements in skeletal muscle capillarization of healthy old men: a randomized controlled trial. Sci Rep [Internet]. 2020;10:6578. Available from: http://www.ncbi.nlm.nih.gov/pubmed/32313031

124. Olsen LN, Hoier B, Hellsten Y, Hogan M, Hepple R. Angiogenic potential is reduced in skeletal muscle of aged women. 2020;0:1–16.

125. Skattebo Ø, Capelli C, Rud B, Auensen M, Calbet JAL, Hallén J. Increased oxygen extraction and mitochondrial protein expression after small muscle mass endurance training. Scand J Med Sci Sports [Internet]. 2020;30:1615–31. Available from: http://www.ncbi.nlm.nih.gov/pubmed/32403173

126. Skattebo Ø, Bjerring AW, Auensen M, Sarvari SI, Cumming KT, Capelli C, et al. Blood volume expansion does not explain the increase in peak oxygen uptake induced by 10 weeks of endurance training. Eur J Appl Physiol [Internet]. Springer Berlin Heidelberg; 2020;120:985–99. Available from: http://www.ncbi.nlm.nih.gov/pubmed/32172291

127. Gliemann L, Rytter N, JØrgensen TS, Piil P, Carter H, Nyberg M, et al. The Impact of Lower Limb Immobilization and Rehabilitation on Angiogenic Proteins and Capillarization in Skeletal Muscle. Med Sci Sports Exerc. 2021;53:1797–806.

128. Perez-Gomez J, Rytter N, Mandrup C, Egelund J, Stallknecht B, Nyberg M, et al. Menopausal transition does not influence skeletal muscle capillary growth in response to cycle training in women. J Appl Physiol. 2021;131:369–75.

129. Tryfonos A, Tzanis G, Pitsolis T, Karatzanos E, Koutsilieris M, Nanas S, et al. Exercise Training Enhances Angiogenesis-Related Gene Responses in Skeletal Muscle of Patients with Chronic Heart Failure. Cells [Internet]. 2021;10. Available from: http://www.ncbi.nlm.nih.gov/pubmed/34440684

130. Almquist NW, Eriksen HB, Wilhelmsen M, Hamarsland H, Ing S, Ellefsen S, et al. No Differences Between 12 Weeks of Block- vs. Traditional-Periodized Training in Performance Adaptations in Trained Cyclists. Front Physiol [Internet]. 2022;13:837634. Available from: http://www.ncbi.nlm.nih.gov/pubmed/35299664
